# Catalogue of the ants (Hymenoptera, Formicidae) of Bulgaria

**DOI:** 10.3897/zookeys.62.430

**Published:** 2010-10-14

**Authors:** Albena Lapeva-Gjonova, Vera Antonova, Alexander G. Radchenko, Maria Atanasova

**Affiliations:** 1 Sofia University, Department of Zoology and Anthropology, 8 Dragan Tzankov blvd., 1164 Sofia, Bulgaria; 2 Institute of Biodiversity and Ecosystem Researches, Bulgarian Academy of Sciences, 2 Gagarin str., 1113 Sofia, Bulgaria; 3 Museum and Institute of Zoology of Polish Academy of Sciences, 64 Wilcza str., 00-679, Warsaw, Poland

**Keywords:** ants, Formicidae, Bulgaria, catalogue, new records, fauna, conservation status, geographical distribution

## Abstract

The present catalogue of the ants (Hymenoptera, Formicidae) of Bulgaria is made on a base of critical reconsideration of literature (covering the period from 1892 till 2009 and part of 2010) as well as on examination of the authors‘ and several museum‘s collections. A lot of data were omitted in the previous Bulgarian monograph on ants, lots of new data were recently added and many important additions and alterations were made due to taxonomic revisions of Eurasian Formicidae during the last three decades. Two new species are reported for the country [Temnothorax graecus (Forel, 1911) and Temnothorax cf. korbi (Emery, 1924)].

This catalogue contains a list of 163 ant species belonging to 40 genera of 6 subfamilies now known from Bulgaria. Synonyms and information on the previously reported names in relevant publications are given. Known localities of the species are grouped by geographic regions. Maps with concrete localities or regions for each species were prepared. The conservation status of 13 ant species is given as they are included in IUCN Red List of Threatened Species and Bulgarian Biodiversity Act. In comparison with adjacent Balkan regions the ant fauna of Bulgaria is quite rich and its core is composed of South European elements.

## Introduction

The Bulgarian myrmecofauna is among the richest of the local faunas of Southern Europe. This is a result of the high diversity of natural habitats, variability of climate and orography, and by the complicated history of the origin of the ant fauna of this relatively small territory. Despite a comparatively large number of publications concerning myrmecological investigation in this country, including monographic synthesizing, a lot of data has been omitted by previous authors and much new data has been added recently. To make a complete synopsis of all the existing information we have summarized it and compiled modern Catalogue of Bulgarian ants.

Early studies of the myrmecofauna of Bulgaria started more than 100 years ago, when Auguste Forel (1892) recorded 54 ant species from various regions of the country: the city of Sofia, Rila Mountain, Rhodopi Mountains, Stara Planina Mountains, and South Black Sea coast. Three new species were also described in this paper: Temnothorax bulgaricus (as Leptothorax), Cardiocondyla bulgarica (as Cardiocondyla elegans Emery var. *bulgarica*), and Cardiocondyla stambuloffii.

The most significant contribution to the fauna and biology of the ants in Bulgaria over the past century was made by Dr. Neno Atanassov, whose first papers were published in the 1930s (Atanassov 1934, 1936), where he reported 57 species. After World War II his monographic work on the fauna and biology of ants of the Vitosha Mountain was published (Atanassov 1952). A couple of other faunistic papers for the Bulgarian territory (Atanassov and Vassileva 1976), particularly of South Dobrudzha (Atanassov and Bulgurkov 1955), Petrich basin (Atanassov 1964), Lozenska Planina Mountain (Vassilev and Evtimov 1973), Rachene river valley (Vassilev 1984) made important additions to our knowledge of the Bulgarian myrmecofauna. Further contributions were also provided by (Wesselinoff (1936, 1968, 1972, 1979), (Atanassov (1957, 1965, 1982), Urbański (1975), (Baroni Urbani (1977, 1978).

In the 1960s and 1980s myrmecological investigations in Bulgaria were concerned mostly with the distribution and biology of red wood ants (Otto et al. 1962, Ronketi and Penev 1966, Wesselinoff 1967, 1972, 1973, 1974, 1979, Keremidchiev et al. 1971, 1972, Bobev 1972, 1973, Atanassov 1974, 1979, 1983, 1990, Gateva 1974, 1975, 1978, Vatov and Bobev 1976, Vesselinov 1981).

Over the last three decades many important additions and alterations to the taxonomy of Eurasian Formicidae, that also include data on Bulgarian ants, have been published (Seifert 1983, 1988a, b, 1990, 1991, 1992, 1995, 1996, 1997a, b, 2000a, b, c, 2002a, b, 2003a, b, 2005, 2006, Radchenko 1989, 1991, 1995a, b, 1997a, b, 2000, 2001, 2007, Radchenko and Elmes 2003, 2004, Schlick-Steiner et al. 2003, 2006a, Seifert and Schultz 2009, Seifert et al. 2009). Two new species have been described from the territory of the country: Lasius balcanicus Seifert, 1988 and Lasius nitidigaster Seifert, 1996.

The first attempt to summarize data on Balkan ants, including the Bulgarian fauna, was made by Agosti and Collingwood (1987a, b). They reported 112 species for Bulgaria based on data from their own and some other Museum collections, as well as on literature data. However, much of the data from these two papers needs revision and correction.

By far, the most comprehensive information on the Bulgarian myrmecofauna is contained in the book “Fauna of Bulgaria. Hymenoptera, Formicidae” by Atanassov and Dlusskij (1992). It includes data on the taxonomy, distribution and ecology of 111 ant species from 36 genera and 4 subfamilies, with identifications keys of all taxa. However, some species, recorded previously for Bulgaria (Sadil 1952, Barrett 1970, Seifert 1983, 1988a, b, 1990, Agosti and Collingwood 1987a, Buschinger et al. 1988) are omitted from this monograph.

Not surprisingly, a lot of essential additions to Bulgarian ant fauna have been published since 1992. These include faunistic and ecological data of the myrmecofauna of Sofia city and its environs (Antonova 2004, 2005, Lapeva-Gjonova and Atanasova 2004, Antonova and Penev 2006, 2008), Rhodopi Mountains (Lapeva-Gjonova 2004a, Lapeva-Gjonova in press (a)), Strandzha Mountain (Antonova et al. in press), Plana Mountain (Vagalinski and Lapeva-Gjonova in press). Beside this, many ant species have been recorded for Bulgaria for the first time (Seifert 1992, Buschinger and Douwes 1993, Radchenko 1994, Seifert 1995, 1997a, Czechowska et al. 1998, Seifert 2000a, b, Markó and Csősz 2002, Csősz and Seifert 2003, Radchenko and Antonova 2004, Stankiewicz and Antonova 2005, Csősz and Markó 2005, Steiner et al. 2005, Seifert 2006, Antonova 2009, Bezděčka and Bezděčková 2009, Csősz and Schulz 2010, Lapeva-Gjonova in press (b)).

As a result, we now include in the List of Bulgarian fauna 163 ant species belonging to 40 genera of 6 subfamilies.

## Materials and order of the Catalogue

The Catalogue we present here is based on the investigation of ants collected personally by the authors in various regions of Bulgaria during last decade, as well as on the material, preserved in the following Museums and Institutions: private collection of A. Lapeva-Gjonova, Faculty of Biology, Sofia University; private collection of V. Antonova, Institute of Biodiversity and Ecosystem Researches, Bulgarian Academy of Sciences, Sofia (IBER); Zoological Museum of the Moscow State University, Russia (ZMMU); Museum and Institute of Zoology of Polish Academy of Sciences, Warsaw, Poland (MIZ); shmalhausen Institute of Zoology of Ukrainian National Academy of Sciences, Kiev, Ukraine (SIZK); National Museum of Natural History, Prague, Czech Republic (NMNHP). All the available literature was also surveyed and the data therein used and in some cases critically reviewed.

Dr. Neno Atanassov’s collection of ants is kept in the National History Museum in Sofia and includes a few thousands of mounted specimens. Unfortunately, the collection is almost useless as the specimens have no locality labels but only numbers.

This catalogue contains a list of ant species, currently known in Bulgaria. Synonyms, information on the previously reported names and localities are included. Two new species [Temnothorax graecus (Forel, 1911) and Temnothorax cf. korbi (Emery, 1924)] for the country are reported.

The arrangement of genera in the subfamilies in the catalog is given by tribes. The species within each genus are listed in alphabetical order. Actual names of genera and species, and their authors are consistent with the most recent world catalogues of ants (Bolton 2003, Bolton et al. 2006) or, in the case of subsequent publications, with the most recent publications of the relevant authors.

**Map 1.**
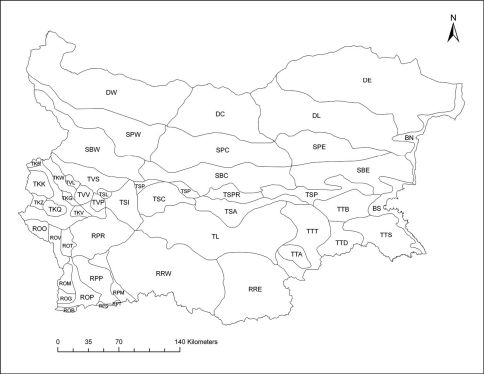
Geographical regions of Bulgaria [after Hubenov (1997) with minor changes and additions]

Publications by both Bulgarian and foreign authors concerning Bulgarian ants covering the period from 1892 till 2009 (2010, part) have been considered. After the actual name of the species, a list of synonyms and names used in relevant publications is given. Known localities of the species are grouped by geographic regions and subregions, arranged in the order given by Hubenov (1997) (Map 1). We have introduced additional regions and/or subregions for localities, which are not adequately covered by that classification system. Some of the localities, given in the references with obsolete names in older references, have been updated here. A few of the names could not be associated with any city or geographical location in the country due to typographical errors or incorrect transliteration. We have cited them in an italic font in the text. Publications without any reference to particular localities in the country are included before all other localities as referring generally to Bulgaria. Notes with comments on taxonomic position and distribution are added for some species.

IUCN species categories are given by the last “1994 Red List Categories & Criteria (version 2.3)” (IUCN 2009). It incorporates changes as a result of comments from IUCN members and was adopted by the IUCN Council in December 1994. In the recent catalogue the global status category for a given taxon is included. The national status of all the species recorded from Bulgaria that are listed in the IUCN report need updating.

Maps with concrete localities or regions for each species have been prepared. Whenever the number of localities for a species is very low, the information for two or more species is combined on a common map. In some cases species have been reported without a specific locality and only region or subregion were noted. In these cases the species are assigned to the whole area (region or subregion).

## Catalogue

### Subfamily Amblyoponinae

#### Genus Amblyopone

Erichson, 1842

##### Amblyopone denticulata

(Roger, 1859)

Amblyopone gheorghieffi  Forel, 1892

###### Records

(Map 2): **Bulgaria** (Agosti and Collingwood 1987a); **Eastern Stara Planina Mts:** Sliven [Forel 1892 (as A. *gheorghieffi*), Baroni Urbani 1978]; **Surnena Sredna Gora Mts:** the slopes of Sredna Gora Mts by Stara Zagora (Atanassov and Dlusskij 1992).

##### Amblyopone impressifrons

(Emery, 1869)

###### Records

(Map 2): **Thracian Lowland**: Svilengrad district, **Krupnik-Sandanski-Petrich Valley**: Kulata (Atanassov and Dlusskij 1992).

### Subfamily Ponerinae

Emery, 1893

#### Genus Cryptopone

Emery, 1893

##### Cryptopone ochracea

(Mayr, 1855)

###### Records

(Map 3): **Eastern Danubian Plain**; **Thracian Lowland**; **Krupnik-Sandanski-Petrich Valley**:Petrich (Atanassov and Dlusskij 1992).

#### Genus Hypoponera

Santschi, 1938

##### Hypoponera eduardi

(Forel, 1894)

###### Records

(Map 4): **Krupnik-Sandanski-Petrich Valley**: Petrich district (Atanassov and Dlusskij 1992).

###### Notes:

Atanassov and Dlusskij (1992) on page 69 noted that this species is widespread in Southern Europe but is absent from Bulgaria, while on a page 71 they report it from the Petrich region.

##### Hypoponera punctatissima

(Roger, 1859)

###### Records

(Map 4): **Eastern Danubian Plain**: Silistra (Atanassov and Dlusskij 1992); **Sofia Basin**: Sofia [Atanassov 1936 (as Ponera punctatissima), Atanassov and Dlusskij 1992]; **Sredna Gora Mts** (Atanassov and Dlusskij 1992); **Sushtinska** **Sredna gora Mts:** Hisarya [Atanassov 1936 (as Ponera punctatissima)]; **Krupnik-Sandanski-Petrich Valley**: Kulata, Melnik (Atanassov and Dlusskij 1992).

#### Genus Ponera

Latreille, 1804

##### Ponera coarctata

(Latreille, 1802)

###### Records

(Map 5): **Bulgaria** (Emery 1914, Taylor 1967, Agosti and Collingwood 1987a, Atanassov and Dlusskij 1992); **Eastern Danubian Plain**: Razgrad, Ruse, Shumen (Wesselinoff 1936); **Sofia Basin**: Sofia (Atanassov 1936, Lapeva-Gjonova and Atanasova 2004, Antonova 2005, Antonova and Penev 2006, 2008); **Strandzha** **Mt.** (Wesselinoff 1936); **Belasitsa Mt.** (Atanassov 1964, Hubenov et al. 1998); **Krupnik-Sandanski-Petrich Valley**: Petrich (Atanassov 1964); **Western Rhodopi Mts:** Asenovgrad (Wesselinoff 1936), Peshtera (Lapeva-Gjonova in press (a)); **Eastern Rhodopi Mts:** Zvezdel vill. (Momchilgrad) (Lapeva-Gjonova 2004a); **Northern Black Sea coast**: Varna district (Wesselinoff 1936); **Southern Black Sea coast**: Rezovo (Atanassov 1936).

###### Notes:

See Ponera testacea.

##### Ponera testacea

Emery, 1895

###### Records

(Map 5): **Krupnik-Sandanski-Petrich Valley**: Melnik, Melnik river valley, Rozhen (Csősz and Seifert 2003).

###### Notes:

Csősz and Seifert (2003) revived the name Ponera coarctata var. testacea from synonymy and raised it to species. Ponera testacea is a more thermophilous species than Ponera coarctata and seems to be widely distributed in Southern Europe. We are pretty sure that many of the previous records of Ponera coarctata for Bulgaria might belong to Ponera testacea.

### Subfamily Proceratiinae

#### Genus Proceratium

Roger, 1863

##### Proceratium melinum

(Roger, 1860)

###### Records

(Map 6): **Eastern Danubian Plain**: Dobrudzha (Atanassov and Dlusskij 1992); **Thracian Lowland**: Svilengrad (Atanassov and Dlusskij 1992); **Krupnik-Sandanski-Petrich Valley**: Petrich, Sandanski (Atanassov and Dlusskij 1992); **Southern Black Sea coast**: Burgas [(Forel 1895 (as Proceratium europaeum), Baroni Urbani 1977, Baroni Urbani and De Andrade 2003, Atanassov and Dlusskij 1992].

##### Proceratium numidicum

Santschi, 1912

###### Records:

**Bulgaria** (Agosti and Collingwood 1987a).

###### Notes:

This species was omitted by Atanassov and Dlusskij (1992). It was described from Tunisia and reported for Albania (Brown 1958), Turkey (Egridir) (Baroni Urbani 1977) and Cyprus (Baroni Urbani and De Andrade 2003). Agosti and Collingwood (1987a) recorded this species without any comments on Bulgaria. The presence of Proceratium numidicumin Bulgaria needs confirmation.

### Subfamily Myrmicinae

#### Genus Pyramica

Roger, 1862

##### Pyramica baudueri

(Emery, 1875)

###### Records

(Map 6): **Eastern Rhodopi Mts:** near Shiroko pole vill. (Momchilgrad) (Bezděčka and Bezděčková 2009).

###### Notes:

After revision of the tribe Dacetini and proposed synonymisation of many generic names (e.g. Bolton 1999, 2000, 2003, Bolton et al. 2006) this species was considered to be in the genus Pyramica. However, recently, Baroni Urbani and De Andrade (2007) provided a new reassessment of the tribe Dacetini and formally synonymised Pyramica with Strumigenys F. Smith. This system is not fully accepted currently (2010) and, perhaps, needs additional revisions and we use here the system, proposed by (Bolton (2000, 2003).

#### Genus Manica

Jurine, 1807

##### Manica rubida

(Latreille, 1802)

###### Records

(Map 7): **Bulgaria** (Agosti and Collingwood 1987a); **Central Predbalkan**: Dermantsi vill. (Lukovit) [Atanassov 1934 (as Myrmica rubida), Atanassov and Dlusskij 1992]; **Central Stara Planina Mts:** Botev peak (Ray hut) [Atanassov 1936 (as Myrmica rubida)]; **Vitosha Mt.** [Atanassov 1936 (as Myrmica rubida), Atanassov and Dlusskij 1992]; **Podbalkan Basins**: Karlovo gorge [Atanassov 1934 (as Myrmica rubida)]; **Osogovska Planina Mt.:** Ruen peak [Atanassov 1934 (as Myrmica rubida)], Kamen peak [Atanassov 1936 (as Myrmica rubida)]; **Rila Mt.:** Elenin peak, Rilska river valley (Forel 1892), Borovets [Atanassov 1934 (as Myrmica rubida)], Musala peak [Atanassov 1936 (as Myrmica rubida), Atanassov and Dlusskij 1992]; **Slavianka Mt.:** Alibotush reserve (Antonova 2009); **Western Rhodopi Mts:** Peshtera, Smolyan, Dospat, Rakitovo, Batak (Lapeva-Gjonova in press (a)); **Southern Black Sea coast**: Maslen nos (Atanassov 1934, Atanassov and Dlusskij 1992).

###### Notes:

The locality Maslen nos (20 m asl) is the lowest one for the species in Bulgaria (Atanassov and Dlusskij 1992). We did not examine correspondent material, but based on the current knowledge of the distribution and ecology of this species we may expect that this record is based on mislabeling or misidentification.

#### Genus Myrmica

Latreille, 1804

##### Myrmica constricta

Karavajev, 1934

###### Records

(Map 8): **Krupnik-Sandanski-Petrich Valley**: Melnik (Seifert et al. 2009).

##### Myrmica gallienii

Bondroit, 1920

Myrmica limanica  Arnoldi, 1934

###### Records

(Map 8): **Bulgaria** (Agosti and Collingwood 1987a, Seifert 1988a: the Balkans); **Western Predbalkan**: Rachene river valley [Vassilev 1984 (as Myrmica limanica)]; **Vitosha Mt.** (Atanassov and Dlusskij 1992).

##### Myrmica hellenica

Finzi, 1926

Myrmica rugulososcabrinodis  Karawajew, 1929

###### Records

(Map 8): **Thracian Lowland**: Maritsa river valley (near by Haskovo), Betova (Seifert 1988a, Seifert et al. 2009); **Sofia Basin**: Sofia [Radchenko and Antonova 2004, Antonova 2005, Antonova and Penev 2006, 2008 (all as Myrmica rugulososcabrinodis)]; **Krupnik-Sandanski-Petrich Valley**: Melnik, Sandanski [Seifert 1988a, Radchenko and Antonova 2004 (as Myrmica rugulososcabrinodis), Seifert et al. 2009]; **Northern Black Sea coast**: Obzor (Seifert et al. 2009); **Southern Black Sea coast**: Burgas (Seifert et al. 2009).

###### Notes:

Very recently Seifert et al. (2009) synonymised Myrmica rugulososcabrinodis with Myrmica hellenica. This species was omitted by Atanassov and Dlusskij (1992).

##### Myrmica lobicornis

Nylander, 1846

###### Records

(Map 8): **Bulgaria** (Agosti and Collingwood 1987a, Seifert 1988a: entire Europe excluding Italy and Greece); **Central Stara Planina Mts:** Karlovo Balkan (Atanassov 1936, Atanassov and Dlusskij 1992); **Vitosha Mt.** (Atanassov 1936, 1952, Atanassov and Dlusskij 1992); **Plana Mt.:** Plana vill., Tsiganka peak (Pasarel vill.) (Vagalinski and Lapeva-Gjonova in press); **Osogovska Planina Mt.**, **Belasitsa Mt.** (Atanassov and Dlusskij 1992); **Rila Mt.:** Elenin peak, Rilska river valley (Forel 1892, Atanassov and Dlusskij 1992); **Pirin Mt.** (Atanassov and Dlusskij 1992); **Slavianka Mt.:** Alibotush reserve (Antonova 2009); **Rhodopi Mts** (Atanassov and Dlusskij 1992); **Western Rhodopi Mts:** Peshtera (Lapeva-Gjonova in press (a)).

##### Myrmica lonae

Finzi, 1926

###### Records

(Map 9): **Bulgaria** (Seifert 2005); **Sofia Basin**: Sofia, surroundings of Sofia (Antonova and Penev 2006, 2008); **Lozenska Planina Mt.** near German monastery (Antonova and Penev 2008); **Strandzha Mt.** (Antonova et al. in press); **Western Rhodopi Mts:** Dobrostan (Seifert 2000b), Rakitovo (Lapeva-Gjonova in press (a)).

###### Notes:

Since Myrmica lonae was for a long time considered to be a junior synonym of Myrmica sabuleti, some old records of the latter species (see below) may belong to Myrmica lonae.

##### Myrmica rubra

(Linnaeus, 1758)

Myrmica laevinodis  Nylander, 1846

###### Records

(Map 9): **Bulgaria** (Emery 1914, Agosti and Collingwood 1987a, Atanassov and Dlusskij 1992); **Central Stara Planina Mts:** along Kostinya river (Teteven Balkan) [Atanassov 1936 (as Myrmica rubra laevinodis)]; **Eastern Stara Planina Mts:** Sliven [Forel 1892 (as Myrmica laevinodis)]; **Sofia Basin**: Sofia (Lapeva-Gjonova and Atanasova 2004, Antonova and Penev 2006, 2008); **Vitosha Mt.** [Atanassov 1952 (as Myrmica laevinodis)]: Knyazhevo [Forel 1892 (as Myrmica laevinodis)], Zheleznitsa vill. [Atanassov 1936 (as Myrmica rubra laevinodis)]; **Plana Mt.:** Plana vill., Kokalyane monastery (Kokalyane vill.), Ivanova mogila peak (Alino vill.) (Vagalinski and Lapeva-Gjonova in press); **Western Rhodopi Mts:** Batak, Velingrad, Rakitovo, Peshtera, Smolyan (Lapeva-Gjonova in press (a)).

##### Myrmica ruginodis

Nylander, 1846

###### Records

(Map 10): **Bulgaria** (Emery 1914, Agosti and Collingwood 1987a, Atanassov and Dlusskij 1992); **Sofia Basin**: Sofia (Lapeva-Gjonova and Atanasova 2004, Antonova 2005, Antonova and Penev 2006, 2008), surroundings of Sofia near Vladaya vill. (Antonova and Penev 2006, 2008); **Vitosha Mt.** (Atanassov 1936, 1952); **Plana Mt.:** Kokalyane monastery (Kokalyane vill.), Astronomical observatory (between Plana vill. and Dolni Okol vill.), east of Zheleznitsa vill., under Muhchel peak (Dolni Okol vill.), Tsiganka peak (Pasarel vill.), Peyova buka hut (Pasarel vill.) (Vagalinski and Lapeva-Gjonova in press); **Belasitsa Mt.:** at the foot of Belasitsa Mt. (Atanassov 1964); **Krupnik-Sandanski-Petrich Valley**: plain by Strumeshnitsa river (Atanassov 1964); **Rila Mt.:** Rila monastery (Forel 1892), Borovets (Atanassov 1936); **Slavianka Mt.:** Alibotush reserve (Antonova 2009); **Western Rhodopi Mts:** Devin, Chepelare (Lapeva-Gjonova in press (a)).

##### Myrmica rugulosa

Nylander, 1849

###### Records

(Map 10): **Bulgaria** (Agosti and Collingwood 1987a, Seifert 1988a: north of 41 o N in the Balkans); **Western Stara Planina Mts:** above Berkovitsa (Atanassov and Dlusskij 1992); **Central Stara Planina Mts:** under Vezhen peak (Atanassov and Dlusskij 1992); **Eastern** **Stara Planina Mts:** Chumerna (Atanassov and Dlusskij 1992); **Sofia Basin**: Sofia (Lapeva-Gjonova and Atanasova 2004, Antonova 2005, Antonova and Penev 2006, 2008), surroundings of Sofia near Vladaya vill. (Antonova and Penev 2006, 2008); **Lozenska Planina Mt**. near German monastery (Antonova and Penev 2008); **Thracian Lowland**: Pazardzhik (Forel 1892); **Rila Mt.:** Raduil, Rila monastery (Atanassov and Dlusskij 1992); **Western Rhodopi Mts:** Asenovgrad (Atanassov and Dlusskij 1992), Peshtera, Chepelare, Smolyan (Atanassov and Dlusskij 1992); **Eastern** **Rhodopi Mts:** Momchilgrad (Atanassov and Dlusskij 1992).

##### Myrmica sabuleti

Meinert, 1861

###### Records

(Map 11): **Bulgaria** (Agosti and Collingwood 1987a, Seifert 2005); **Western Predbalkan**: Rachene river valley (Vassilev 1984); **Stara Planina Mts** (Atanassov and Dlusskij 1992); **Sofia Basin**: Sofia (Antonova 2004, 2005, Lapeva-Gjonova and Atanasova 2004, Antonova and Penev 2006, 2008); **Vitosha** **Mt.** (Atanassov 1952, Atanassov and Dlusskij 1992, Hubenov et al. 1998); **Plana Mt.:** Tsiganka peak (Pasarel vill.), Bukov dol loc. (Pasarel vill.), Pasarel vill., Alino vill., Peyova buka hut (Pasarel vill.)(Vagalinski and Lapeva-Gjonova in press); **Lozenska Planina Mt.:** near German monastery (Antonova and Penev 2008); **Strandzha Mt.** (Antonova et al. in press); **Rila Mt.**, **Pirin Mt.**, **Rhodopi Mts** (Atanassov and Dlusskij 1992); **Western Rhodopi Mts:** Batak, Dospat, Rakitovo (Lapeva-Gjonova in press (a)); **Eastern** **Rhodopi Mts:** Byal Izvor vill. (Arda), Zvezdel vill. (Momchilgrad), Malki Voden vill. (Madzharovo), Kokiche vill. (Kardzhali) (Lapeva-Gjonova 2004a); **South Bulgaria** (Seifert 1988a).

##### Myrmica scabrinodis

Nylander, 1846

###### Records

(Map 11): **Bulgaria** (Agosti and Collingwood 1987a, Seifert 1988a, Atanassov and Dlusskij 1992); **Central Predbalkan**: Dermantsi vill. (Lukovit) (Atanassov 1934); **Eastern Stara Planina Mts:** Sliven (Forel 1892); **Sofia Basin**: Sofia (Forel 1892, Lapeva-Gjonova and Atanasova 2004, Antonova 2005, Antonova and Penev 2006, 2008); **Vitosha Mt.** (Atanassov 1934, 1952); **Plana Mt.:** Pasarel vill. (Vagalinski and Lapeva-Gjonova in press); **Lozenska Planina** **Mt.** (Vassilev and Evtimov 1973), near German monastery (Antonova and Penev 2008); **Bakadzhik-Burgas district**: Aytos (Forel 1892); **Belasitsa Mt.:** slopes of Belasitsa Mt. (Atanassov 1964, Hubenov et al. 1998); **Western Rhodopi Mts:** Smolyan, Peshtera, Batak (Lapeva-Gjonova in press (a)).

##### Myrmica schencki

Viereck, 1903

###### Records

(Map 12): **Bulgaria** (Agosti and Collingwood 1987a, Seifert 1988a); **Stara Planina Mts** (Atanassov and Dlusskij 1992); **Sofia Basin**: Sofia (Lapeva-Gjonova and Atanasova 2004, Antonova 2005, Antonova and Penev 2006, 2008); surroundings of Sofia near Vladaya vill. (Antonova and Penev 2006, 2008); **Vitosha Mt.** (Atanassov 1952, Atanassov and Dlusskij 1992); **Plana Mt.:** Tsiganka peak (Pasarel vill.), Bukov dol loc. (Pasarel vill.), Pasarel vill., Alino vill., Turmachka neighbourhood (Plana vill.) (Vagalinski and Lapeva-Gjonova in press); **Lozenska Planina Mt**. near German monastery (Antonova and Penev 2008); **Krupnik-Sandanski-Petrich Valley**: west of Petrich, around Mitino vill. (Atanassov 1964, Hubenov et al. 1998); **Rila Mt.** (Atanassov and Dlusskij 1992); **Pirin Mt.** (Atanassov and Dlusskij 1992): Rozhen vill. (Seifert 2003a); **Rhodopi Mts** (Atanassov and Dlusskij 1992); **Western Rhodopi Mts:** Chepelare, Smolyan, Rakitovo (Lapeva-Gjonova in press (a)).

##### Myrmica slovaca

Sadil, 1952

Myrmica salina : Seifert 1988a, 2002a, Antonova 2005, Antonova and Penev 2006, 2008, *nec* Ruzsky, 1905

###### Records

(Map 12): **Sofia Basin**: Sofia [Antonova 2005, Antonova and Penev 2006, 2008 (all as Myrmica salina)]; **Strandzha Mt.:** *Tirnovo* [Seifert 2002a (as Myrmica salina)]; **Western Rhodopi Mts:** Bachkovo [Sadil 1952, Seifert 1988a (as Myrmica salina)].

###### Notes:

During the last decade Myrmica slovaca has been considered as a junior synonym of Myrmica salina Ruzsky, 1905, but very recently Radchenko and Elmes (2009) based on the finding of Ruzsky’s original type material of Myrmica salina showed that these two species are not conspecific and revived the name Myrmica slovaca from synonymy.

Despite the fact that one of the type localities of Myrmica slovaca is in Bulgaria, this species was omitted by Atanassov and Dlusskij (1992).

##### Myrmica specioides

Bondroit, 1918

Myrmica sancta  Karawajew, 1926
Myrmica bessarabica : Atanassov and Dlusskij 1992, *nec* Nasonov, 1889

###### Records

(Map 13): **Bulgaria** (Agosti and Collingwood 1987a); **Sofia Basin**: Sofia (Antonova 2005, Antonova and Penev 2006, 2008), near Vladaya vill. (Antonova and Penev 2008); **Lyulin Mt.** [Atanassov and Dlusskij 1992 (as Myrmica bessarabica)]; **Strandzha Mt.:** Malko Tarnovo (Seifert 1988a); **Rila Mt.:** Kostenets [Atanassov and Vassileva 1976 and Hubenov et al. 1998 (as Myrmica sancta)]; **Southern Black Sea coast**: Sozopol (Seifert 1988a).

###### Notes:

After examination of the “holotype” of Myrmica bessarabica Seifert (2002a) found that there is a discrepancy between Nasonov’s original description and the characteristics of this specimen, and proposed considering Myrmica bessarabica *incertae sedis* in the genus Myrmica, and revived the name Myrmica specioides from synonymy (see also Radchenko and Elmes 2004).

##### Myrmica sulcinodis

Nylander, 1846

###### Records

(Map 13): **Bulgaria** (Agosti and Collingwood 1987a, Seifert 1988a, Atanassov and Dlusskij 1992); **Western Stara Planina Mts:** Milanovo vill. (Vratsa) (Atanassov 1936); **Sofia Basin**: surroundings of Sofia near Vladaya vill. (Antonova 2004, Antonova and Penev 2006, 2008); **Lyulin Mt.** (Atanassov 1936); **Vitosha Mt.** (Atanassov 1952); **Plana Mt.:**Astronomical observatory (between Plana vill. and Dolni Okol vill.)(Vagalinski and Lapeva-Gjonova in press); **Belasitsa Mt.:** the slopes of Belasitsa (Atanassov 1964, Hubenov et al. 1998); **Krupnik-Sandanski-Petrich Valley**: Luda Mara river valley (Atanassov 1964); **Rila Mt.:** Elenin peak (Forel 1892), Kostenets (Atanassov 1936); **Western Rhodopi Mts:** Smolyan, Peshtera, Rakitovo (Lapeva-Gjonova in press (a)).

##### Myrmica vandeli

Bondroit, 1920

###### Records

(Map 13): **Vitosha Mt.:**above Selimitsa hut (Stankiewicz and Antonova 2005).

###### Notes:

The locality for this species in Bulgaria is the southernmost in Europe (Stankiewicz and Antonova 2005).

#### Genus Stenamma

Westwood, 1839

##### Stenamma debile

(Förster, 1850)

Stenamma westwoodi : Emery 1914, Atanassov 1964, Atanassov and Dlusskij 1992, Hubenov et al. 1998, Lapeva-Gjonova and Atanasova 2004, *nec* Westwood, 1839

###### Records

(Map 14): **Bulgaria** (Emery 1914); **Western Danubian Plain**: Vidin (Atanassov 1964); **Western Stara Planina Mts:** under Todorini Kukli peak (Atanassov and Dlusskij 1992), Chepan (Dragoman) (Borisova et al. 2005); **Eastern** **Stara Planina Mts:**Karandila, under Razboyna peak (Atanassov and Dlusskij 1992); **Sofia Basin**: Sofia [Lapeva-Gjonova and Atanasova 2004 (as Stenamma westwoodi), Antonova 2005, Antonova and Penev 2006, 2008]; **Plana Mt.:** Peyova buka hut (Pasarel vill.) (Vagalinski and Lapeva-Gjonova in press.); **Sushtinska Sredna Gora Mts:** under Bratiya peak (Atanassov and Dlusskij 1992); **Belasitsa Mt.** [Atanassov 1964 (as Stenamma westwoodi), Hubenov et al. 1998]; **Western Rhodopi Mts:** Asenovgrad (Atanassov and Dlusskij 1992), Peshtera (Lapeva-Gjonova in press (a)); **Eastern** **Rhodopi Mts:** Momchilgrad (Atanassov and Dlusskij 1992).

###### Notes:

According to DuBois (1993) Stenamma westwoodii is distributed only in Western Europe, so all previous records for it in Bulgaria should refer to Stenamma debile, which is a more widespread European species.

#### Genus Aphaenogaster

Mayr, 1853

##### Aphaenogaster epirotes

(Emery, 1895)

###### Records

(Map 15): **Eastern Danubian Plain**: Dobrich (Atanassov and Dlusskij 1992); **Eastern Rhodopi Mts:** Svirachi vill. (Ivaylovgrad), between Odrintsi and Svirachi vill. (Lapeva-Gjonova 2004a).

##### Aphaenogaster gibbosa

(Latreille, 1798)

###### Records

(Map 15): **Western** **Stara Planina Mts:** Chepan (Dragoman) (Borisova et al. 2005); **Krupnik-Sandanski-Petrich Valley**: west of Petrich, along Strumeshnitsa river (Atanassov 1964), Petrich (Hubenov et al. 1998); **Slavianka Mt.:** above Paril vill. (Atanassov and Dlusskij 1992); **Eastern Rhodopi Mts:** Momchilgrad, Ivaylovgrad (Atanassov and Dlusskij 1992); **Northern Black Sea coast**: Obzor vill. district (Atanassov and Dlusskij 1992).

##### Aphaenogaster pallida

(Nylander, 1849)

###### Records

(Map 16): **Western** **Predbalkan**: Rachene river valley (Vassilev 1984).

**Notes**: This species is distributed in France, Canary Islands, Italy, Corsica, Sicily, Algeria, Morocco and Tunisia. The record of this species for Bulgaria is quite doubtful and needs confirmation.

##### Aphaenogaster subterranea

(Latreille, 1798)

###### Records

(Map 16): **Bulgaria** (Emery 1914, Agosti and Collingwood 1987a); **Western Predbalkan**: Rachene river valley (Vassilev 1984); **Eastern Stara Planina Mts:** Sliven (Forel 1892), Byala vill. (Sliven) (Atanassov 1936), Karandila (Atanassov and Dlusskij 1992); **Vitosha Mt.** (Atanassov 1952); **Bakadzhik-Burgas district**: Aytos (Forel 1892); **Strandzha Mt.** (Antonova et al. in press): Papia peak (Atanassov 1936, Atanassov and Dlusskij 1992); **Osogovska Planina** **Mt.:** Hisarlaka (Atanassov 1936, Atanassov and Dlusskij 1992); **Belasitsa Mt.:** at the foot of Belasitsa Mt. (Atanassov 1964, Atanassov and Dlusskij 1992, Hubenov et al. 1998); **Slavianka Mt.** (Atanassov and Dlusskij 1992); **Rhodopi Mts** (Atanassov and Dlusskij 1992); **Western** **Rhodopi Mts:** Smolyan (Lapeva-Gjonova in press (a)); **Eastern Rhodopi Mts:** Madzharovo district, Malko Popovo and Malki Voden vill. (Madzharovo municipality), between Dabovets and Kamilski dol vill. (Ivaylovgrad), between Odrintsi vill. and Svirachi vill. (Ivaylovgrad) (Lapeva-Gjonova 2004a); **Northern Black Sea coast**: Obzor vill. (Atanassov and Dlusskij 1992).

#### Genus Messor

Forel, 1890

##### Notes:

Taxonomy of the genus Messor, particularly in Europe, is poorly resolved and many taxonomic problems need further elaboration. Therefore (see below) we left all the species names recorded by previous authors without comments, but we stress that many of the records need confirmation.

##### Messor atanassovii

Atanassov, 1982

###### Records

(Map 17): **Thracian Lowland**: Zagore vill. (Stara Zagora), Belozem vill. (Plovdiv) (Atanassov 1982, Atanassov and Dlusskij 1992); **Strandzha Mt.** (Antonova et al. in press); **Mesta Valley**: Dolno Dryanovo (Atanassov and Dlusskij 1992); **Western** **Rhodopi Mts:** Asenovgrad (Atanassov 1982, Atanassov and Dlusskij 1992).

##### Messor barbarus

(Linnaeus, 1767)

Aphaenogaster (Messor) barbara  L. var. *barbaro-structor* Dalla Tore, 1893 (*nomen nudum*): Forel 1892.
Aphaenogaster (Messor) barbara  L. var. *sordida* Forel, 1892: Forel 1895.

###### Records

(Map 17): **Bulgaria** (Csősz and Markó 2005); **Thracian Lowland**: Pazardzhik (Forel 1892); **Bakadzhik-Burgas district**: Aytos (Forel 1892); **Dupnitsa Basin**: Dupnitsa (Forel 1892); **Northern** **Black Sea coast**: Kavarna (Markó and Csősz 2002); **Southern Black Sea coast**: Pomorie, Veselie vill. (Forel 1892), Burgas (Forel 1895).

###### Notes:

This species was omitted by Atanassov and Dlusskij (1992).

##### Messor caducus

(Motschoulsky, 1839)

Messor semirufus meridionalis : Atanassov 1936, 1964, Atanassov and Vassileva 1976, Hubenov et al. 1998, *nec* André, 1883

###### Records

(Map 18): **Bulgaria** (Atanassov and Dlusskij 1992); **Podbalkan Basins**: Sotirya vill. (Sliven) [Atanassov and Vassileva 1976 (as Messor semirufus meridionalis)]; **Thracian Lowland**: Harmanli [Atanassov and Vassileva 1976 and Hubenov et al. 1998 (as Messor semirufus meridionalis)]; **Ograzhden Mt.** [Atanassov and Vassileva 1976 (as Messor semirufus meridionalis)]; **Belasitsa Mt.** (Hubenov et al. 1998): east slopes of Belasitsa Mt. [Atanassov 1964 (as Messor semirufus meridionalis)]; **Krupnik-Sandanski-Petrich Valley**: Petrich [Atanassov and Vassileva 1976 (as Messor semirufus meridionalis)]; **Eastern Rhodopi Mts:** Kardzhali [Atanassov and Vassileva 1976 (as Messor semirufus meridionalis)]; **Northern Black Sea coast**: Obzor [Atanassov and Vassileva 1976 and Hubenov et al. 1998 (as Messor semirufus meridionalis)]; **Southern Black Sea coast**: Ropotamo river – Sozopol [Atanassov 1936 (as Messor semirufus var. meridionalis)].

##### Messor capitatus

(Latreille, 1798)

###### Records

(Map 18): **Bulgaria** (Csősz and Markó 2005); **Northern Black Sea coast**: Kavarna, Kaliakra (Markó and Csősz 2002).

##### Messor concolor

Santschi, 1927

###### Records

(Map 19): **Bulgaria** (Agosti and Collingwood 1987a); **Thracian Lowland**: Klokotnitsa (Schlick-Steiner et al. 2006a).

###### Notes:

This species was omitted by Atanassov and Dlusskij (1992).

##### Messor oertzeni

Forel, 1910

Messor oertzeni  Forel var. *amphigea* Forel, 1911: Atanassov 1934, 1964

###### Records

(Map 19): **Bulgaria** (Agosti and Collingwood 1987a); **Ihtimanska Sredna Gora Mts:** Benkovski peak (Atanassov 1934); **Krupnik-Sandanski-Petrich Valley**: Petrich (Atanassov 1934), above Marino pole vill. [Atanassov 1964 (as Messor oertzeni var. amphigea)]; **Ograzhden Mt.**, **Belasitsa Mt.** [Atanassov 1964 (as Messor oertzeni var. amphigea)]; **Slavianka Mt.** (Atanassov and Dlusskij 1992); **Western Rhodopi Mts** (Atanassov and Dlusskij 1992); **Eastern Rhodopi Mts:** Senoklas vill. (Madzharovo), Svirachi vill. (Ivaylovgrad) (Lapeva-Gjonova 2004a); **Northern Black Sea coast**: Evksinograd palace (Atanassov and Dlusskij 1992).

##### Messor structor

(Latreille, 1798)

Messor muticus  Nylander, 1849
Messor barbarus varrialei  Emery, 1921
Messor clivorum  Ruzsky, 1905
Messor rufitarsis  Fabricius, 1804

###### Records

(Map 20): **Bulgaria** [Agosti and Collingwood 1987a (as Messor muticus), Atanassov and Dlusskij 1992]; **Western Predbalkan**:Rachene river valley [Atanassov and Vassileva 1976 (as Messor clivorum), Vassilev 1984]; **Central Predbalkan**:Dermantsi vill. (Lukovit) [Atanassov 1936 (as Messor barbarus varrialei and Messor structor rufitarsis), Atanassov and Vassileva 1976 (as Messor rufitarsis romanus)]; **Western Stara Planina Mts:** Milanovo vill., Lakatnik station [Atanassov 1934, 1936 (as Messor barbarus varrialei and Messor structor rufitarsis), Atanassov and Vassileva 1976 (as Messor clivorum)], Vrattsata loc., Zgorigrad vill. [Atanassov and Vassileva 1976 (as Messor clivorum)], Vratsa [Hubenov et al. 1998 (as Messor clivorum)], Chepan Mt. (Dragoman) (Borisova et al. 2005); **Zemen Gorge**: Skakavitsa waterfall, Zemen station [Atanassov 1936 (as Messor barbarus varrialei and Messor structor rufitarsis)], Zemen (Schlick-Steiner et al. 2006a); **Sofia Basin**: surroundings of Sofia (Antonova and Penev 2006); **Lyulin Mt.** (Vassilev and Evtimov 1973); **Vitosha Mt.** [Hubenov et al. 1998 (as Messor clivorum)]: Boyana [Atanassov 1934 (as Messor barbarus structor var. mutica), 1952 (as Messor rufitarsis)]; **Plana Mt.:** Pasarel vill., Peyova buka hut (Pasarel vill.), Grobat peak (Vagalinski and Lapeva-Gjonova in press); **Podbalkan Basins**: Rose valley [Atanassov et al. 1955 (as Messor rufitarsis)]; **Ihtimanska Sredna Gora**: Benkovski peak [Atanassov 1936 (as Messor barbarus varrialei and Messor structor rufitarsis)]; **Lozenska Planina Mt.** (Vassilev and Evtimov 1973); north of Pasarel vill. (Antonova and Penev 2008); **Thracian Lowland**: Pazardzhik (Forel 1892), Plovdiv (Forel 1911), Harmanli [Urbanski 1975 (as Messor rufitarsis)]; **Bakadzhik-Burgas district**: Aytos (Forel 1892), Mandra lake [Atanassov and Vassileva 1976 (as Messor clivorum)]; **Osogovska Planina Mt.:** Hisarlaka [Atanassov 1936 (as Messor barbarus varrialei and Messor structor rufitarsis)]; **Ograzhden Mt.** [Atanassov 1964 (as Messor barbarus varrialei and Messor structor rufitarsis)]; **Belasitsa Mt.:** at the foot of Belasitsa Mt. [Atanassov 1964 (as Messor barbarus varrialei and Messor structor rufitarsis)]; **Krupnik-Sandanski-Petrich Valley**: Kresna gorge [Atanasov 1936 (as Messor structor rufitarsis)], surroundings of Parvomay vill., along Strumeshnitsa river [Atanassov 1964 (as Messor barbarus varrialei)], Petrich [Atanassov and Vassileva 1976 (as Messor rufitarsis romanus)]; **Slavianka Mt.:** Petrovo vill. [Atanassov and Vassileva 1976 (as Messor rufitarsis romanus)]; **Western Rhodopi Mts:** Asenovgrad (Forel 1892), Smilyan vill. (Smolyan) (Hlaváč et al. 2007); **Eastern Rhodopi Mts:** Dedets vill. (Zlatograd), Byal Izvor vill. (Arda), Momchilgrad, Madzharovo, between Dabovets and Kamilski dol vill. (Ivaylovgrad), Odrintsi vill. (Ivaylovgrad), Padalo vill. (Krumovgrad) (Lapeva-Gjonova 2004a), Daskalovo (Schlick-Steiner et al. 2006a); **Northern** **Black Sea coast**: Evksinograd palace [Atanassov 1936 (as Messor barbarus varrialei and Messor rufitarsis)], Balchik (Schlick-Steiner et al. 2006a); **Southern** **Black Sea coast**: Nesebar (Barrett 1970), Mandra lake (Burgas) [Atanassov and Vassileva 1976 (as Messor rufitarsis romanus)].

#### Genus Pheidole

Westwood, 1839

##### Pheidole pallidula

(Nylander, 1849)

###### Records

(Map 21): **Bulgaria** (Agosti and Collingwood 1987a, Csősz and Markó 2005, Atanassov and Dlusskij 1992); **Western Predbalkan**: Rachene river valley (Vassilev 1984); **Central Predbalkan**: Dermantsi vill. (Lukovit) [Atanassov 1934, 1936 (as Pheidole pallidula arenarum var. orientalis)]; **Western Stara Planina Mts:** Lakatnik station [Atanassov 1934, 1936 (as Pheidole pallidula arenarum var. orientalis)]; **Central Stara Planina Mts:** Zhaltets peak [Atanassov 1934, 1936 (as Pheidole pallidula arenarum var. orientalis)]; **Eastern Stara Planina Mts:** Sliven (Forel 1892); **Zemen Gorge**: Skakavitsa waterfall [Atanassov 1936 (as Pheidole pallidula arenarum var. orientalis)]; **Podbalkan Basins**: Rose valley [Atanassov et al. 1955 (as Pheidole pallidula arenarum var. orientalis)]; **Strandzha Mt.:** Ropotamo river (Atanassov 1934); **Dupnitsa Basin**: Dupnitsa (Forel 1892); **Rila Mt.:** Rila (Forel 1892); **Krupnik-Sandanski-Petrich Valley**: Kresna, Kresna gorge [Atanassov 1934, 1936 (as Pheidole pallidula arenarum var. orientalis)]; west of Petrich [Atanassov 1964 (as Pheidole pallidula arenarum var. orientalis)]; **Ograzhden Mt.** [Atanassov 1964 (as Pheidole pallidula arenarum var. orientalis)]; **Belasitsa Mt.:** at the foot of Belasitsa Mt. [Atanassov 1964 (as Pheidole pallidula arenarum var. orientalis)]; **Western** **Rhodopi Mts:** Asenovgrad (Forel 1892); **Eastern Rhodopi Mts:** Dedets vill. (Zlatograd), Malko Popovo vill. (Madzharovo), Madzharovo, between Dabovets and Kamilski dol vill. (Ivaylovgrad), between Odrintsi vill. and Svirachi vill. (Ivaylovgrad), Svirachi vill. (Ivaylovgrad), Padalo vill. (Krumovgrad) (Lapeva-Gjonova 2004a); **Northern Black Sea coast**: Evksinograd palace [Atanassov 1936 (as Pheidole pallidula arenarum var. orientalis)], Kavarna (Markó and Csősz 2002); **Southern Black Sea coast**: Sozopol (Forel 1892), Burgas (Forel 1895), near Ropotamo river (Atanassov 1934).

#### Genus Myrmecina

Curtis, 1829

##### Myrmecina graminicola

(Latreille, 1802)

Myrmecina latreillei  Curtis, 1829

###### Records

(Map 22): **Bulgaria** (Emery 1914, Agosti and Collingwood 1987a); **Western Predbalkan**: Belogradchik (Atanassov and Dlusskij 1992); **Sofia Basin**: Sofia, surroundings of Sofia (Lapeva-Gjonova and Atanasova 2004, Antonova 2005, Antonova and Penev 2006, 2008); **Plana Mt.:** Pasarel vill. (Vagalinski and Lapeva-Gjonova in press); **Thracian Lowland**: Svilengrad (Atanassov and Vassileva 1976, Atanassov and Dlusskij 1992, Hubenov et al. 1998); **Krupnik-Sandanski-Petrich Valley**: Sandanski (Atanassov and Vassileva 1976, Atanassov and Dlusskij 1992, Hubenov et al. 1998); **Pirin Mt.:** Mechkul vill. (Atanassov and Dlusskij 1992); **Western Rhodopi Mts:** Peshtera (Lapeva-Gjonova in press (a)); **Southern Black Sea coast**: Burgas [Forel 1895 (as Myrmecina latreillei)], Tsarevo (Atanassov and Vassileva 1976, Atanassov and Dlusskij 1992).

#### Genus Crematogaster

Lund, 1831

##### Crematogaster auberti

Emery, 1869

###### Records

(Map 23): **Krupnik-Sandanski-Petrich Valley**: Kamenitsa vill. (Lapeva-Gjonova in press (b)).

##### Crematogaster schmidti

(Mayr, 1853)

Cremastogaster scutellaris var. christowitchii  Forel, 1892

###### Records

(Map 23): **Bulgaria** (Agosti and Collingwood 1987a, Atanassov and Dlusskij 1992); **Western Stara Planina Mts:** Lakatnik station (Atanassov 1936); **Eastern** **Stara Planina Mts:** Sliven [Forel 1892 (as Crematogaster scutellaris)]; **Podbalkan Basins**: Rose valley (Atanassov et al. 1955); **Thracian Lowland**: Pazardzhik [Forel 1892 (as Crematogaster scutellaris var. christotwitchii)]; **Bakadzhik-Burgas district**: Aytos [Forel 1892 (as Crematogaster scutellaris var. christotwitchii)]; **Strandzha Mt.** (Antonova et al. in press): Papia peak [Atanassov 1934 (as Crematogaster scutellaris var. christotwitchii)]; **Belasitsa Mt.** (Atanassov 1964); **Krupnik-Sandanski-Petrich Valley**: west of Petrich, along Strumeshnitsa river (Atanassov 1964); **Slavianka Mt.:** St. Vrach – Petrovo vill. (Atanassov 1936); **Western** **Rhodopi Mts:** Asenovgrad [Forel 1892 (as Crematogaster scutellaris var. christotwitchii)], Dospat (Lapeva-Gjonova in press (a)); **Eastern Rhodopi Mts:** Zhelezino vill. (Ivaylovgrad), Madzharovo municipality, Malko Popovo vill. (Madzharovo), Malki Voden vill. (Madzharovo), between Dabovets and Kamilski dol vill. (Ivaylovgrad), Odrintsi vill. (Ivaylovgrad), between Odrintsi and Svirachi vill. (Ivaylovgrad), Kokiche vill. (Kardzhali), Meden buk vill. (Ivaylovgrad) (Lapeva-Gjonova 2004a); **Southern Black Sea coast**: Veselie vill. [Forel 1892 (as Crematogaster scutellaris var. christotwitchii)], Burgas [Forel 1895 (as Crematogaster scutellaris)], Rezovo [Atanassov 1934 (as Crematogaster scutellaris and Crematogaster scutellaris var. christotwitchii)].

###### Notes:

According to Atanassov and Dlusskij (1992) the records of Forel (1892) and Atanassov (1934) for Crematogaster scutellaris should be refer to Crematogaster schmidti.

##### Crematogaster scutellaris

(Olivier, 1792)

###### Records:

**Bulgaria** (Agosti and Collingwood 1987a).

###### Notes:

This species was omitted by Atanassov and Dlusskij (1992).

##### Crematogaster sordidula

(Nylander, 1849)

###### Records

(Map 23): **Bulgaria** (Agosti and Collingwood 1987a, Atanassov and Dlusskij 1992, Csősz and Markó 2005); **Eastern** **Stara Planina Mts:** Sliven (Forel 1892); **Bakadzhik-Burgas district**: Aytos (Forel 1892); **Krupnik-Sandanski-Petrich Valley**: Petrich plain (Atanassov 1964); **Western Rhodopi Mts:** Asenovgrad (Forel 1892); **Eastern Rhodopi Mts:** Beli Plast vill. (Kardzhali), Madzharovo municipality, Malko Popovo vill. (Madzharovo), between Dabovets and Kamilski dol vill. (Ivaylovgrad), Svirachi vill. (Ivaylovgrad) (Lapeva-Gjonova 2004a); **Northern Black Sea coast**: Kavarna (Markó and Csősz 2002).

#### Genus Monomorium

Mayr, 1855

##### Monomorium pharaonis

(Linnaeus, 1758)

###### Records

(Map 24): **Eastern Danubian Plain**: Ruse, Shumen (Atanassov and Dlusskij 1992); **Sofia Basin**: Sofia (Atanassov 1965, Atanassov and Dlusskij 1992); **Thracian Lowland**: Svilengrad (Atanassov 1965), Plovdiv (Atanassov and Dlusskij 1992); **Strandzha Mt.:** Malko Tarnovo (Atanassov and Dlusskij 1992); **Northern** **Black Sea coast**: Varna (Atanassov 1965, Atanassov and Dlusskij 1992), Obzor vill. (Atanassov and Dlusskij 1992); **Southern Black Sea coast**: Burgas, Tsarevo (Atanassov 1965, Atanassov and Dlusskij 1992), Sozopol, Ahtopol (Atanassov and Dlusskij 1992).

#### Genus Solenopsis

Westwood, 1840

##### Solenopsis fugax

(Latreille, 1798)

###### Records

(Map 25): **Bulgaria** [Emery 1914, Agosti and Collingwood 1987a, Atanassov and Dlusskij 1992 (as Diplorhoptrum fugax)]; **Western Predbalkan**: Rachene river valley [Vassilev 1984 (as Diplorhoptrum fugax)]; **Central Predbalkan**: Dermantsi vill. (Lukovit) (Atanassov 1934); **Western Stara Planina Mts:** Milanovo vill. (Vratsa) (Atanassov 1934); **Sofia Basin**: Sofia [Forel 1892, Atanassov 1936, Antonova 2004, 2005, Lapeva-Gjonova and Atanasova 2004 (as Diplorhoptrum fugax); Antonova and Penev 2006, 2008], surroundings of Sofia (Antonova and Penev 2006); **Vitosha Mt.** (Atanassov 1936, 1952): Dragalevtsi (Atanassov 1934); **Podbalkan Basins**: Rose valley (Atanassov et al. 1955); **Ihtimanska Sredna Gora Mts:** Benkovski peak (Atanassov 1936); **Lozenska Planina Mt.** (Vassilev and Evtimov 1973); **Osogovska Planina Mt.:** Hisarlaka (Kyustendil) (Atanassov 1936); **Krupnik-Sandanski-Petrich Valley**: west of Petrich, along Strumeshnitsa river (Atanassov 1964); **Western Rhodopi Mts:** Asenovgrad (Forel 1892), Peshtera (Atanassov 1934, Lapeva-Gjonova in press (a)); **Eastern** **Rhodopi Mts:** Zvezdel vill. (Momchilgrad), between Dabovets and Kamilski dol vill. (Ivaylovgrad), between Odrintsi and Svirachi vill. (Ivaylovgrad), Svirachi vill. (Lapeva-Gjonova 2004a); **Southern Black Sea coast**: Burgas (Forel 1895).

#### Genus Formicoxenus

(Nylander, 1846)

##### Formicoxenus nitidulus

(Nylander, 1846)

###### Records

(Map 26): **Central Predbalkan**: Dermantsi vill. (Lukovit) (Atanassov 1936); **Stara Planina Mts** (Atanassov and Dlusskij 1992); **Western Stara Planina Mts:** Zgorigrad vill. (Vratsa) (Atanassov 1936); **Vitosha Mt.** (Atanassov and Dlusskij 1992): Knyazhevo (Csősz and Markó 2005); **Osogovska Planina Mt.:** Hisarlaka (Atanassov 1936, Atanassov and Dlusskij 1992); **Belasitsa Mt.** (Atanassov and Vassileva 1976, Hubenov et al. 1998); **Slavianka Mt.** (Atanassov and Dlusskij 1992); **Rhodopi Mts** (Atanassov and Dlusskij 1992); **Western Rhodopi Mts:** Peshtera (Lapeva-Gjonova in press (a)).

###### Conservation Status:

Vulnerable A2c (IUCN).

#### Genus Harpagoxenus

Forel, 1893

##### Harpagoxenus sublaevis

(Nylander, 1849)

###### Records

(Map 27): **Slavianka Mt.:** Alibotush reserve (Antonova 2009).

###### Conservation Status:

Vulnerable A2c (IUCN).

#### Genus Leptothorax

Mayr, 1855

##### Leptothorax acervorum

(Fabricius, 1793)

###### Records

(Map 28): **Bulgaria** (Emery 1914, Agosti and Collingwood 1987a, Atanassov and Dlusskij 1992); **Central Stara Planina Mts:** Bratanitsa peak (Atanassov 1936); **Vitosha Mt.** (Atanassov 1952); **Plana Mt.:** Tsiganka peak (Pasarel vill.), Alino vill. (Vagalinski and Lapeva-Gjonova in press); **Rila Mt.:** Rila monastery (Forel 1892); **Western Rhodopi Mts:** Rakitovo, Peshtera, Batak (Lapeva-Gjonova in press (a)).

##### Leptothorax muscorum

(Nylander, 1846)

###### Records

(Map 28): **Bulgaria** (Agosti and Collingwood 1987a); **Stara Planina Mts** (Atanassov and Dlusskij 1992); **Vitosha Mt.** (Atanassov 1952, Atanassov and Dlusskij 1992, Hubenov et al. 1998); **Rila Mt.** (Atanassov and Dlusskij 1992); **Slavianka Mt.:** Alibotush reserve (Antonova 2009); **Rhodopi Mts** (Atanassov and Dlusskij 1992).

#### Genus Temnothorax

Mayr, 1861

##### Notes:

Till recently Temnothorax was considered as a junior synonym of Leptothorax, but Bolton (2003) has divided the latter genus to two: Leptothorax (which includes the former subgenus Leptothorax s. str.) and Temnothorax (which includes, among others, the former subgenus Myrafant M. R. Smith). Therefore all authors before 2003 and some after this date have placed Temnothorax species in the genus Leptothorax (except of Temnothorax recedens).

##### Temnothorax affinis

(Mayr, 1855)

###### Records

(Map 29): **Bulgaria** (Emery 1914, Agosti and Collingwood 1987a); **Western Stara Planina Mts:** Chepan (Dragoman) (Borisova et al. 2005); **Eastern Stara Planina Mts** (Atanassov and Dlusskij 1992); **Sofia Basin**: Sofia (Forel 1892, Antonova 2005, Antonova and Penev 2006, 2008), surroundings of Sofia (Atanassov and Dlusskij 1992); **Lyulin Mt.** (Atanassov and Dlusskij 1992); **Vitosha Mt.** (Atanassov 1952, Atanassov and Dlusskij 1992); **Plana Mt.:** Bukov dol loc. (Pasarel vill.), Peyova buka hut (Pasarel vill.), Tsiganka peak (Pasarel vill.), Pasarel vill., Alino vill. (Vagalinski and Lapeva-Gjonova in press); **Belasitsa Mt.**, **Pirin Mt.**, **Slavianka Mt.** (Atanassov and Dlusskij 1992); **Rhodopi Mts** (Atanassov and Dlusskij 1992); **Eastern Rhodopi Mts:** Beli Plast vill. (Kardzhali), Zvezdel vill. (Momchilgrad), Zhelezino vill. (Ivaylovgrad), between Dabovets and Kamilski dol vill. (Ivaylovgrad) (Lapeva-Gjonova 2004a).

##### Temnothorax bulgaricus

(Forel, 1892)

###### Records

(Map 29): **Bulgaria** (Agosti and Collingwood 1987a); **Eastern Stara Planina Mts:** Sliven (Forel 1892, Atanassov and Dlusskij 1992); **Zemen Gorge**: Zemen station (Atanassov and Vassileva 1976), Zemen (Hubenov et al. 1998); **Krupnik-Sandanski-Petrich Valley**: Petrich (Atanassov and Dlusskij 1992); **Western Rhodopi Mts:** Asenovgrad (Atanassov and Dlusskij 1992); **Northern Black Sea coast**: Obzor vill. (Atanassov and Dlusskij 1992).

##### Temnothorax clypeatus

(Mayr, 1853)

###### Records

(Map 30): **Bulgaria** (Agosti and Collingwood 1987a); **Zemen Gorge**: Zemen district (Atanassov and Dlusskij 1992); **Lyulin Mt.** (Atanassov and Dlusskij 1992); **Plana Mt.:** Pasarel vill. (Vagalinski and Lapeva-Gjonova in press); **Osogovska Planina Mt.:** slopes of Osogovska Planina Mt. by Kyustendil (Atanassov and Dlusskij 1992); **Krupnik-Sandanski-Petrich Valley**: west of Petrich, near by Strumeshnitsa river, around Parvomay vill. (Atanassov 1964, Hubenov et al. 1998, Atanassov and Dlusskij 1992); **Belasitsa Mt.:** at the foot of Belasitsa Mt. (Atanassov and Dlusskij 1992); **Northern Black Sea coast**: Obzor vill. (Atanassov and Vassileva 1976, Hubenov et al. 1998).

##### Temnothorax corticalis

(Schenck, 1852)

###### Records

(Map 30): **Bulgaria** (Agosti and Collingwood 1987a); **Vitosha Mt.:** Dragalevtsi monastery (Atanassov and Dlusskij 1992); **Krupnik-Sandanski-Petrich Valley**: west of Petrich, along Strumeshnitsa river (Atanassov 1964, Atanassov and Dlusskij 1992, Hubenov et al. 1998); **Northern Black Sea coast**: Balchik (Atanassov and Dlusskij 1992); **Southern Black Sea coast**: Tsarevo (Atanassov and Dlusskij 1992).

##### Temnothorax crassispinus

(Karavaiev, 1926)

###### Records

(Map 31): **Sofia Basin**: Sofia (Lapeva-Gjonova and Atanasova 2004, Antonova 2004); **Plana Mt.:** Pasarel vill., Bukov dol loc. (Pasarel vill.), Muhchel peak (Dolni okol vill.), Peyova buka hut (Pasarel vill.), Alino vill. (Vagalinski and Lapeva-Gjonova in press); **Western Rhodopi Mts:** Peshtera (Lapeva-Gjonova in press (a)); **Southern Bulgarian mountains**: [Seifert 1995 (as Leptothorax nylanderi slavonicus)].

###### Notes:

For many years Temnothorax crassispinus was considered as a junior synonym of Temnothorax nylanderi, but Radchenko (2000) showed that it is a good species, distributed mainly in the Eastern Europe including Bulgaria, while Temnothorax nylanderi is mainly West European species. Nevertheless, we recently found Temnothorax nylanderi in parks in Sofia. Therefore although most of the records of Temnothorax nylanderi for Bulgaria from before 2000 should be Temnothorax crassispinus, some of them might include also “true” Temnothorax nylanderi, this question may be definitely resolved only after investigation of the correspondent material.

##### Temnothorax graecus

(Forel, 1911)

###### Records

(Map 31): **Central Stara Planina Mts:** Gabrovo monastery (Gabrovo) (Antonova, MIZ collection, det. A. Schultz, unpublished data).

##### Temnothorax interruptus

(Schenck, 1852)

###### Records

(Map 31): **Western Predbalkan**: Belogradchik (Atanassov and Dlusskij 1992); **Western Stara Planina Mts:** Berkovitsa (Atanassov and Dlusskij 1992); **Eastern** **Stara Planina Mts:** Kotel (Atanassov and Dlusskij 1992); **Plana Mt.:** Kokalyane monastery (Kokalyane vill.) (Vagalinski and Lapeva-Gjonova in press); **Krupnik-Sandanski-Petrich Valley**: Petrich (Atanassov and Dlusskij 1992); **Northern Black Sea coast**: Obzor vill., Balchik (Atanassov and Dlusskij 1992).

##### Temnothorax korbi

(Emery, 1924) cf.

###### Records

(Map 32): **Southern Black Sea coast**: Ahtopol (Antonova, MIZ collection, det. A. Schultz, unpublished data).

##### Temnothorax melanocephalus

(Emery, 1870)

###### Records

(Map 32): **Bulgaria** (Emery 1922, Agosti and Collingwood 1987a); **Krupnik-Sandanski-Petrich Valley**:Petrich (Atanassov and Dlusskij 1992); **Rila Mt.:** Borovets (Emery 1914).

###### Notes:

Bolton (1995) and Bolton et al. (2006) erroneously considered Temnothorax melanocephalus as a junior synonym of Temnothorax tuberum, referring this synonymy to Casevitz-Weulersse (1990), but at the same time noted that Atanassov and Dlusskij (1992) considered Temnothorax melanocephalus as a good species. Since 1992 its status has not been formally changed by any subsequent authors and we consider it as a good species.

##### Temnothorax nadigi

(Kutter, 1925)

###### Records

(Map 32): **Western Rhodopi Mts:** Chervenata stena reserve (Dobrostan) (Czechowska et al. 1998).

##### Temnothorax nigriceps

(Mayr, 1855)

###### Records:

**Bulgaria** (Agosti and Collingwood 1987a).

###### Notes:

This species was omitted by Atanassov and Dlusskij (1992).

##### Temnothorax nylanderi

(Förster, 1850)

###### Records

(Map 32) definitely belonging to Temnothorax nylanderi: **Sofia Basin**: Sofia (Antonova 2005, Antonova and Penev 2006, 2008); near Vladaya vill. (Antonova and Penev 2008); **Lozenska Planina Mt.:** near German monastery (Antonova and Penev 2008); **Strandzha Mt.** (Antonova et al. in press).

The following records of Temnothorax nylanderi most likely mainly belong to Temnothorax crassispinus, but might include also Temnothorax nylanderi: **Bulgaria** (Emery 1914, Agosti and Collingwood 1987a); **Western Predbalkan**: Belogradchik (Atanassov and Dlusskij 1992); **Western Stara Planina Mts:** Vratsa (Atanassov and Dlusskij 1992); **Sofia Basin**: Sofia (Forel 1892, Atanassov and Dlusskij 1992); **Krupnik-Sandanski-Petrich Valley**: Petrich (Atanassov and Dlusskij 1992); **Northern Black Sea coast**: Byala vill. (Varna), Balchik (Atanassov and Dlusskij 1992); **Southern Black Sea coast**: Burgas (Forel 1895).

##### Temnothorax parvulus

(Schenck, 1852)

###### Records

(Map 33): **Bulgaria** (Agosti and Collingwood 1987a, Atanassov and Dlusskij 1992); **Western Stara Planina Mts:** Chepan Mt. (Dragoman) (Borisova et al. 2005); **Eastern Stara Planina Mts:** Sliven (Forel 1892); **Vitosha Mt.:** Knyazhevo (Forel 1892); **Plana Mt.:** Bukov dol loc. (Pasarel vill.) (Vagalinski and Lapeva-Gjonova in press).

##### Temnothorax recedens

(Nylander, 1856)

###### Records

(Map 33): **Bulgaria** (Agosti and Collingwood 1987a); **Eastern Stara Planina Mts** (Atanassov and Dlusskij 1992): Sliven (Forel 1892, Dlussky and Soyunov 1988); **Bakadzhik-Burgas district**:Aytos (Atanassov and Dlusskij 1992); **Krupnik-Sandanski-Petrich Valley**: Kresna (Dlussky and Soyunov 1988, Atanassov and Dlusskij 1992); **Northern Black Sea coast**: Balchik, Kavarna, Batova river valley, Kaliakra, Obzor vill. (Atanassov and Dlusskij 1992); **Southern** **Black Sea coast**: Pomorie (Forel 1892, Atanassov and Dlusskij 1992), Burgas, Tsarevo, Rezovo (Atanassov and Dlusskij 1992).

###### Conservation Status:

Lower Risk/least concern (IUCN).

##### Temnothorax saxonicus

(Seifert, 1995)

###### Records

(Map 34): **Bulgaria** (Seifert 1995); **Western Stara Planina Mts:** Tompsan (Seifert 2006); **Vitosha Mt.:** Chuypetlyovo (Seifert 2006); **Krupnik-Sandanski-Petrich Valley**: Kresna, Petrich (Seifert 2006); **Northern Black Sea coast**: Aladzha monastery (Seifert 2006).

###### Notes:

Seifert (1995) described Temnothorax saxonicus as an eastern subspecies of Temnothorax sordidulus, but later he (Seifert 2006) raised it to the species rank.

##### Temnothorax semiruber

(André, 1881)

Leptothorax rottenbergi var. balcanica  Santschi, 1909

###### Records

(Map 34): **Bulgaria** (Agosti and Collingwood 1987a, Atanassov and Dlusskij 1992); **Eastern Stara Planina Mts:** Sliven (Forel 1892); **Boboshevo-Simitli Valley**: Kocherinovo [Atanassov and Vassileva 1976 and Hubenov et al. 1998 (as Leptothorax rottenbergi var. balcanica)]; **Krupnik-Sandanski-Petrich Valley**: Kresna gorge [Atanassov 1936 (as Leptothorax rottenbergi var. balcanica)], Sandanski [Atanassov and Vassileva 1976 and Hubenov et al. 1998 (as Leptothorax rottenbergi var. balcanica)]; **Belasitsa Mt.:** slopes of Belasitsa Mt. [Atanassov 1964 (as Leptothorax rottenbergi var. balcanica)]; **Western** **Rhodopi Mts:** Asenovgrad (Forel 1892); **Eastern Rhodopi Mts:** Dedets vill. (Zlatograd), Byal Izvor vill. (Arda), Momchilgrad, Madzharovo (Lapeva-Gjonova 2004a).

##### Temnothorax sordidulus

(Müller, 1923)

###### Records

(Map 34): **Western Predbalkan**: Cherven bryag (Seifert 2006); **Western Stara Planina Mts:** Lakatnik, Dragoman, Tri ushi, Tompsan (Seifert 2006).

##### Temnothorax tauricus

Ruzsky, 1902

###### Records

(Map 35): **Bulgaria** (Radchenko 1994, 1995a); **Krupnik-Sandanski-Petrich Valley**:Petrich; **Western** **Rhodopi Mts:** Bagnovski monastery (**=**Bachkovski monastery) (materials at ZMMU, A. Radchenko).

##### Temnothorax tuberum

(Fabricius, 1775)

###### Records

(Map 35): **Bulgaria** (Agosti and Collingwood 1987a); **Sofia Basin**:Sofia (Antonova 2005, Antonova and Penev 2006, 2008), surroundings of Sofia near Vladaya vill. (Antonova and Penev 2006, 2008); **Thracian Lowland**: Pazardzhik (Forel 1892); **Rila Mt**. (Atanassov and Dlusskij 1992): Elenin peak (Forel 1892); **Pirin Mt.**, **Rhodopi Mts** (Atanassov and Dlusskij 1992).

##### Temnothorax unifasciatus

(Latreille, 1798)

Leptothorax tuberum r. unifasciatus var. unifasciatotuberum  Forel, 1892 (*nomen nudum* and unavailable name)
Leptothorax tuberum r. unifasciatus var. interruptotuberum  Forel, 1892 (*nomen nudum* and unavailable name)

###### Records

(Map 35): **Bulgaria** (Emery 1914, Agosti and Collingwood 1987a); **Central Predbalkan**: Dermantsi vill. (Lukovit) (Atanassov 1936); **Stara Planina Mts** (Atanassov and Dlusskij 1992); **Eastern Stara Planina Mts:** Sliven (Forel 1892); **Sofia Basin**: Sofia (Forel 1892, Antonova 2004, 2005, Lapeva-Gjonova and Atanasova 2004, Antonova and Penev 2006, 2008), surroundings of Sofia (Antonova and Penev 2006); **Vitosha Mt.** (Atanassov 1952, Atanassov and Dlusskij 1992); **Plana Mt.:** Alino vill. (Vagalinski and Lapeva-Gjonova in press); **Lozenska Planina Mt.:** near German monastery and north of Pasarel vill. (Antonova and Penev 2008); **Strandzha Mt.** (Antonova et al. in press); **Belasitsa Mt.** (Atanassov 1964, Atanassov and Dlusskij 1992, Hubenov et al. 1998); **Rila Mt.** (Forel 1892, Atanassov and Dlusskij 1992); **Rhodopi Mts** (Atanassov and Dlusskij 1992); **Western Rhodopi Mts:** Peshtera (Lapeva-Gjonova in press (a)); **Southern Black Sea coast**: Burgas (Forel 1892).

#### Genus Chalepoxenus

Menozzi, 1923

##### Chalepoxenus muellerianus

(Finzi, 1922)

###### Records

(Map 27): **Bulgaria** (Buschinger and Douwes 1993); **Western Rhodopi Mts:** Dobrostan hill (Buschinger et al. 1988).

###### Notes:

This species is not included in Fauna of Bulgaria (Atanassov and Dlusskij 1992).

###### Conservation Status:

Vulnerable D2 (IUCN).

#### Genus Myrmoxenus

Ruzsky, 1902

##### Myrmoxenus gordiagini

Ruzsky, 1902

###### Records

(Map 27): **Western Predbalkan**: Reselets vill. (Cherven Bryag) (Buschinger and Douwes 1993).

###### Conservation Status:

Vulnerable D2 (IUCN).

##### Myrmoxenus ravouxi

(André, 1896)

###### Records

(Map 27): **Krupnik-Sandanski-Petrich Valley**: Kresna [Buschinger and Douwes 1993 (as Epimyrma ravouxi)].

###### Conservation Status:

Vulnerable D2 (IUCN).

#### Genus Cardiocondyla

Emery, 1869

##### Cardiocondyla bulgarica

Forel, 1892

###### Records

(Map 36): **Bulgaria** [Agosti and Collingwood 1987a, Radchenko 1995b (as junior synonym of Cardiocondyla elegans)]; **Pirin Mt.:** Rozhen (Seifert 2003b); **Northern Black Sea coast**: Obzor vill. (Atanassov and Dlusskij 1992); **Southern** **Black Sea coast**: Pomorie, Sozopol (Forel 1892, Atanassov and Dlusskij 1992, Seifert 2003b), Arkutino (Atanassov and Dlusskij 1992).

##### Cardiocondyla elegans

Emery, 1869

###### Records

(Map 36): **Krupnik-Sandanski-Petrich Valley**: Sandanski (Atanassov and Dlusskij 1992); **Pirin Mt.:** Mechkul vill. (Simitli) (Atanassov and Dlusskij 1992), Melnik (Seifert 2003b); **Northern Black Sea coast**: Kavarna, Byala, the mouth of Batova river (Atanassov and Dlusskij 1992); **Southern** **Black Sea coast**: Nesebar, Primorsko (Atanassov and Dlusskij 1992).

##### Cardiocondyla nigra

Forel, 1905

###### Records:

**Bulgaria** [Agosti and Collingwood 1987a, Radchenko 1995b (as junior synonym of Cardiocondyla batesii Forel)].

###### Notes:

Agosti and Collingwood (1987a) recorded Cardiocondyla nigra for Bulgaria and Turkey (Thrace), and Radchenko (1995b) repeated this record (under the name Cardiocondyla batesii that he considered as a senior synonym of Cardiocondyla nigra). However, Seifert (2003b) regarded Cardiocondyla batesii and Cardiocondyla nigra as separate species. According to him, the first species is distributed in Iberian Peninsula and NW Africa [Agosti and Collingwood (1987a) recorded it also for Greece], and the latter – in the Iberian Peninsula, North Africa and Turkey. Most probably, both species have distributions that include Mediterranean areas and are absent from Bulgaria; their occurrence in this country needs confirmation.

##### Cardiocondyla stambuloffii

Forel, 1892

###### Records

(Map 36): **Bulgaria** (Agosti and Collingwood 1987a); **Eastern Stara Planina Mts:** Sliven (Hubenov et al. 1998); **Northern** **Black Sea coast**: Albena, St. St. Konstantin and Elena, Varna, Obzor vill. (Atanassov and Dlusskij 1992); **Southern Black Sea coast**: Burgas (Forel 1892, Pisarski 1962, Atanassov and Dlusskij 1992, Radchenko 1995b, Hubenov et al. 1998, Seifert 2003b), Pomorie, Sozopol (Forel 1892, Atanassov and Dlusskij 1992, Seifert 2003b), Tsarevo (Atanassov and Dlusskij 1992, Hubenov et al. 1998), Arkutino, Ahtopol (Atanassov and Dlusskij 1992).

#### Genus Anergates

Forel, 1874

##### Anergates atratulus

(Schenck, 1852)

###### Records

(Map 37): **Western Stara Planina Mts**, **Vitosha Mt.**, **Osogovska** **Planina Mt.**, **Rhodopi Mts**, **Northern Black Sea coast**, **Southern Black Sea coast** (Atanassov and Dlusskij 1992).

###### Conservation Status:

Vulnerable D2 (IUCN).

#### Genus Tetramorium

Mayr, 1855

##### Tetramorium caespitum

(Linnaeus, 1758)

###### Records

(Map 38): **Bulgaria** (Emery 1914, Agosti and Collingwood 1987a, Atanassov and Dlusskij 1992); **Western Predbalkan**: Rachene river valley (Vassilev 1984); **Central Predbalkan**: Dermantsi vill. (Lukovit) (Atanassov 1934, 1936); **Western Stara Planina Mts:** Vrattsata loc., Milanovo vill. (Atanassov 1934), Lakatnik station (Atanassov 1936), Chepan Mt. (Dragoman) (Borisova et al. 2005); **Central Stara Planina Mts:** Teteven (Atanassov 1934), Tsarichina peak (Atanassov 1936); **Eastern Stara Planina Mts:** Sliven (Forel 1892); **Zemen Gorge**: Skakavitsa vill., Zemen (Atanassov 1936), Zemen gorge (Lapeva-Gjonova 2004b); **Sofia Basin**: Boyana (Atanassov 1934); Pancharevo vill. (Atanassov 1936), Sofia (Forel 1892, Lapeva-Gjonova and Atanasova 2004, Antonova 2005, Antonova and Penev 2006, 2008), surroundings of Sofia near Vladaya vill. (Atanasov 1936, Antonova and Penev 2006, 2008); **Lyulin Mt.:**Mihaylovo district (Atanassov 1936); **Vitosha Mt.** (Atanassov 1952); **Plana Mt.:** Plana vill., Pasarel vill., Richov well loc. (Dolni okol vill.), Tsiganka peak (Pasarel vill.), Yovichina mogila peak (Popovyane vill.) (Vagalinski and Lapeva-Gjonova in press); **Podbalkan Basins**: Rose valley (Atanassov et al. 1955); **Lozenska Planina Mt.** (Vassilev and Evtimov 1973): German monastery (Atanassov 1936, Antonova and Penev 2008), north of Pasarel vill. (Antonova and Penev 2008); **Thracian Lowland**: Pazardzhik (Forel 1892, Atanassov 1936), Krichim (Atanassov 1936); **Bakadzhik-Burgas district**: Aytos (Forel 1892); **Strandzha Mt.** (Antonova et al. in press); **Ograzhden Mt.** (Atanassov 1964); **Belasitsa Mt.** (Atanassov 1964); **Krupnik-Sandanski-Petrich Valley**: west of Petrich (Atanassov 1964); **Dupnitsa Basin**: Dupnitsa; **Rila Mt**.: Rilska river valley (Forel 1892), Kostenets, Borovets (Atanassov 1936); **Slavianka Mt.** (Atanassov 1936); **Mesta Valley**: Petrelik vill. (Gotse Delchev) (Lapeva-Gjonova 2004b); **Western Rhodopi Mts:** Asenovgrad (Forel 1892), Peshtera (Atanassov 1936, Lapeva-Gjonova in press (a)), Batak, Devin, Smolyan, Rakitovo, Velingrad, Dospat (Lapeva-Gjonova in press (a)); **Eastern Rhodopi Mts:** Dedets vill. (Zlatograd), Byal Izvor vill. (Arda), Beli Plast vill. (Kardzhali), Kokiche vill. (Kardzhali), Momchilgrad, Madzharovo, Malko Popovo vill. (Madzharovo), between Dabovetz and Kamilski dol vill. (Ivaylovgrad), between Odrintsi and Svirachi vill. (Ivaylovgrad), Svirachi vill. (Ivaylovgrad) (Lapeva-Gjonova 2004a); **Southern Black Sea coast**: Pomorie, Burgas, Sozopol, Veselie vill. (Forel 1892), Maslen nos, Mandra lake (Atanassov 1934), Nesebar (Barrett 1970).

###### Notes:

According to Schlick-Steiner et al. (2006b) the Tetramorium caespitum/impurum complex comprises seven Palaearctic species. The taxonomy of the complex is not properly resolved and we assume that there are several cryptic species related to Tetramorium caespitum in Bulgaria.

##### Tetramorium chefketi

Forel, 1911

Tetramorium taurocaucasicum  Arnoldi, 1968
Tetramorium forte  Forel, 1904 (part., material from Balkans, East Europe, Caucasus and Middle Asia)
Tetramorium moravicum : Agosti and Collingwood, 1987a, b, misidentification, *nec* Kratochvil, 1941
Tetramorium forte : Agosti and Collingwood 1987a; Atanassov and Dlusskij 1992; Lapeva-Gjonova 2004a; Lapeva-Gjonova and Atanasova 2004; Antonova 2005; Borisova et al. 2005

###### Records

(Map 39): **Bulgaria** (Agosti and Collingwood 1987a); **Eastern Danubian Plain**: Ludogorie-Dobrudzha district (Atanassov and Dlusskij 1992); **Stara Planina Mts** (Atanassov and Dlusskij 1992); **Western Stara Planina Mts:** Chepan Mt. (Dragoman) (Borisova et al. 2005); **Sofia Basin**: Sofia [Lapeva-Gjonova and Atanasova 2004 and Antonova 2005 (as Tetramorium forte), Antonova and Penev 2006, 2008], surroundings of Sofia (Antonova and Penev 2006); **Vitosha Mt.** (Atanassov 1952, Atanassov and Dlusskij 1992); **Lozenska Planina Mt**.: near German monastery (Antonova and Penev 2008); **Thracian Lowland**: Harmanli [Atanassov and Vassileva 1976 (as Tetramorium taurocaucasicum)]; **Strandzha Mt.** (Atanassov and Dlusskij 1992, Antonova et al. in press); **Belasitsa Mt.** (Atanassov and Dlusskij 1992); **Boboshevo-Simitli Valley**: Kocherinovo, Blagoevgrad (Csősz et al. 2007); **Pirin Mt.** (Atanassov and Dlusskij 1992); **Rhodopi Mts** (Atanassov and Dlusskij 1992); **Eastern Rhodopi Mts:** Kokiche vill. (Kardzhali), Madzharovo municipality, Byal Izvor vill. (Arda), Beli Plast vill. (Kardzhali), Malko Popovo vill. (Madzharovo), between Dabovets and Kamilski dol vill. (Ivaylovgrad), between Odrintsi vill. and Svirachi vill. (Ivaylovgrad), Svirachi vill. (Lapeva-Gjonova 2004a); **Southern Black Sea coast**: Primorsko [Atanassov and Vassileva 1976, Hubenov et al. 1998 (as Tetramorium taurocaucasicum)], Maslen nos [Atanassov and Vassileva 1976 (as Tetramorium taurocaucasicum)], Burgas (Csősz et al. 2007).

###### Notes:

Agosti and Collingwood (1987a) recorded Tetramorium forte and Tetramorium moravicum from Bulgaria, but Atanassov and Dlusskij (1992) treated Tetramorium moravicum, as well as Tetramorium taurocaucasicum, as junior synonyms of Tetramorium forte. The complicate taxonomic history of this species was resolved recently by Csősz et al. 2007 (for some more details see also Radchenko et al. 1998, Czechowski et al. 2002, Güsten et al. 2006).

##### Tetramorium diomedeum

Emery, 1908

###### Record

(Map 39): **Southern Black Sea coast**: Ahtopol (Csősz and Schulz 2010).

##### Tetramorium ferox

Ruzsky, 1903

###### Records

(Map 39): **Bulgaria** (Agosti and Collingwood 1987a, Atanassov and Dlusskij 1992); **Podbalkan Basins**: Kalofer (Atanassov and Vassileva 1976); **Krupnik-Sandanski-Petrich Valley**: Sandanski (Atanassov and Vassileva 1976); **Western Rhodopi Mts:** Smilyan vill. (Hlaváč et al. 2007); **Eastern** **Rhodopi Mts:** Kardzhali (Csősz and Schulz 2010); **Southern Black Sea coast**: Dolno Ezerovo (Burgas) (Csősz and Schulz 2010).

##### Tetramorium hungaricum

(Röszler, 1935)

###### Records

(Map 40): **Bulgaria** (Agosti and Collingwood 1987a, Atanassov and Dlusskij 1992); **Central Predbalkan**: Dermantsi vill. (Lukovit) [Atanassov 1936 (as Tetramorium caespitum semilaeve)]; **Sofia Basin**: Sofia [Lapeva-Gjonova and Atanasova 2004 (as Tetramorium semilaeve), Antonova 2005, Antonova and Penev 2006, 2008]; **Plana Mt.:** Kokalyane vill. (Steiner et al. 2005); **Belasitsa Mt.:** at the foot of Belasitsa Mt. [Atanassov 1964 (as Tetramorium caespitum semilaeve)]; **Krupnik-Sandanski-Petrich Valley**: Kresna [Atanassov 1936 (as Tetramorium caespitum semilaeve)], around Mitino vill. [Atanassov 1964 (as Tetramorium caespitum semilaeve)]; Sandanski (Csősz and Markó 2004), Rupite (Steiner et al. 2005); **Eastern Rhodopi Mts:** Byal Izvor vill. (Arda), Zhelezino vill. (Ivaylovgrad), between Odrintsi vill. and Svirachi vill. (Ivaylovgrad) [Lapeva-Gjonova 2004a (as Tetramorium semilaeve)]; **Northern Black Sea coast**: Varna [Csősz and Markó 2004 (as Tetramorium semilaeve)].

###### Notes:

This species, primarily described as a subspecies of Tetramorium caespitum, was raised to species rank by Röszler (1951), but this name was then forgotten for many years, until Csősz and Markó (2004) redescribed it. The latter authors considered Tetramorium hungaricum as a Ponto-Caspian or Balkan element.

##### Tetramorium moravicum

Kratochvil, 1941

###### Records

(Map 40): **Bulgaria** (Csősz and Schulz 2010); **Sofia Basin**: Sofia (Antonova and Penev 2006); **Plana Mt.:** Kokalyane vill. (Steiner et al. 2005), Peyova buka hut (Pasarel vill.) (Vagalinski and Lapeva-Gjonova in press); **Lozenska Planina Mt.:** near German monastery (Antonova and Penev 2008); **Strandzha Mt.:** Malko Tarnovo (Csősz et al. 2007); **Boboshevo-Simitli Valley**: Blagoevgrad (Csősz et al. 2007); **Western Rhodopi Mts:** Dospat (Lapeva-Gjonova in press (a)).

#### Genus Strongylognathus

Mayr, 1853

##### Strongylognathus kratochvili

Silhavy, 1937

Strongylognathus bulgaricus  Pisarski, 1966 (see Notes below).

###### Records

(Map 41): **Central Predbalkan**: Veliko Tarnovo (Viehmeyer 1922), Preobrazhenski monastery (10 km by Veliko Tarnovo), Dryanovo (Atanassov and Dlusskij 1992); **Eastern Predbalkan**: Veliki Preslav (Atanassov and Dlusskij 1992).

###### Notes:

Viehmeyer (1922) described Strongylognathus huberi rehbinderi var. bulgarica from Bulgaria, but this name is unavailable (quadrinomen). Later Pisarski (1966) proposed the first available name for this form (Strongylognathus rehbinderi bulgaricus) and considered Strongylognathus kratochvili as its junior synonym. Nevertheless, based on the International Code of Zoological Nomenclature (1999) the author of Strongylognathus bulgaricus is Pisarski (1966), thus the name Strongylognathus kratochvili has priority. Atanassov and Dlusskij (1992) recorded this species as Strongylognathus bulgaricus.

###### Conservation Status:

Vulnerable D2 (IUCN).

##### Strongylognathus testaceus

(Schenck, 1852)

###### Records

(Map 41): **Bulgaria** (Agosti and Collingwood 1987a, Atanassov and Dlusskij 1992); **Krupnik-Sandanski-Petrich Valley**: Kozhuh Mt. (Atanassov 1964); **Belasitsa Mt.** (Atanassov 1964, Hubenov et al. 1998); **Eastern** **Rhodopi Mts:** Dedets vill. (Zlatograd) (Lapeva-Gjonova 2004a); **Southern Black Sea coast**: Ahtopol (Atanassov and Vassileva 1976).

### Subfamily Dolichoderinae

#### Genus Dolichoderus

Lund, 1831

##### Dolichoderus quadripunctatus

(Linnaeus, 1771)

###### Records

(Map 42): **Bulgaria** (Agosti and Collingwood 1987a); **Central Predbalkan**: Dermantsi vill. (Lukovit) (Atanassov 1936); **Sofia Basin**: Sofia [Atanassov and Dlusskij 1992, Lapeva-Gjonova and Atanasova 2004 (as Hypoclinea quadripunctata), Antonova 2005, Antonova and Penev 2006, 2008], surroundings of Sofia near Vladaya vill. (Antonova and Penev 2006, 2008); **Plana Mt.:** Pasarel vill. (Vagalinski and Lapeva-Gjonova in press); **Bakadzhik-Burgas district**: Aytos (Forel 1892); **Strandzha Mt.** (Antonova et al. in press): Malko Tarnovo [Atanassov and Dlusskij 1992 (as Hypoclinea quadripunctata)]; **Southwestern Bulgaria** [Atanassov and Dlusskij 1992 (as Hypoclinea quadripunctata)]; **Belasitsa Mt.** (Atanassov 1964, Hubenov et al. 1998); **Krupnik-Sandanski-Petrich Valley**: Petrich plain (Atanassov 1964); **Rila Mt.** (Forel 1892); **Western Rhodopi Mts:** Asenovgrad (Forel 1892); **Eastern Rhodopi Mts:** Madzharovo (Lapeva-Gjonova 2004a); **Southern Black Sea coast**: Veselie vill. (Forel 1892).

#### Genus Liometopum

Mayr, 1861

##### Liometopum microcephalum

(Panzer, 1798)

###### Records

(Map 43): **Bulgaria** (Agosti and Collingwood 1987a); **Western Danubian Plain**: Sadovets vill. (Lukovit) (Atanassov 1934), Lukovit (Del Toro et al. 2009); **Eastern Danubian Plain**: Ludogorie-Dobrudzha district (Atanassov 1957, Atanassov and Dlusskij 1992); **Central Predbalkan**: Dermantsi vill. (Lukovit) (Atanassov 1934, 1936, 1957); **Stara Planina Mts** (Atanassov 1957, Atanassov and Dlusskij 1992); **Central Stara Planina Mts:** Ribaritsa vill. (Atanassov 1936); **Eastern** **Stara Planina Mts:** Sliven (Forel 1892); **Podbalkan Basins**: Rose valley (Kazanlak) (Atanassov 1957); **Sredna gora Mts** (Atanassov 1957, Atanassov and Dlusskij 1992); **Thracian Lowland**: Pazardzhik (Forel 1892), Krichim (Atanassov 1936); **Bakadzhik-Burgas district**: Aytos (Forel 1892); **Strandzha Mt.** (Atanassov and Dlusskij 1992, Antonova et al. in press); **Belasitsa Mt.** (Atanassov 1957, 1964, Atanassov and Dlusskij 1992); **Krupnik-Sandanski-Petrich Valley**: Petrich plain, along Strumeshnitsa river (Atanassov 1964); **Rila Mt.** (Atanassov 1957, Atanassov and Dlusskij 1992): Rila (Forel 1892, Del Toro et al. 2009); **Pirin Mt.** (Atanassov 1957, Atanassov and Dlusskij 1992); **Rhodopi Mts** (Atanassov 1957, Atanassov and Dlusskij 1992); **Western Rhodopi Mts:** Dospat (Lapeva-Gjonova in press (a)); **Eastern Rhodopi Mts:** Pastrook vill. (Ivaylovgrad) (Lapeva-Gjonova 2004a); **Southern Black Sea coast**: Sozopol, Veselie vill. (Forel 1892).

#### Genus Tapinoma

Förster, 1850

##### Tapinoma erraticum

(Latreille, 1798)

###### Records

(Map 44): **Bulgaria** [Emery 1914 (as Tapinoma erraticum nigerrimum), Agosti and Collingwood 1987a, Atanassov and Dlusskij 1992]; **Western Predbalkan**: Rachene river valley (Vassilev 1984); **Western Stara Planina Mts:** Chepan Mt. (Dragoman) (Borisova et al. 2005); **Central Stara Planina Mts:** Vezhen hut (Atanassov 1936); **Eastern** **Stara Planina Mts:** Sliven (Forel 1892); **Sofia Basin**: Sofia (Lapeva-Gjonova and Atanasova 2004, Antonova 2005, Antonova and Penev 2006, 2008), surroundings of Sofia near Vladaya village (Antonova and Penev 2006, 2008); **Vitosha Mt.** (Atanassov 1952); **Plana Mt.:** Plana vill., Tsiganka peak (Pasarel vill.), Vrabnitsa loc. (Dolni okol vill.), Turmachka mahala (Plana vill.), Krasta peak (Dolni okol vill.), Pasarel vill., Alino vill. (Vagalinski and Lapeva-Gjonova in press); **Lozenska Planina Mt.** (Vassilev and Evtimov 1973); **Strandzha Mt.** (Antonova et al. in press); **Dupnitsa Basin**: Dupnitsa (Forel 1892); **Belasitsa Mt.** (Atanassov 1964); **Krupnik-Sandanski-Petrich Valley**: along Strumeshnitsa river, Kozhuh Mt. [Atanassov 1964 and Hubenov et al. 1998 (as Tapinoma erraticum nigerrimum)]; **Rila Mt.:** Rilska river valley (Forel 1892); **Pirin Mt.**, **Rhodopi Mts** (Seifert 1984); **Western Rhodopi Mts:** Chepelare, Rakitovo, Batak, Velingrad, Peshtera (Lapeva-Gjonova in press (a)), Radilovo vill. (Atanassov 1936); **Eastern Rhodopi Mts:** Byal Izvor vill. (Arda), Beli Plast vill. (Kardzhali), Momchilgrad, Madzharovo municipality, between Odrintsi and Svirachi vill. (Ivaylovgrad), Senoklas vill. (Madzharovo) (Lapeva-Gjonova 2004a); **Black Sea coast** (Seifert 1984); **Northern Black Sea coast**: Evksinograd palace (Atanassov 1936); **Southern** **Black Sea coast**: Pomorie, Sozopol (Forel 1892).

##### Tapinoma madeirense

Forel, 1895

Tapinoma ambiguum  Emery, 1925

###### Records

(Map 44): **Bulgaria** (Czechowski et al. 2002); **Plana Mt.:** Plana vill. (Vagalinski and Lapeva-Gjonova in press); **Pirin Mt.**, **Rhodopi Mts**, **Black Sea coast** (Seifert 1984).

###### Notes:

Until recently all authors considered this species to be Tapinoma ambiguum, but Wetterer et al. (2007) synonymised it with Tapinoma madeirense.

#### Genus Bothriomyrmex

Emery, 1869

##### Bothriomyrmex communistus

Santschi, 1919

###### Records

(Map 45): **Strandzha Mt.:** Malko Tarnovo (Atanassov and Dlusskij 1992); **Krupnik-Sandanski-Petrich Valley**: west of Petrich (Atanassov 1964, Atanassov and Dlusskij 1992), Petrich (Hubenov et al. 1998), Sandanski (Atanassov and Dlusskij 1992).

##### Bothriomyrmex gibbus

Soudek, 1925

###### Records

(Map 45): **Western Predbalkan**: Rachene river valley (Vassilev 1984).

###### Notes:

Bothriomyrmex gibbus was not recorded for Bulgaria by Atanassov and Dlusskij (1992), and since 1984 no records of this species have been confirmed. However, based on the known distribution, the presence of this species in Bulgaria is quite probable.

##### Bothriomyrmex menozzii

Emery, 1925

###### Records

(Map 45): **Bulgaria** (Csősz and Markó 2005); **Northern Black Sea coast**: Kavarna (Markó and Csősz 2002).

##### Bothriomyrmex meridionalis

(Roger, 1863)

###### Records

(Map 45): **Krupnik-Sandanski-Petrich Valley**: west of Petrich (Atanassov 1964, Hubenov et al. 1998); **Eastern Rhodopi Mts:** Madzharovo (Lapeva-Gjonova 2004a).

#### Genus Linepithema

Mayr, 1866

##### Linepithema humile

(Mayr, 1868)

###### Records

(Map 46): **Sofia Basin**: Sofia; **Northern Black Sea coast**: Varna; **Southern Black Sea coast**: Burgas [Atanassov and Dlusskij 1992 (all as Iridomyrmex humilis)].

### Subfamily Formicinae

#### Genus Plagiolepis

Mayr, 1861

##### Plagiolepis pygmaea

(Latreille, 1798)

###### Records

(Map 47): **Bulgaria** (Agosti and Collingwood 1987a, Atanassov and Dlusskij 1992); **Western Stara Planina Mts:** Chepan Mt. (Dragoman) (Borisova et al. 2005); **Eastern** **Stara Planina Mts:** Sliven (Forel 1892); **Sofia Basin**: Sofia (Forel 1892, Antonova 2005, Antonova and Penev 2006, 2008), the surroundings of Sofia (Antonova and Penev 2006); **Vitosha Mt.** (Atanassov 1952); **Lozenska Planina Mt.** (Vassilev and Evtimov 1973); **Bakadzhik-Burgas district**: Aytos (Forel 1892); **Strandzha Mt.** (Atanassov 1936, Antonova et al. in press); **Osogovska Planina Mt.:** Hisarlaka (Atanassov 1936); **Dupnitsa Basin**: Dupnitsa (Forel 1892); **Eastern Rhodopi Mts:** Dedets vill. (Zlatograd), Byal Izvor vill. (Ardino), Momchilgrad, Beli Plast vill. (Kardzhali), Malko Popovo vill. (Madzharovo), Madzharovo, Zvezdel vill. (Momchilgrad), Zhelezino vill. (Ivaylovgrad), between Odrintsi vill. and Svirachi vill. (Ivaylovgrad), between Dabovets vill. and Kamilski dol vill. (Ivaylovgrad), Svirachi vill. (Ivaylovgrad) (Lapeva-Gjonova 2004a); **Southern Black Sea coast**: Burgas, Veselie vill. (Forel 1892).

##### Plagiolepis taurica

Santschi, 1920

Plagiolepis vindobonensis  Lomnicki, 1925
Plagiolepis vindobonensis  - all authors before 2000 recorded it under this name.

###### Records

(Map 47): **Bulgaria** (Agosti and Collingwood 1987a, Atanassov and Dlusskij 1992); **Western Predbalkan**: Rachene river valley (Vassilev 1984); **Western Stara Planina Mts:** Chepan (Dragoman) (Borisova et al. 2005); **Sofia Basin**: the surroundings of Sofia (Antonova and Penev 2006); **Plana Mt.:** Bukov dol loc. (Pasarel vill.) (Vagalinski and Lapeva-Gjonova in press); **Lozenska Planina Mt.:** north of Pasarel vill. (Antonova and Penev 2008); **Ograzhden Mt., Belasitsa Mt.** (Atanassov 1964); **Krupnik-Sandanski-Petrich Valley**: Petrich plain, Marino pole vill., Mitino vill. (Atanassov 1964); **Western Rhodopi Mt.:** Peshtera, Batak (Lapeva-Gjonova in press (a)).

#### Genus Lepisiota

Santschi, 1926

##### Lepisiota frauenfeldi

(Mayr, 1855)

Acantholepis frauenfeldi  - all authors recorded it under this name.

###### Records

(Map 48): **Bulgaria** (Agosti and Collingwood 1987a, Atanassov and Dlusskij 1992); **Belasitsa Mt.** (Atanassov 1964); **Krupnik-Sandanski-Petrich Valley**: Kresna (Atanassov 1936); **Eastern Rhodopi Mt.:** Svirachi vill. (Ivaylovgrad) (Lapeva-Gjonova 2004a).

##### Lepisiota splendens

(Karavaiev, 1912)

###### Records:

**Bulgaria** [Agosti and Collingwood 1987a (as Acantholepis splendens)].

###### Notes:

This species was omitted by Atanassov and Dlusskij (1992).

#### Genus Prenolepis

Mayr, 1861

##### Prenolepis nitens

(Mayr, 1853)

###### Records

(Map 49): **Bulgaria** (Agosti and Collingwood 1987a, Atanassov and Dlusskij 1992); **Sofia Basin**: Sofia (Antonova 2005, Antonova and Penev 2006, 2008); **Podbalkan Basins**: Rose valley (Atanassov et al. 1955); **Strandzha Mt.** (Antonova et al. in press): Malko Tarnovo (Atanassov and Vassileva 1976, Hubenov et al. 1998); **Krupnik-Sandanski-Petrich Valley**: the valley of Luda Mara river (Atanassov 1964); **Belasitsa Mt.** (Atanassov 1964); **Western Rhodopi Mts:** Radilovo vill. (Peshtera) (Atanassov 1936), Smolyan (Lapeva-Gjonova in press (a)); **Eastern Rhodopi Mts:** Kokiche vill. (Kardzhali) (Lapeva-Gjonova 2004a).

#### Genus Lasius

Fabricius, 1804

##### Lasius alienus

(Förster, 1850)

###### Records

(Map 50): **Bulgaria** (Agosti and Collingwood 1987a, Atanassov and Dlusskij 1992, Seifert 1992); **Western Predbalkan**: Rachene river valley (Vassilev 1984); **Western Stara Planina Mts:** Lakatnik station (Vratsa district) (Atanassov 1936); **Konyavska Planina Mt.:** Uglyartsi vill. (Lapeva-Gjonova 2004b); **Sofia Basin**: Sofia (Forel 1892, Lapeva-Gjonova and Atanasova 2004, Antonova 2004, 2005, Antonova and Penev 2006, Hlaváč et al. 2007), the surroundings of Sofia near Vladaya vill. (Antonova and Penev 2006, 2008); **Vitosha Mt.** (Atanassov 1936, 1952, Hlaváč et al. 2007): Knyazhevo (Forel 1892); **Plana Mt.:** Pasarel vill. (Hlaváč et al. 2007, Vagalinski and Lapeva-Gjonova in press); Tsiganka peak (Pasarel vill.), Bukov dol loc. (Pasarel vill.), Plana vill., Peyova buka hut (Pasarel vill.), Alino vill. (Vagalinski and Lapeva-Gjonova in press); **Lozenska Planina Mt.** (Vassilev and Evtimov 1973): near German monastery (Antonova and Penev 2008); **Bakadzhik-Burgas district**: Aytos (Forel 1892); **Strandzha Mt.** (Antonova et al. in press); **Krupnik-Sandanski-Petrich Valley**: along Strumeshnitsa river (Atanassov 1964); **Belasitsa Mt.** (Atanassov 1964); **Rila Mt.:** Rila monastery, the valley of Rilska river (Forel 1892); **Western Rhodopi Mts:** Chepintsi vill., Arda vill., Koshnitsa vill. (Hlaváč et al. 2007), Dospat, Smolyan, Rakitovo, Velingrad, Chepelare (Lapeva-Gjonova in press (a)); **Eastern Rhodopi Mts:** Kokiche vill. (Kardzhali), Dedets vill. (Zlatograd), Dyadovtsi vill. (Ardino), Madzharovo, Zvezdel vill. (Momchilgrad), between Dabovets vill. and Kamilski dol vill. (Ivaylovgrad), between Odrintsi vill. and Svirachi vill. (Ivaylovgrad), Svirachi vill. (Ivaylovgrad), Zhelezino vill. (Ivaylovgrad), Meden buk vill. (Ivaylovgrad) (Lapeva-Gjonova 2004a); **Northern Black Sea coast**: Evksinograd palace (Atanassov 1936); **Southern** **Black Sea coast**: Veselie vill. (Forel 1892).

###### Notes:

Seifert (1992) has divided the former “Lasius alienus” into several species, three of which are found in Bulgaria: Lasius alienus, Lasius paralienus and Lasius psammophilus. Consequently, records of Lasius alienus in all papers before 1992 may refer to any of these species.

##### Lasius balcanicus

Seifert, 1988

###### Records

(Map 50): **Sofia Basin**: Sofia (Antonova and Penev 2006, 2008); **Plana Mt.:** Bukov dol loc. (Pasarel vill.), Peyova buka hut (Pasarel vill.) (Vagalinski and Lapeva-Gjonova in press); **Pirin Mt.:** Rozhen (Seifert 1988b); **Western Rhodopi Mts:** Dospat (Lapeva-Gjonova in press (a)); **Northern** **Black Sea coast**: Obzor vill. (Seifert 1988b).

###### Notes:

This species was omitted by Atanassov and Dlusskij (1992).

##### Lasius bicornis

(Förster, 1850)

###### Records

(Map 51): **Bulgaria** (Seifert 1988b, Atanassov and Dlusskij 1992); **Lozenska Planina Mt.** (Vassilev and Evtimov 1973); **Krupnik-Sandanski-Petrich Valley**: around Mitino vill. (Atanassov 1964).

##### Lasius brunneus

(Latreille, 1798)

###### Records

(Map 51): **Bulgaria** (Emery 1914, Agosti and Collingwood 1987a, Atanassov and Dlusskij 1992, Seifert 1992); **Central Predbalkan**: Dermantsi vill. (Lukovit) (Atanassov 1934); **Eastern** **Stara Planina Mts:** Sliven (Forel 1892); **Zemen Gorge** (Lapeva-Gjonova 2004b); **Sofia Basin**: Sofia (Lapeva-Gjonova and Atanasova 2004, Antonova 2005, Antonova and Penev 2006, 2008), the surroundings of Sofia near Vladaya vill. (Antonova and Penev 2006, 2008); **Vitosha Mt.** (Atanassov 1952); **Plana Mt.:** Kokalyane monastery (Kokalyane vill.), Richov well loc. (Dolni Okol vill.), Turmachka neighbourhood (Plana vill.), Bukov dol loc. (Pasarel vill.) (Vagalinski and Lapeva- Gjonova in press); **Lozenska Planina Mt.** (Vassilev and Evtimov 1973): near German monastery (Antonova and Penev 2008); **Bakadzhik-Burgas district**: Aytos (Forel 1892); **Strandzha Mt.** (Antonova et al. in press); **Krupnik-Sandanski-Petrich Valley**: west of Petrich, along Strumeshnitsa river (Atanassov 1964); **Dupnitsa Basin**: Dupnitsa (Forel 1892); **Western Rhodopi Mts:** Peshtera (Lapeva-Gjonova in press (a)); **Eastern** **Rhodopi Mts:** Malko Popovo vill. (Madzharovo) (Lapeva-Gjonova 2004a); **Southern** **Black Sea coast**: Veselie vill. (Forel 1892).

##### Lasius carniolicus

Mayr, 1861

###### Records

(Map 52): **Thracian Lowland**: Svilengrad (Atanassov and Dlusskij 1992); **Krupnik-Sandanski-Petrich Valley**: Petrich (Atanassov and Dlusskij 1992); **Southern** **Black Sea coast**: Tsarevo (Atanassov and Dlusskij 1992).

##### Lasius citrinus

Emery, 1922

Lasius affinis  (Schenck, 1852)

###### Records

(Map 52): **Eastern Danubian Plain**: Tutrakan [Atanassov and Dlusskij 1992 (as Lasius affinis)]; **Central Predbalkan**: Elena [Atanassov and Dlusskij 1992 (as Lasius affinis)]; **Eastern Stara Planina Mts:** Prilep [Seifert 1988b (as Lasius affinis)], 30 km north of Karnobat (Seifert 1990), Karandila loc. [Atanassov and Dlusskij 1992 (as Lasius affinis)]; **Sofia Basin**: Sofia [Antonova 2004, 2005, Lapeva-Gjonova and Atanasova 2004 (as Lasius affinis), Antonova and Penev 2006, 2008], the surroundings of Sofia near Vladaya vill. (Antonova and Penev 2006); **Lozenska Planina Mt.:** near German monastery (Antonova and Penev 2008); **Western Rhodopi Mts:** Asenovgrad, Belovo [Atanassov and Dlusskij 1992 (as Lasius affinis)]; **Northern** **Black Sea coast**: Balchik [Atanassov and Dlusskij 1992 (as Lasius affinis)].

##### Lasius distinguendus

(Emery, 1916)

Lasius hybridus  (Bondroit, 1918)

###### Records

(Map 52): **Bulgaria** (Agosti and Collingwood 1987a, Seifert 1988b, Schlick-Steiner et al. 2002); **Western Predbalkan**: Rachene river valley [Vassilev 1984 (as Lasius hybridus)].

###### Notes:

This species was omitted by Atanassov and Dlusskij (1992).

##### Lasius emarginatus

(Olivier, 1792)

###### Records

(Map 53): **Bulgaria** (Agosti and Collingwood 1987a, Seifert 1992); **Eastern Danubian Plain**: Tutrakan (Atanassov and Dlusskij 1992); **Western Predbalkan**: along Rachene river (Vassilev 1984), Belogradchik (Atanassov and Dlusskij 1992); **Central Predbalkan**: Elena (Atanassov and Dlusskij 1992); **Eastern Stara Planina Mts:** Karandila loc. (Atanassov and Dlusskij 1992); **Lozenska Planina Mt.** (Vassilev and Evtimov 1973); **Strandzha Mt.** (Antonova et al. in press); **Krupnik-Sandanski-Petrich Valley**: west of Petrich, along Strumeshnitsa river (Atanassov 1964); **Belasitsa Mt.** (Atanassov and Dlusskij 1992); **Western Rhodopi Mts:** Peshtera, Batak (Lapeva-Gjonova in press (a)), Asenovgrad (Atanassov and Dlusskij 1992); **Eastern** **Rhodopi Mts:** Madzharovo, Padalo vill. (Krumovgrad) (Lapeva-Gjonova 2004a); **Northern** **Black Sea coast**: Balchik (Atanassov and Dlusskij 1992).

##### Lasius flavus

(Fabricius, 1782)

###### Records

(Map 53): **Bulgaria** (Agosti and Collingwood 1987a, Atanassov and Dlusskij 1992); **Western Stara Planina Mts:** Milanovo vill. (Vratsa) (Atanassov 1934); **Central Stara Planina Mts:** Botev peak (Ray hut), Vezhen hut, under Bratanitsa peak (Atanassov 1936); **Sofia Basin**: Sofia (Atanassov 1934, Lapeva-Gjonova 2004b, Lapeva-Gjonova and Atanasova 2004, Antonova 2005, Antonova and Penev 2006, 2008), the surroundings of Sofia (Antonova and Penev 2006); **Lyulin Mt.** (Atanassov 1934); **Vitosha Mt.** (Atanassov 1936, 1952); **Podbalkan Basin**: Rose valley (Atanassov et al. 1955); **Lozenska Planina Mt.** (Vassilev and Evtimov 1973); **Strandzha Mt.:** Balgari vill. (Atanassov 1934); **Belasitsa Mt.** (Atanassov 1964); **Krupnik-Sandanski-Petrich Valley**: along Strumeshnitsa river (Atanassov 1964); **Rila Mt.:** Borovets (Atanassov 1934); **Western Rhodopi Mts:** Dobrostan vill. (Seifert 1983), Smolyan, Rakitovo (Lapeva-Gjonova in press (a)).

##### Lasius fuliginosus

(Latreille, 1798)

###### Records

(Map 54): **Bulgaria** (Agosti and Collingwood 1987a, Atanassov and Dlusskij 1992); **Western Danubian Plain**: Sadovets vill. (Lukovit) (Atanassov 1934); **Central Predbalkan**: Dermantsi vill. (Lukovit) (Atanassov 1934); **Western Stara Planina Mts:** Chepan (Dragoman) (Borisova et al. 2005); **Eastern Stara Planina Mts:** Byala vill. (Sliven) (Atanassov 1936); **Sofia Basin**: Sofia (Forel 1892, Antonova 2004, 2005, Lapeva-Gjonova and Atanasova 2004, Antonova and Penev 2006, 2008), the surroundings of Sofia near Vladaya vill. (Antonova and Penev 2006, 2008); **Lyulin Mt.** (Atanassov 1936); **Vitosha Mt.** (Atanassov 1952); **Plana Mt.:** Plana vill., Peyova buka hut (Pasarel vill.), Tsiganka peak (Pasarel vill.), Astronomical observatory (between Plana vill. and Dolni Okol vill.) (Vagalinski and Lapeva-Gjonova in press); **Podbalkan Basins**: Rose valley (Atanassov et al. 1955); **Lozenska Planina Mt.** (Vassilev and Evtimov 1973); **Thracian Lowland**: Krichim (Atanassov 1936); **Bakadzhik-Burgas district**: Aytos (Forel 1892); **Osogovska Planina Mt.:** Hisarlaka (Atanassov 1936); **Krupnik-Sandanski-Petrich Valley**: west of Petrich (Atanassov 1964); **Western Rhodopi Mts:** Peshtera (Forel 1892, Atanassov 1934), Dospat, Smolyan, Batak, Velingrad (Lapeva-Gjonova in press (a)); **Eastern Rhodopi Mts:** Zhelezino vill. (Ivaylovgrad) (Lapeva-Gjonova 2004a); **Northern Black Sea coast**: Evksinograd palace (Atanassov 1936).

##### Lasius jensi

Seifert, 1982

###### Records

(Map 54): **Western Rhodopi Mts:** Dobrostan (Seifert 1988b).

###### Notes:

This species was omitted by Atanassov and Dlusskij (1992).

##### Lasius meridionalis

(Bondroit, 1920)

###### Records:

**Bulgaria** (Agosti and Collingwood 1987a, Atanassov and Dlusskij 1992).

##### Lasius mixtus

(Nylander, 1846)

###### Records

(Map 54): **Bulgaria** (Emery 1914, Seifert 1988b); **Central Predbalkan**: Dermantsi vill. (Lukovit) (Atanassov 1936), Lovech (Atanassov and Dlusskij 1992); **Western Stara Planina Mts:**Chepan Mt. (Dragoman) (Borisova et al. 2005); **Central Stara Planina Mts:** Teteven (Atanassov and Dlusskij 1992); **Eastern** **Stara Planina Mts:** Kotel (Atanassov and Dlusskij 1992); **Zemen Gorge**: Zemen station (Atanassov 1936); **Osogovska Planina Mt.:** Kyustendil (Atanassov and Dlusskij 1992); **Boboshevo-Simitli Valley**: Kocherinovo (Atanassov and Dlusskij 1992); **Mesta Valley**: Gotse Delchev district (Atanassov and Dlusskij 1992).

##### Lasius myops

Forel, 1894

###### Records

(Map 55): **Bulgaria** (Agosti and Collingwood 1987a); **Central Predbalkan**: Veliko Tarnovo (Seifert 1983); **Vitosha Mt.** (Atanassov 1952); **Northern Black Sea coast**: Obzor vill. (Seifert 1983).

###### Notes:

Atanassov and Dlusskij (1992) considered this species as a junior synonym of Lasius flavus.

##### Lasius neglectus

Van Loon, Boomsma and Andrasfalvy, 1990

###### Records

(Map 55): **Eastern Danubian Plain**: Senokos vill., Dobrich (Espadaler et al. 2007); **Northern** **Black Sea coast**: Albena [Seifert 1992 (as Lasius turcicus), 2000a], Kranevo, Balchik, Kavarna, Varna municipality (Espadaler et al. 2007).

##### Lasius niger

(Linnaeus, 1758)

Lasius niger var. alienoniger  Forel, 1874: Forel 1892, Atanassov 1936, 1952 (see Notes below)

###### Records

(Map 55): **Bulgaria** (Agosti and Collingwood 1987a, Atanassov and Dlusskij 1992, Seifert 1992); **Western Predbalkan**: Krapets vill. [Atanassov 1936 (as Lasius alieno niger)]; **Central** **Predbalkan**: Dermantsi vill. (Lukovit) [Atanassov 1934, 1936 (as Lasius alieno niger)]; **Western Stara Planina Mts:** Chepan (Dragoman) (Borisova et al. 2005); **Eastern** **Stara Planina Mts:** Sliven (Forel 1892); **Zemen Gorge**: Skakavitsa waterfall (Atanassov 1936); **Vitosha Mt.** [Atanassov 1952 (as Lasius niger L. var. *alieno-niger* Forel), Hlaváč et al. 2007]; **Sofia Basin**: Sofia [Atanassov 1936 (as Lasius alieno niger), Antonova 2004, 2005, Lapeva-Gjonova 2004b, Lapeva-Gjonova and Atanasova 2004, Antonova and Penev 2006, 2008, Hlaváč et al. 2007], the surroundings of Sofia near Vladaya vill. (Antonova and Penev 2006, 2008); **Plana Mt.:** Pasarel vill. (Vagalinski and Lapeva-Gjonova in press); **Podbalkan Basins**: Rose valley (Atanassov et al. 1955); **Lozenska Planina Mt.** (Vassilev and Evtimov 1973): near German monastery (Antonova and Penev 2008); **Belasitsa Mt.** (Atanassov 1964); **Krupnik-Sandanski-Petrich Valley**: around Mitino vill., Petrich plain (Atanassov 1964); **Rila Mt.:** the valley of Rilska river [Forel 1892 (as Lasius niger Rasse *alienus var. alieno-niger*)]; **Western Rhodopi Mts:** Asenovgrad [Forel 1892 (as Lasius niger Rasse *alienus var. alieno-niger*)], Devin, Peshtera, Batak (Lapeva-Gjonova in press (a)); **Southern Black Sea coast**: Burgas, Sozopol [Forel 1892 (as Lasius niger Rasse *alienus var. alieno-niger*)], Veselie vill. (Forel 1892).

###### Notes:

Some of the above mentioned records most probably include also closely related Lasius platythorax. Lasius niger alienoniger Forel, 1874 has been considered by different authors to be a separate species or a junior synonym of Lasius niger, but Seifert (1992) proposed it should be considered as *incertae sedis* in Lasius (for details see Bolton 1995, Bolton et al. 2006).

##### Lasius nitidigaster

Seifert, 1996

Lasius rabaudi : Agosti and Collingwood 1987a, Seifert 1988b, *nec* Bondroit, 1917

###### Records

(Map 56): **Bulgaria** (Agosti and Collingwood 1987a, Seifert 1988b); **Central Stara Planina Mts:** Tryavna (Seifert 1988b); **Plana Mt.:** Plana vill. (Vagalinski and Lapeva-Gjonova in press); **Krupnik-Sandanski-Petrich Valley**: Melnik (Seifert 1988b, 1997a); **Northern Black Sea coast**: Obzor (Seifert 1988b, 1997a); **Southern Black Sea coast**: Nesebar (Seifert 1988b).

##### Lasius paralienus

Seifert, 1992

###### Records

(Map 56): **Bulgaria** (Seifert 1992); **Sofia Basin**: Sofia and the surroundings of Sofia (Antonova and Penev 2006); **Plana Mt.:** Plana vill., Astronomical observatory (between Plana vill. and Dolni Okol vill.), Turmachka neighbourhood (Plana vill.), Kiselichki kamak peak (Alino vill.) (Vagalinski and Lapeva-Gjonova in press); **Lozenska Planina Mt.:** north of Pasarel vill. (Antonova and Penev 2008); **Strandzha Mt.** (Antonova et al. in press); **Northern Black Sea coast**: Kavarna (Csősz and Markó 2005).

See also **Notes** to Lasius alienus, above.

##### Lasius platythorax

Seifert, 1991

###### Records

(Map 56): **Sofia Basin**: Sofia, the surroundings of Sofia near Vladaya vill. (Antonova and Penev 2006, 2008); **Plana Mt.:** Pasarel vill., Bukov dol loc. (Pasarel vill.) (Vagalinski and Lapeva-Gjonova in press); **Lozenska Planina Mt.:** near German monastery (Antonova and Penev 2008); **Western Rhodopi Mts:** Peshtera, Batak (Lapeva-Gjonova in press (a)).

See also **Notes** to Lasius niger, above.

##### Lasius psammophilus

Seifert, 1992

###### Records

(Map 57): **Sofia Basin**: Sofia (Antonova and Penev 2006, 2008); **Plana Mt.:** Plana vill., Pasarel vill. (Vagalinski and Lapeva-Gjonova in press).

See also **Notes** to Lasius alienus, above.

##### Lasius umbratus

(Nylander, 1846)

###### Records

(Map 57): **Bulgaria** (Atanassov and Dlusskij 1992); **Sofia Basin**: Sofia (Forel 1892, Lapeva-Gjonova and Atanasova 2004); **Ograzhden Mt.** (Atanassov 1964); **Western Rhodopi Mts:** Dospat (Lapeva-Gjonova in press (a)); **Eastern Rhodopi Mts:** Dedets vill. (Zlatograd) (Lapeva-Gjonova 2004a).

#### Genus Camponotus

Mayr, 1861

##### Camponotus aegaeus

Emery, 1915

###### Records

(Map 58): **Krupnik-Sandanski-Petrich Valley**: Kamenitsa vill., Mikrevo vill.; **Pirin Mt.:** Kalimantsi vill. (Lapeva-Gjonova in press (a)).

##### Camponotus aethiops

(Latreille, 1798)

Camponotus marginatus  Latreille, 1798 (Forel 1892)
Camponotus aethiops concavus  Dalla Torre, 1893
Camponotus aethiops sylvaticoides  Dalla Torre, 1893

###### Records

(Map 58): **Bulgaria** (Emery 1914, Agosti and Collingwood 1987a, Atanassov and Dlusskij 1992); **Western Predbalkan**: Rachene river valley (Vassilev 1984); **Western Stara Planina Mts:** Ledenika cave (Vratsa) [Atanassov 1934 (as Camponotus maculatus aethiops var. concava)], Chepan (Dragoman) (Borisova et al. 2005); **Eastern** **Stara Planina Mts:** Sliven [Forel 1892 (as Camponotus maculatus Rasse *aethiops* Latr.)]; **Sofia Basin**: Sofia [Forel 1892 (as Camponotus marginatus), Antonova and Penev 2006], the surroundings of Sofia (Antonova and Penev 2006); **Vitosha Mt.** (Atanassov 1936, 1952); **Plana Mt.:** Alino vill., Turmachka neighbourhood, Peyova buka hut (Pasarel vill.) (Vagalinski and Lapeva-Gjonova in press); **Podbalkan Basin**: Rose valley (Atanassov et al. 1955); **Ihtimanska Sredna Gora**: Benkovski peak [Atanassov 1934 (as Camponotus maculatus aethiops)]; **Lozenska Planina Mt.** (Vassilev and Evtimov 1973): German monastery [Atanassov 1934 (as Camponotus maculatus aethiops var. concava)], north of Pasarel vill. (Antonova and Penev 2008); **Thracian Lowland**: Pazardzhik [Forel 1892 (as Camponotus marginatus)]; **Bakadzhik-Burgas district**: Aytos [Forel 1892 (as Camponotus marginatus, Camponotus maculatus Rasse *aethiops* Latr. var. *concavus* Forel and Camponotus maculatus Rasse *aethiops* Latr.)]; **Strandzha Mt.** (Antonova et al. in press); **Dupnitsa Basin**: Dupnitsa [Forel 1892 (as Camponotus maculatus Rasse *aethiops* Latr.)]; **Krupnik-Sandanski-Petrich Valley**: along Strumeshnitsa river, around Marino pole vill. (Atanassov 1964); **Ograzhden Mt.** (Atanassov 1964); **Belasitsa Mt.** (Atanassov 1964); **Slavianka Mt.** (Atanassov 1936); **Western Rhodopi Mts** [Atanassov 1934 (as Camponotus maculatus aethiops)]; **Western Rhodopi Mts:** Asenovgrad [Forel 1892 (as Camponotus maculatus Rasse *aethiops* Latr. var. *sylvaticoides*)], Dospat (Lapeva-Gjonova in press (a)); **Eastern Rhodopi Mts:** Kokiche vill. (Kardzhali), Byal Izvor vill. (Ardino), Momchilgrad, Madzharovo, Malko Popovo vill. (Madzharovo), Senoklas vill. (Madzharovo), Zhelezino vill. (Ivaylovgrad),Malki Voden vill. (Madzharovo), between Dabovets vill. and Kamilski dol vill. (Ivaylovgrad), between Odrintsi vill. and Svirachi vill. (Ivaylovgrad) (Lapeva-Gjonova 2004a); **Southern Black Sea coast**: Veselie vill. [Forel 1892 (as Camponotus marginatus and Camponotus maculatus Rasse *aethiops* Latr *.*)], Sozopol [Forel 1892 (as Camponotus maculatus Rasse *aethiops* Latr.)].

##### Camponotus dalmaticus

(Nylander, 1849)

Camponotus lateralis var. dalmaticus  Nyl.: Forel 1892

###### Records

(Map 58): **Bulgaria** (Agosti and Collingwood 1987a); **Eastern Stara Planina Mts:** Karandila loc. (Atanassov and Dlusskij 1992); **Ihtimanska Sredna Gora Mts:** Benkovski peak (Atanassov 1934, Atanassov and Dlusskij 1992); **Thracian Lowland**: Pazardzhik (Forel 1892, Atanassov and Dlusskij 1992), Svilengrad (Atanassov and Dlusskij 1992); **Belasitsa Mt.** (Atanassov 1964, Atanassov and Dlusskij 1992); **Krupnik-Sandanski-Petrich Valley**: Petrich plain (Atanassov 1964), Kresna gorge, Petrich (Atanassov and Dlusskij 1992); **Rila Mt.** (Forel 1892); **Slavianka Mt.:** Petrovo vill. (Atanassov and Dlusskij 1992); **Western Rhodopi Mts:** Podkova vill., Asenovgrad (Atanassov and Dlusskij 1992); **Eastern Rhodopi Mts:** Momchilgrad (Atanassov and Dlusskij 1992); Madzharovo (Lapeva-Gjonova 2004a); **Southern Black Sea coast**: Tsarevo (Atanassov and Dlusskij 1992).

##### Camponotus fallax

(Nylander, 1856)

Camponotus caryae : Vassilev and Evtimov 1973, *nec* Fitch, 1855

###### Records

(Map 59): **Bulgaria** (Agosti and Collingwood 1987a, Atanassov and Dlusskij 1992); **Sofia Basin**: Sofia (Atanassov 1934, Lapeva-Gjonova and Atanasova 2004, Antonova 2005, Antonova and Penev 2006, 2008), the surroundings of Sofia near Vladaya vill. (Antonova and Penev 2006, 2008); **Plana Mt.:** Bukov dol loc. (Pasarel vill.) (Vagalinski and Lapeva-Gjonova in press); **Lozenska Planina Mt.** [Vassilev and Evtimov 1973 (as Camponotus caryae)]: German monastery (Atanassov 1934); **Krupnik-Sandanski-Petrich Valley**: Parvomay vill. (Atanassov 1964).

##### Camponotus gestroi

Emery, 1878

###### Records

(Map 59): **Krupnik-Sandanski-Petrich Valley**: Kamenitsa vill. (Lapeva-Gjonova in press (a)).

##### Camponotus herculeanus

(Linnaeus, 1758)

###### Records

(Map 59): **Bulgaria** (Agosti and Collingwood 1987a, Atanassov and Dlusskij 1992); **Stara Planina Mts** (Seifert 1989); **Western Stara Planina Mts:** Vilya-glava mine (Atanassov 1934); **Sofia Basin**: Sofia (Atanassov 1934); **Vitosha Mt.** (Atanassov 1936, 1952); **Plana Mt.:** above Dyavolski most bridge (Kokalyane vill.), Bukov dol loc. (Pasarel vill.), Peyova buka hut (Pasarel vill.) (Vagalinski and Lapeva-Gjonova in press); **Ihtimanska Sredna Gora Mts:** Benkovski peak (Atanassov 1934); **Strandzha Mt.:** Papia peak (Atanassov 1934); **Belasitsa Mt.** (Atanassov 1964); **Krupnik-Sandanski-Petrich Valley**: along Luda Mara river (Atanassov 1964); **Rila Mt.** (Seifert 1989): Elenin peak, Rilska river valley (Forel 1892), Borovets (Atanassov 1934, 1936), Kostenets (Atanassov 1934); **Pirin Mt.** (Seifert 1989): Bansko-Banderitsa (Atanassov 1934); **Rhodopi Mts** (Seifert 1989); **Western Rhodopi Mts:** Karlak peak (Atanasov 1934), Perelik peak (Atanassov 1936), Rakitovo, Batak (Lapeva-Gjonova in press (a)); **Southern Black Sea coast**: Maslen nos (Atanassov 1934).

###### Notes:

Camponotus herculeanus is aboreo-montane species and its record from “Maslen nos” seems doubtful.

##### Camponotusionius

Emery, 1920

###### Records

(Map 60): **Bulgaria** (Agosti and Collingwood 1987a); **Krupnik-Sandanski-Petrich Valley**: Kozhuh Mt. (Atanassov 1964).

###### Notes:

This species was omitted by Atanassov and Dlusskij (1992).

##### Camponotus lateralis

(Olivier, 1792)

###### Records

(Map 60): **Bulgaria** (Agosti and Collingwood 1987a, Atanassov and Dlusskij 1992); **Central Predbalkan**: Dermantsi vill. (Lukovit) (Atanassov 1936); **Eastern** **Stara Planina Mts:** Sliven (Forel 1892); **Sofia Basin**: Sofia (Atanassov 1934); **Vitosha Mt.:** Boyana waterfall (Atanassov 1934); **Lozenska Planina Mt.** (Vassilev and Evtimov 1973); **Strandzha Mt.** (Atanassov 1934, Antonova et al. in press); **Belasitsa Mt.** (Atanassov 1964, Hubenov et al. 1998); **Slavianka Mt.** (Atanassov 1936); **Western Rhodopi Mts:** Asenovgrad (Forel 1892); **Eastern Rhodopi Mts:** Kokiche vill. (Kardzhali), Zvezdel vill. (Momchilgrad), between Odrintsi vill. and Svirachi vill. (Ivaylovgrad), between Dabovets vill. and Kamilski dol vill. (Ivaylovgrad) (Lapeva-Gjonova 2004a); **Southern Black Sea coast**: Sozopol (Forel 1892).

##### Camponotus ligniperdus

(Latreille, 1802)

Camponotus herculeanus herculeanoligniperda  Forel, 1874

###### Records

(Map 60): **Bulgaria** (Agosti and Collingwood 1987a, Atanassov and Dlusskij 1992, Seifert 2008); **Central Predbalkan**: Dermantsi vill. (Lukovit) [Atanassov 1934 (as Camponotus herculeanus var. herculeano-ligniperda)]; **Stara Planina Mts** (Seifert 1989); **Western Stara Planina Mts:** Iskrets vill. (Atanassov 1934); **Zemen Gorge**: Zemen (Atanassov 1934); **Sofia Basin**: Sofia, Eleshnitsa vill. (Atanassov 1934), surroundings of Sofia near Vladaya vill. (Antonova and Penev 2006, 2008); **Vitosha Mt.** (Atanassov 1934, 1952): Dragalevtsi (Atanassov 1934), Vladaya vill. (Atanasov 1936); **Plana Mt.:** Astronomical observatory (between Plana vill. and Dolni Okol vill.), Tsiganka peak (Pasarel vill.), Pasarel vill., Bukov dol loc. (Pasarel vill.), Peyova buka hut (Pasarel vill.) (Vagalinski and Lapeva-Gjonova in press); **Podbalkan Basins**: Karlovo gorge (Atanassov 1934); **Ihtimanska Sredna Gora Mts:** Benkovski peak (Atanassov 1934); **Lozenska Planina Mt.** (Vassilev and Evtimov 1973): German monastery [Atanassov 1934 (as Camponotus herculeanus var. herculeano-ligniperda), Antonova and Penev 2008]; **Belasitsa Mt.** (Atanassov 1964); **Rila Mt.** (Seifert 1989): Rila monastery (Forel 1892), Borovets [Atanassov 1934 (as Camponotus herculeanus var. herculeanoligniperda)], Kostenets (Atanassov 1936); **Pirin Mt.** (Seifert 1989): Bansko-Banderitsa, Damyanitsa vill. (Atanassov 1934); **Rhodopi Mts** (Seifert 1989); **Western Rhodopi Mts:** Batak (Atanassov 1934), Dospat, Smolyan (Lapeva-Gjonova in press (a)); **Eastern Rhodopi Mt.:** Dyadovtsi vill. (Ardino) (Lapeva-Gjonova 2004a); **Northern Black Sea coast**: Evksinograd (Atanassov 1934).

##### Camponotus piceus

(Leach, 1825)

Camponotus atricolor  (Nylander, 1849)
Camponotus merula  (Losana, 1834)
Camponotus foveolatus  (Mayr, 1853)
Camponotus lateralis  var. *rectus* Forel, 1892

###### Records

(Map 61): **Bulgaria** [Emery 1914 (as Camponotus lateralis var. atricolor), Agosti and Collingwood 1987a (as Camponotus atricolor)]; **Western Predbalkan**: Rachene river valley [Vassilev 1984 (as Camponotus merula)], Belogradchik (Atanassov and Dlusskij 1992); **Eastern** **Stara Planina Mts:** Sliven [Forel 1892 (as Camponotus lateralis var. atricolor)]; **Sofia Basin**: Sofia [Forel 1892, Atanassov 1934 (as Camponotus lateralis var. atricolor), Antonova 2005, Antonova and Penev 2006, 2008]; surroundings of Sofia near Vladaya vill. (Antonova and Penev 2006, 2008); **Vitosha Mt.** [Atanassov 1952 (as Camponotus lateralis var. atricolor)]: Knyazhevo [Forel 1892 (as Camponotus lateralis var. foveolatus)]; **Plana Mt.:** Alino vill., Bukov dol loc. (Pasarel vill.) (Vagalinski and Lapeva-Gjonova in press); **Lozenska Planina Mt.:** north of Pasarel vill. (Antonova and Penev 2008); **Thracian Lowland**: Pazardzhik [Forel 1892 (as Camponotus lateralis var. foveolatus and Camponotus lateralis var. dalmaticus)]; **Bakadzhik-Burgas district**: Aytos [Forel 1892 (as Camponotus lateralis var. foveolatus)]; **Strandzha Mt.** (Antonova et al. in press): Malko Tarnovo (Atanassov and Vassileva 1976, Hubenov et al. 1998); **Dupnitsa Basin**: Dupnitsa [Forel 1892 (as Camponotus lateralis var. atricolor)]; **Belasitsa Mt.** (Atanassov 1964, Hubenov et al. 1998); **Rila Mt.:** Rilska river valley [Forel 1892 (as Camponotus lateralis var. atricolor, Camponotus lateralis var. foveolatus and Camponotus lateralis var. dalmaticus)]; **Krupnik-Sandanski-Petrich Valley**: Petrich plain (Atanassov 1964); **Eastern Rhodopi Mts:** Zvezdel vill. (Momchilgrad), Malki Voden vill. (Madzharovo), Padalo vill. (Krumovgrad) (Lapeva-Gjonova 2004a); **Southern Black Sea coast**: Pomorie [Forel 1892 (as Camponotus lateralis var. foveolatus and Camponotus lateralis var. rectus)], Sozopol [Forel 1892 (as Camponotus lateralis var. atricolor, Camponotus lateralis var. foveolatus and Camponotus lateralis var. rectus)].

###### Notes:

For most of the last two decades Camponotus atricolor has been considered as a junior synonym of Camponotus piceus. Seifert (1996) noted Camponotus atricolor as separate species, while Radchenko (1997b) considerd it as junior synonym of Camponotus piceus. Markó et al. (2006) revived this name from synonymy, but stressed that confirmation of the status of this species needs further investigation.

##### Camponotus samius

Forel, 1889

Camponotus spagnolinii  Emery, 1920

###### Records

(Map 61): **Bulgaria** (Agosti and Collingwood 1987a); **Podbalkan Basins**: Sotirya vill. (Sliven) [Atanassov and Vassileva 1976 (as Camponotus spagnolinii)]; **Strandzha Mt.** (Antonova et al. in press); **Krupnik-Sandanski-Petrich Valley**: Kozhuh Mt. (Atanassov 1964); **Western Rhodopi Mts:** Vishteritsa [Atanassov and Vassileva 1976 (as Camponotus spagnolinii)], Asenovgrad [Atanassov and Vassileva 1976 (as Camponotus spagnolinii), Hubenov et al. 1998); **Eastern Rhodopi Mts:** Madzharovo, Malko Popovo vill. (Madzharovo), Zhelezino vill. (Ivaylovgrad),between Odrintsi vill. and Svirachi vill. (Ivaylovgrad), between Dabovets vill. and Kamilski dol vill. (Ivaylovgrad), Meden buk vill. (Ivaylovgrad) (Lapeva-Gjonova 2004a); **Northern Black Sea coast**: Obzor vill. [Atanassov and Vassileva 1976 (as Camponotus spagnolinii)]; **Southern Bulgaria** (Atanassov and Dlusskij 1992).

##### Camponotus sylvaticus

(Olivier, 1792)

###### Records

(Map 61): **Lozenska Planina Mt.** (Vassilev and Evtimov 1973).

###### Notes:

This species has not been recorded from Bulgaria since 1973 and its presence in this territory needs confirmation.

##### Camponotus truncatus

(Spinola, 1808)

###### Records

(Map 62): **Bulgaria** (Agosti and Collingwood 1987a, Borowiec 2007); **Stara Planina Mts** (Atanassov and Dlusskij 1992); **Sofia Basin**: Sofia (Lapeva-Gjonova and Atanasova 2004, Antonova 2005, Antonova and Penev 2006, 2008); **Krupnik-Sandanski-Petrich Valley**: west of Petrich, around Parvomay vill. (Atanassov 1964); **Western Rhodopi Mts:** Asenovgrad [Forel 1892 (as Colobopsis truncata)]; **Eastern Rhodopi Mts:** Madzharovo (Lapeva-Gjonova 2004a); **Southwestern Bulgaria** (Atanassov and Dlusskij 1992).

##### Camponotus vagus

(Scopoli, 1763)

###### Records

(Map 62): **Bulgaria** (Agosti and Collingwood 1987a, Atanassov and Dlusskij 1992); **Central Predbalkan**: Dermantsi vill. (Lukovit) (Atanassov 1934); **Western Stara Planina Mts:** Ogoya vill. (Atanassov 1934), Ledenika cave (Vratsa Balkan) (Atanassov 1936); **Central Stara Planina Mts:** Kostinya river valley (Teteven) (Atanassov 1936); **Zemen Gorge**: Zemen (Atanassov 1934); **Sofia Basin**: Sofia (Atanassov 1936, Lapeva-Gjonova and Atanasova 2004, Antonova 2005, Antonova and Penev 2006, 2008), the surroundings of Sofia (Antonova and Penev 2006); **Lyulin Mt.** (Atanassov 1934); **Vitosha Mt.** (Atanassov 1936, 1952); **Plana Mt.:** Bukov dol loc. (Pasarel vill.), Pasarel vill. (Vagalinski and Lapeva-Gjonova in press); **Podbalkan Basins**: Rose valley (Atanassov et al. 1955); **Ihtimanska Sredna Gora Mts:** Benkovski peak (Atanassov 1934); **Lozenska Planina Mt.** (Vassilev and Evtimov 1973); **Thracian Lowland**: Krichim (Atanassov 1936); **Strandzha Mt.:** Papia peak (Atanassov 1934), Malko Tarnovo (Atanassov 1936); **Osogovska Planina Mt.:** Hisarlaka (Kyustendil) (Atanassov 1936); **Belasitsa Mt.** (Atanassov 1964); **Krupnik-Sandanski-Petrich Valley**: along Strumeshnitsa river, around Parvomay vill. (Atanassov 1964); **Slavianka Mt.** (Atanassov 1936); **Western Rhodopi Mts:** Dospat, Batak (Lapeva-Gjonova in press (a)); **Eastern Rhodopi Mts:** Madzharovo (Lapeva-Gjonova 2004a); **Northern Black Sea coast**: Evksinograd palace (Atanassov 1936); **Southern** **Black Sea coast**: Veselie vill. (Forel 1892).

#### Genus Formica

Linnaeus, 1758

##### Formica aquilonia

Yarrow, 1955

###### Records

(Map 63): **Western Predbalkan**: Belogradchik (Atanassov and Dlusskij 1992); **Rila Mt.:** Zavrachitsa hut (Wesselinoff 1973).

###### Conservation Status:

Lower Risk/near threatened (IUCN).

##### Formica cinerea

Mayr, 1853

Formica balcanina  Petrov and Collingwood, 1993
Formica fuscocinerea : Atanassov 1936, 1952, 1964, Atanassov et al. 1955, *nec* Forel, 1874

###### Records

(Map 63): **Bulgaria** (Emery 1914, Atanassov and Dlusskij 1992, Petrov and Collingwood 1993); **Western Predbalkan**: Rachene river valley (Vassilev 1984); **Western Stara planina Mts:** Chepan Mt. (Dragoman) (Borisova et al. 2005); **Sofia Basin**: Sofia [Atanassov 1936 (as Formica cinerea var. fusco-cinerea), Antonova 2004 (as Formica balcanina), 2005, Lapeva-Gjonova and Atanasova 2004 (as Formica balcanina), Antonova and Penev 2006, 2008], surroundings of Sofia near Vladaya vill. (Antonova and Penev 2006, 2008); **Vitosha Mt.** [Atanassov 1936 (as Formica cinerea var. fusco-cinerea), 1952]; **Plana Mt.:** Plana vill., Pasarel vill., Turmachka neighbourhood (Plana vill.), Dolni Okol vill., Astronomical observatory (between Plana vill. and Dolni Okol vill.), Rechyov kamak peak (Plana vill.) (Vagalinski and Lapeva-Gjonova in press); **Podbalkan Basins**: Rose valley (Atanassov et al. 1955); **Lozenska Planina Mt.** (Vassilev and Evtimov 1973), north of Passarel vill. (Antonova and Penev 2008); **Thracian Lowland**: Pazardzhik (Forel 1892); **Krupnik-Sandanski-Petrich Valley**: Petrich, Mitino vill., Parvomay, along Strumeshnitsa river [Atanassov 1964 (as Formica cinerea var. fusco-cinerea)]; **Rila Mt.:** Rila monastery (Forel 1892); **Pirin Mt.:** Melnik, Rozhen, Karlanovo vill. (Seifert 2002b); **Western Rhodopi Mts:** Sveta Petka vill., Velingrad, Bachkovo vill. (Seifert 2002b), Chervenata stena reserve (Hlaváč et al. 2007), Dospat, Devin, Velingrad, Laki, Chepintsi vill., Batak (Lapeva-Gjonova in press (a)); **Eastern Rhodopi Mts:** Byal Izvor vill. (Ardino), Senoklas vill. (Madzharovo), Dyadovtsi vill. (Ardino) [Lapeva-Gjonova 2004a (as Formica balcanina)]; **Northern Black Sea coast**: Zlatni pyasatsi (Seifert 2002b).

##### Formica clara

Forel, 1886

Formica lusatica  Seifert, 1997

###### Records

(Map 63): **Bulgaria** [Seifert 1997b, 2008 (as Formica lusatica)]; **Plana Mt.:** Plana vill., near Peyova buka hut (Pasarel vill.) (Vagalinski and Lapeva-Gjonova in press); **Northern Black Sea coast**: Obzor vill. (Seifert and Schultz 2009); **Southern Black Sea coast**: Nesebar (Barrett 1970).

###### Notes:

This species was not recorded for Bulgaria by Atanassov and Dlusskij (1992), but very recently its occurrence in this country was confirmed by Seifert and Schultz (2009).

##### Formica cunicularia

Latreille, 1798

Formica fusca var. fuscorufibarbis  Forel, 1874

###### Records

(Map 64): **Bulgaria** (Agosti and Collingwood 1987a, Atanassov and Dlusskij 1992); **Western Predbalkan**: Rachene river valley (Vassilev 1984); **Western Stara Planina Mts:** Chepan Mt. (Dragoman) (Borisova et al. 2005); **Sofia Basin**: Sofia (Lapeva-Gjonova and Atanasova 2004, Antonova 2005, Antonova and Penev 2006, 2008), surroundings of Sofia (Barrett 1970, Antonova and Penev 2006), near Vladaya vill. (Antonova and Penev 2008); **Plana Mt.:** Plana vill., Peyova buka hut (Pasarel vill.), Astronomical observatory (between Plana vill. and Dolni Okol vill.), Alino vill. (Vagalinski and Lapeva-Gjonova in press); **Lozenska Planina Mt.:** north of Pasarel vill. (Antonova and Penev 2008); **Bakadzhik-Burgas district**: Aytos [Forel 1892 (as Formica fusca gagates var. fusco-rufibarbis)]; **Strandzha Mt.** (Antonova et al. in press); **Eastern Rhodopi Mts:** Dedets vill. (Zlatograd), Madzharovo, Zhelezino vill. (Ivaylovgrad), Meden buk vill. (Ivaylovgrad) (Lapeva-Gjonova 2004a); **Southern Black Sea coast**: Burgas [Forel 1892 (as Formica fusca gagates var. fusco-rufibarbis)].

##### Formica exsecta

Nylander, 1846

###### Records

(Map 64): **Bulgaria** (Emery 1914, Agosti and Collingwood 1987a, Seifert 2000c); **Predbalkan** [Wesselinoff 1973 (as Coptoformica exsecta)]; **Central Predbalkan**: Dermantsi vill. (Lukovit) (Atanassov 1934); **Stara Planina Mts** [Bobev 1972, Wesselinoff 1973 (as Coptoformica exsecta), Vatov and Bobev 1976, Atanassov and Dlusskij 1992]; **Western Stara Planina Mts:** Gerana mine (Vratsa district), Petrohan-Kom (Atanassov 1936); **Central Stara Planina Mts:** Vezhen hut, Zhaltets peak, Bratanitsa peak (Atanassov 1936), Dobrila peak [Wesselinoff 1973 (as Coptoformica exsecta)], Tsarichina reserve (under Vezhen peak), Dermenka hut (Atanassov and Dlusskij 1992); **Viskyar Mt.**, **Lyulin Mt.**, **Verila Mt.** [Wesselinoff 1973 (as Coptoformica exsecta)]; **Vitosha Mt.** [Atanassov 1934, 1936, 1952, Wesselinoff 1967, 1973 (as Coptoformica exsecta), Atanassov and Dlusskij 1992]: Momina skala loc. (Atanassov 1934); **Plana Mt.** [Wesselinoff 1967, 1973 (as Coptoformica exsecta)]; **Sredna Gora Mts** (Bobev 1972, Vatov and Bobev 1976); **Osogovo-Belasitsa group** (Vatov and Bobev 1976); **Osogovska Planina Mt.**, **Slavianka Mt.**, **Belasitsa Mt.** (Atanassov and Dlusskij 1992); **Rila-Pirin group** (Bobev 1972, Vatov and Bobev 1976); **Rila Mt.** [Wesselinoff 1973 (as Coptoformica exsecta), 1979, Vesselinov 1981, Atanassov and Dlusskij 1992]: Elenin peak (Forel 1892), Parangalitsa reserve [Wesselinoff 1968 and 1973 (as Coptoformica exsecta)], Ibar reserve (Atanassov 1983); **Pirin Mt.** [Wesselinoff 1973 (as Coptoformica exsecta), 1979, Vesselinov 1981, Atanassov and Dlusskij 1992]; **Rhodopi Mts** [Wesselinoff 1973 (as Coptoformica exsecta), 1979, Vesselinov 1981, Atanassov 1983, Atanassov and Dlusskij 1992]; **Western Rhodopi Mts:** Devin (Lapeva-Gjonova in press (a)); **Eastern Rhodopi Mts** (Bobev 1972, Vatov and Bobev 1976).

##### Formica fusca

Linnaeus, 1758

###### Records

(Map 65): **Bulgaria** (Agosti and Collingwood 1987a, Atanassov and Dlusskij 1992); **Western Danubian Plain**: Vidin (Gateva 1975); **Central Predbalkan**: Dermantsi vill. (Lukovit) (Atanassov 1934); **Sofia Basin**: Sofia, Vrania palace (Atanassov 1934), the surroundings of Sofia (Atanassov 1934, Antonova 2005, Antonova and Penev 2006); **Vitosha Mt.** (Atanassov 1952); **Plana Mt.:** Plana vill., Kokalyane monastery (Kokalyane vill.), Bukov dol loc. (Pasarel vill.) (Vagalinski and Lapeva-Gjonova in press); **Lozenska Planina Mt.** (Vassilev and Evtimov 1973): German monastery (Atanassov 1934); **Thracian Lowland**: Krichim (Atanassov 1934); **Rila Mt.:** Elenin peak (Forel 1892); **Slavianka Mt.:** Alibotush reserve (Antonova 2009); **Western Rhodopi Mts:** Smolyan (Gateva 1975, Lapeva-Gjonova in press (a)), Peshtera (Lapeva-Gjonova in press (a)).

##### Formica gagates

Latreille, 1798

Formica fusca r. gagates var. cinereofuscoides  Forel, 1892 (unavailable name)
Formica fusca var. fuscorufibarbis  Forel, 1874: Forel 1892 (as Formica fusca r. gagates var. fuscorufibarbis, unavailable name).
Formica fusca var. cinereorufibarbis  Forel, 1874: Forel 1892 (as Formica fusca r. gagates var. cinereorufibarbis, unavailable name).

###### Records

(Map 65): **Bulgaria** (Agosti and Collingwood 1987a, Atanassov and Dlusskij 1992); **Central Predbalkan**: Dermantsi vill. (Lukovit) (Atanassov 1934); **Western Stara Planina Mts:** Chepan (Dragoman) (Borisova et al. 2005); **Zemen Gorge**: Krastata ornitsa loc. above Skakavitsa station (Atanassov 1934); **Sofia Basin**: Sofia, Knyazhevo [Forel 1892 (as Formica fusca Rasse *gagates* Latr. var. *cinereorufibarbis*)], Sofia and surroundings (Antonova and Penev 2006); **Plana Mt.:** Pasarel vill., Peyova buka hut (Pasarel vill.) (Vagalinski and Lapeva-Gjonova in press); **Lozenska Planina Mt.** (Vassilev and Evtimov 1973): German monastery (Atanassov 1936, Antonova and Penev 2008)]; **Belasitsa Mt.** (Atanassov 1964); **Rila Mt.** (Forel 1892); **Western Rhodopi Mts:** Asenovgrad (Forel 1892), Syutka peak (Atanassov 1936); **Eastern Rhodopi Mts:** Malko Popovo vill. (Madzharovo), Madzharovo, Kokiche vill. (Kardzhali), Meden buk vill. (Ivaylovgrad) (Lapeva-Gjonova 2004a); **Southern Black Sea coast**: Pomorie (Forel 1892).

##### Formica glauca

Ruzsky, 1895

###### Records

(Map 65): **Bulgaria** (Agosti and Collingwood 1987a); **Central Predbalkan**: along Lukovitsko dere river (Atanassov and Vassileva 1976, Hubenov et al. 1998); **Central Stara Planina Mts:** Teteven, along Ribaritsa river (Atanassov and Vassileva 1976, Hubenov et al. 1998); **Thracian Lowland**: Chirpan (Atanassov and Vassileva 1976 and Hubenov et al. 1998 (as Formica cunicularia glauca)]; **Western Rhodopi Mts:** Asenovgrad (Atanassov and Vassileva 1976).

##### Formica lemani

Bondroit, 1917

###### Records

(Map 66): **Bulgaria** (Agosti and Collingwood 1987a, Atanassov and Dlusskij 1992); **Vitosha Mt.** (Atanassov 1936, 1952, Gateva 1975); **Belasitsa Mt.** (Atanassov 1964); **Western Rhodopi Mts:** Smolyan, Rakitovo, Dospat (Lapeva-Gjonova in press (a)).

##### Formica lugubris

Zetterstedt, 1838

###### Records

(Map 66): **Bulgaria** (Agosti and Collingwood 1987a); **Eastern Danubian Plain**: Stozher vill. (Otto et al. 1962), Razgrad, Ruse, Dobrich (Ronketi and Penev 1966), Suvorovo (Keremidchiev et al. 1972); **Predbalkan** (Wesselinoff 1973); **Eastern** **Predbalkan**:Targovishte (Ronketi and Penev 1966); **Stara Planina Mts:** (Bobev 1972, Wesselinoff 1973, Atanassov 1974, Vatov and Bobev 1976); **Western** **Stara Planina Mts:** under Kom peak, Todorini kukli peaks (Atanassov and Dlusskij 1992); **Central Stara Planina Mts:** Boatin reserve (under Tetevenska baba peak), Tsarichina reserve (under Vezhen peak), Dermenka hut (Troyan Balkan) (Atanassov 1983), Vezhen peak (Atanassov and Dlusskij 1992); **Eastern** **Stara Planina Mts:** Tvarditsa pass, Karandila loc., under Chumerna peak, Razboyna vill. (Atanassov and Dlusskij 1992); **Viskyar Mt.**, **Lyulin Mt.**, **Verila Mt.** (Wesselinoff 1973); **Vitosha Mt.** (Otto et al. 1962, Wesselinoff 1967, 1973, Keremidchiev et al. 1972, Atanassov 1974): Cherni vrach (Atanassov 1974), Marchevski ostrets peak, Selimitsa peak, Kupena peak, Samara (Sedloto) peak (Wesselinoff 1967, Atanassov and Dlusskij 1992); **Plana Mt.** (Wesselinoff 1973): Astronomical observatory (between Plana vill. and Dolni Okol vill.) (Vagalinski and Lapeva-Gjonova in press); **Sredna Gora Mts** (Bobev 1972, Vatov and Bobev 1976):Koprivshtitsa (Vesselinov 1981); **Sakar Mt.** (Atanassov and Dlusskij 1992); **Osogovo-Belasitsa group** (Vatov and Bobev 1976); **Belasitsa Mt.**, **Slavianka Mt.** (Atanassov 1974, Atanassov and Dlusskij 1992): Alibotush reserve (Atanassov 1974); **Krupnik-Sandanski-Petrich Valley**: Sandanski (Gateva 1975); **Rila Mt.** (Bobev 1972, Wesselinoff 1973, 1979, Atanassov 1974, Vatov and Bobev 1976, Vesselinov 1981): under Ibar peak, Borovets (Otto et al. 1962, Atanassov and Dlusskij 1992), Parangalitsa reserve (Wesselinoff 1968, 1973), Semkovo (Wesselinoff 1973), Rila monastery (Gateva 1975), Ibar reserve (Atanassov 1983); **Pirin Mt.** (Bobev 1972, Wesselinoff 1973, 1979, Atanassov 1974, Vatov and Bobev 1976, Vesselinov 1981, Atanassov and Dlusskij 1992, Seifert 1996): Bansko (Otto et al. 1962), Kremenski lakes (Wesselinoff 1973), Bayuvi dupki reserve (Atanassov 1983); **Rhodopi Mts** (Bobev 1972, 1973, Wesselinoff 1973, 1979, Atanassov 1974, Gateva 1974, 1975, 1978, Vatov and Bobev 1976, Vesselinov 1981, Seifert 1996); **Western Rhodopi Mts:** Chehlyovo forestry (near Velingrad) (Otto et al. 1962, Keremidchiev et al. 1972, Wesselinoff 1973, 1974, Atanassov and Dlusskij 1992), Hvoyna vill. (Otto et al. 1962, Atanassov and Dlusskij 1992), Yundola, Vishteritsa, Shiroka Laka (Otto et al. 1962), Mantaritsa-Petlite-Kaynatsite reserve (under Syutka peak) (Atanassov 1983), Snezhanka peak (Gateva 1974, Atanassov and Dlusskij 1992), loc. Terekliytsa, above Smolyan lakes (Gateva 1974), Eshekulak, Syutka peak, under Karatepe peak, Perelik peak (Atanassov and Dlusskij 1992); **Eastern Rhodopi Mts:** (Vatov and Bobev 1976); **Northern Black Sea coast**: Varna, Balchik (Ronketi and Penev 1966).

###### Conservation Status:

Lower Risk/near threatened (IUCN).

##### Formica picea

Nylander, 1846

###### Records

(Map 67): **Central** **Stara Planina Mts:** along Kostinya river; **Eastern Stara Planina Mts:** above Obzor vill.; **Rila Mt.:** above Dolna Bania vill., along Bistritsa river; **Western Rhodopi Mts:** Hvoyna vill. [Atanassov and Dlusskij 1992 (as Formica (Raptiformica) transcaucasica)].

###### Notes:

A discussion of the quite complicate taxonomic history of the name Formica picea Nylander lies outside of the remit of the current work; for details see Dlussky (1967), Atanassov and Dlusskij (1992), Bolton (1995), Bolton et al. (2006) and Seifert (2004).

##### Formica polyctena

Förster, 1850

###### Records

(Map 67): **Bulgaria** (Seifert 2008); **Danubian Plain**: *Dachi usoi* loc., *Dalga polyana* loc. (Wesselinoff 1973); **Eastern Stara Planina Mts:** Balgarka peak,Vetrila peak, Zheravna (Wesselinoff 1973); **Golo bardo Mt.:** around Radomir (Atanassov and Dlusskij 1992); **Rila Mt.:** Malyovitsa peak, southwest of Govedartsi vill. (Atanassov and Dlusskij 1992); **Pirin Mt.:** Mocharata loc. (Wesselinoff 1973).

###### Conservation Status:

Lower Risk/near threatened (IUCN).

##### Formica pratensis

Retzius, 1783

Formica nigricans  Bondroit, 1912
Formica cordieri  Bondroit, 1917

###### Records

(Map 68): **Bulgaria** [Emery 1914, Agosti and Collingwood 1987a (as Formica pratensis and Formica nigricans), Atanassov and Dlusskij 1992, Csősz and Markó 2005]; **Danubian Plain** [Bobev 1972 (as Formica nigricans), Wesselinoff 1973 (as Formica nigricans), 1979, Vatov and Bobev 1976, Vesselinov 1981]; **Eastern Danubian Plain**: between Balchik and Dobrich [Otto et al. 1962 (as Formica nigricans)], Shumen (Gateva 1975); **Predbalkan** [Wesselinoff 1973 (as Formica nigricans)]; **Central Predbalkan**: Lovech (Gateva 1975); **Stara Planina Mts** [Bobev 1972 (as Formica nigricans), Wesselinoff 1973 (as Formica nigricans), 1979, Vatov and Bobev 1976, Vesselinov 1981]; **Western Stara Planina Mts:** Zgorigrad vill. (Vratsa), Batulia vill. (Atanassov 1936), Murgash peak [Wesselinoff 1973 (as Formica nigricans)]; **Central Stara Planina Mts:** Boatin reserve (under Tetevenska baba peak) (Atanassov 1983); **Eastern** **Stara Planina Mts** [Bobev 1972 (as Formica nigricans), Vatov and Bobev 1976, Vesselinov 1981, Wesselinoff 1979]; **Verila Mt.** [Wesselinoff 1967, 1973 (as Formica nigricans)]; **Viskyar Mt.** [Wesselinoff 1973 (as Formica nigricans)]; **Zemen Gorge**: Zemen marsh (Atanassov 1936); **Sofia Basin**: Sofia (Forel 1892, Gateva 1975, Antonova 2005, Antonova and Penev 2006, 2008), the surroundings of Sofia (Antonova and Penev 2006); **Lyulin Mt.** (Atanassov 1936, Wesselinoff 1967, 1973 (as Formica nigricans)]; **Vitosha Mt.** [(Atanassov 1936, 1952, Wesselinoff 1967, 1973 (as Formica nigricans)]: Bistritsa vill. (Lapeva-Gjonova 2004b); **Plana Mt.** [Wesselinoff 1967, 1973 (as Formica nigricans)], Tsiganka peak (Pasarel vill.), Bukov dol loc. (Pasarel vill.), Plana vill., Pasarel vill., Manastirishte peak (Plana vill.), Astronomical observatory (between Plana vill. and Dolni Okol vill.), Mechitski kamak peak (Plana vill.) (Vagalinski and Lapeva-Gjonova in press); **Podbalkan Basins**: Rose valley (Atanassov et al. 1955); **Sredna Gora Mts** [Bobev 1972 (as Formica nigricans), 1973, 1979, Vatov and Bobev 1976, Vesselinov 1981, Wesselinoff 1979]; **Surnena Sredna Gora Mts:** Stara Zagora (Gateva 1975); **Lozenska Planina Mt.** [Wesselinoff 1967 (as Formica nigricans), Vassilev and Evtimov 1973): north of Pasarel vill. (Antonova and Penev 2008); **Sakar-Tundzha district**: along Tundzha river [Wesselinoff 1973 (as Formica nigricans)]; **Strandzha Mt.** [Bobev 1972 (as Formica nigricans), Vatov and Bobev 1976, Wesselinoff 1979, Vesselinov 1981]; **Osogovo-Belasitsa group** [Wesselinoff 1973 (as Formicas nigricans), Vatov and Bobev 1976]; **Osogovska Planina Mt.:** Hisarlaka (Atanassov 1936); **Belasitsa Mt.** (Atanassov 1964); **Krupnik-Sandanski-Petrich Valley**: Petrich (Atanassov 1964), Sandanski (Gateva 1975); **Rila Mt.** [Forel 1892, Otto et al. 1962 (as Formica nigricans), Bobev 1972, Wesselinoff 1973 (as Formica nigricans)]: Ibar reserve (Atanassov 1983); **Pirin Mt.** [Otto et al. 1962 (as Formica nigricans), Bobev 1972 (as Formica nigricans), Wesselinoff 1973 (as Formica nigricans), Gateva 1975]; **Mesta Valley**: Yakoruda (Gateva 1975); **Rhodopi Mts** [Otto et al. 1962 (as Formica nigricans), Wesselinoff 1973 (as Formica nigricans), Gateva 1978, Atanassov 1983]; **Western Rhodopi Mts** [Bobev 1972 (as Formica nigricans)]: Batak, Peshtera, Shiroka laka vill. [Bobev 1973 (as Formica nigricans)], Chepelare, Golyam Beglik dam (Gateva 1975), Dospat (Lapeva-Gjonova in press (a)); **Eastern Rhodopi Mts** [Bobev 1972 (as Formica nigricans), Vatov and Bobev 1976]: Senoklas vill. (Madzharovo) (Lapeva-Gjonova 2004a); **Northern Black Sea coast**: Varna [Otto et al. 1962 (as Formica nigricans), Ronketi and Penev 1966 (as Formica cordieri and Formica nigricans), Gateva 1975], Balchik (Ronketi and Penev 1966); **Southern Black Sea coast**: Burgas (Forel 1892).

###### Conservation Status:

Lower Risk/near threatened (IUCN).

##### Formica pressilabris

Nylander, 1846

###### Records

(Map 68): **Bulgaria** (Atanassov and Dlusskij 1992); **Western Stara Planina Mts:** Petrohan-Kom (Atanassov 1936); **Vitosha Mt.** (Atanassov 1952); **Rila Mt.:** Kostenets (Atanassov 1936); **Rhodopi Mts** (Gateva 1975).

##### Formica rufa

Linnaeus, 1761

###### Records

(Map 69): **Bulgaria** (Agosti and Collingwood 1987a); **Danubian Plain** (Bobev 1972, Wesselinoff 1973, 1979, Vatov and Bobev 1976, Vesselinov 1981); **Eastern Danubian Plain**: Razgrad, Ruse, Dobrich (Ronketi and Penev 1966); **Predbalkan** (Wesselinoff 1973); **Central Predbalkan**: Dermantsi vill. (Lukovit) (Atanassov 1934, 1936), Aglen vill. (Lukovit) (Atanassov 1934); **Eastern Predbalkan**: Targovishte (Ronketi and Penev 1966); **Stara Planina Mts** (Bobev 1972, Wesselinoff 1973, 1979, Atanassov 1974, Vatov and Bobev 1976, Vesselinov 1981, Atanassov and Dlusskij 1992); **Western Stara Planina Mt.:** Sokolets peak, Milanovo vill., Zgorigrad vill. (Atanassov 1934); **Central Stara Planina Mts:** Kostinya river valley, Botev peak (Ray hut) (Atanassov 1936), Boatin reserve (under Tetevenska baba peak), Tsarichina reserve (under Vezhen peak), Dermenka hut (Troyan Balkan) (Atanassov 1983); **Eastern Stara Planina Mts** (Bobev 1972, Vatov and Bobev 1976, Vesselinov 1981, Wesselinoff 1979): Byala vill. (Sliven) (Atanassov 1936); **Viskyar Mt.** (Wesselinoff 1973); **Verila Mt.** (Wesselinoff 1973); **Zemen Gorge**: Zemen marsh (Atanassov 1936); **Sofia Basin**: Sofia (Lapeva-Gjonova and Atanasova 2004, Antonova and Penev 2006), near Vladaya village (Antonova and Penev 2008); **Lyulin Mt.** (Atanassov 1934, Wesselinoff 1973); **Vitosha Mt.** (Atanassov 1934, 1936, 1952, 1974, 1979, Wesselinoff 1967, 1973): Knyazhevo (Atanassov 1934), Dragalevtsi (Atanassov 1936); **Plana Mt.:** Plana vill., Tsiganka peak (Pasarel vill.), Alino vill. (Wesselinoff 1967, 1973, Vagalinski and Lapeva-Gjonova in press); **Sredna Gora Mts** (Bobev 1972, Wesselinoff 1973, 1979, Vatov and Bobev 1976, Vesselinov 1981); **Ihtimanska Sredna Gora Mt.:** Benkovski peak (Atanassov 1934); **Lozenska Planina Mt.** (Wesselinoff 1967, Vassilev and Evtimov 1973, Lapeva-Gjonova 2004b); **Sakar-Tundzha district**: along Tundzha river (Wesselinoff 1973); **Strandzha Mt.** (Bobev 1972, Vatov and Bobev 1976, Wesselinoff 1979, Vesselinov 1981); **Osogovo-Belasitsa group** (Wesselinoff 1973, Vatov and Bobev 1976); **Belasitsa Mt.** (Atanassov 1964, 1974, Wesselinoff 1974, Lapeva-Gjonova 2004b); **Krupnik-Sandanski-Petrich Valley**: Luda Mara river valley (Atanassov 1964), Sandanski (Gateva 1975); **Rila-Pirin group** (Bobev 1972, Vatov and Bobev 1976); **Rila Mt.** (Wesselinoff 1973, 1979, Atanassov 1974, Vesselinov 1981, Atanassov and Dlusskij 1992): Rilska river valley (Forel 1892), Borovets (Atanassov 1934), Parangalitsa reserve (Wesselinoff 1968), Govedartsi vill., Borovets, Raduil, Kostenets (Otto et al. 1962), Ibar reserve (Atanassov 1983), Panichishte (Lapeva-Gjonova 2004b); **Pirin Mt.** (Vesselinov 1981, Wesselinoff 1973, 1979):Razlog, Bansko, Dobrinishte, Gotse Delchev (Otto et al. 1962); **Slavianka Mt.** (Atanassov 1974); **Rhodopi Mts** (Wesselinoff 1973,Gateva 1978,Atanassov 1983, Atanassov and Dlusskij 1992); **Western Rhodopi Mts** (Bobev 1972, 1973, Atanassov 1974): Selishte, Vishteritsa, Eleshnitsa (Otto et al. 1962), Yundola (Otto et al. 1962, Bechev and Stoyanova 2004), Chepelare, Golyam Beglik dam, Smolyan (Gateva 1975), Zdravets hut, Byala cherkva resort, Ravnishta hut, Skalni mostove, Varhovrah hut (Bechev and Stoyanova 2004), Rakitovo, Batak, Peshtera (Lapeva-Gjonova in press (a)); **Eastern Rhodopi Mts** (Bobev 1972, Vatov and Bobev 1976); **Northern Black Sea coast**: Varna, Balchik (Ronketi and Penev 1966).

###### Conservation Status:

Lower Risk/near threatened (IUCN); Protected (Bulgarian Biodiversity Act (2002), Annex 2 and 3).

##### Formica rufibarbis

Fabricius, 1793

###### Records

(Map 70): **Bulgaria** (Agosti and Collingwood 1987a, Atanassov and Dlusskij 1992, Seifert 2008); **Eastern** **Stara Planina Mts:** Sliven [Forel 1892 (as Formica fusca Rasse *rufibarbis*)]; **Sofia Basin**: Sofia (Lapeva-Gjonova and Atanasova 2004, Antonova 2005, Antonova and Penev 2006, 2008); surroundings of Sofia near Vladaya vill. (Antonova and Penev 2006, 2008); **Vitosha Mt.** [Atanassov 1952, Wesselinoff 1967 (as Serviformica rufibarbis)]; **Plana Mt.** (Vagalinski and Lapeva-Gjonova in press); **Lozenska Planina Mt.** [Wesselinoff 1967 (as Serviformica rufibarbis), Vassilev and Evtimov 1973], north of Pasarel vill. (Antonova and Penev 2008); **Bakadzhik-Burgas district**: Aytos [Forel 1892 (as Formica fusca Rasse *rufibarbis*)]; **Strandzha Mt.:** Balgari vill. (Atanassov 1936); **Krupnik-Sandanski-Petrich Valley**: Petrich, along Strumeshnitsa river (Atanassov 1964); **Rila Mt.:** Rila monastery (Forel 1892); **Western Rhodopi Mts:** Asenovgrad [Forel 1892 (as Formica fusca Rasse *rufibarbis*)], Dobrostan (Seifert and Schultz 2009), Dospat, Velingrad, Batak (Lapeva-Gjonova in press (a)); **Southern Black Sea coast**: Sozopol, Pomorie [Forel 1892 (as Formica fusca Rasse *rufibarbis*)], Burgas (Forel 1895).

##### Formica sanguinea

Latreille, 1798

###### Records

(Map 70): **Bulgaria** (Agosti and Collingwood 1987a); **Danubian Plain** [Wesselinoff 1973 (as Raptiformica sanguinea), Vatov and Bobev 1976]; **Western Danubian Plain**: Vidin (Gateva 1975); **Predbalkan** (Wesselinoff 1973); **Central Predbalkan**: Lukovit, Dermantsi vill. (Atanassov 1934); **Stara Planina Mts** [Wesselinoff 1973 (as Raptiformica sanguinea)]; **Western Stara Planina Mts** (Vatov and Bobev 1976): Gerana mine (Vratsa) (Atanassov 1934); **Eastern** **Stara Planina Mts** (Vatov and Bobev 1976): Sliven (Forel 1892); **Verila Mt.** [Wesselinoff 1967 and 1973 (as Raptiformica sanguinea)]; **Viskyar Mt.**, **Lyulin Mt.** [Wesselinoff 1973 (as Raptiformica sanguinea)]; **Sofia Basin**: Sofia and surroundings of Sofia, near Vladaya village (Antonova and Penev 2006, 2008); **Vitosha Mt.** [Atanassov 1936, 1952, Wesselinoff 1973 (as Raptiformica sanguinea), Gateva 1975]: Knyazhevo (Forel 1892), Zlatnite mostove loc. (Atanassov 1934); **Plana Mt.** [Wesselinoff 1973 (as Raptiformica sanguinea)]: Plana vill., Bukov dol loc. (Pasarel vill.), Tsiganka peak (Pasarel vill.), Astronomical observatory (between Plana vill. and Dolni Okol vill.), Pasarel vill. (Vagalinski and Lapeva-Gjonova in press); **Strandzha Mt**. (Vatov and Bobev 1976); **Osogovska Planina Mt.:** Hisarlaka (Kyustendil) (Atanassov 1936); **Rila-Pirin group** (Vatov and Bobev 1976); **Rila Mt.** [Wesselinoff 1973 (as Raptiformica sanguinea)]: Elenin peak (Forel 1892, Atanassov and Dlusskij 1992), Rila monastery (Forel 1892, Gateva 1975), Parangalitsa reserve (Wesselinoff 1968); **Rhodopi Mts** [Wesselinoff 1973 (as Raptiformica sanguinea)]; **Western Rhodopi Mts:** Buynovo vill. (Hlaváč et al. 2007), Smolyan (Gateva 1975, Lapeva-Gjonova in press (a)), Dospat, Rakitovo (Lapeva-Gjonova in press (a)); **Eastern** **Rhodopi Mts:** Malki Voden vill. (Madzharovo) (Lapeva-Gjonova 2004a).

##### Formica truncorum

Fabricius, 1804

###### Records

(Map 70): **Bulgaria** (Atanassov and Dlusskij 1992); **Danubian Plain** (Wesselinoff 1973); **Western Predbalkan**: Belogradchik (Atanassov and Vassileva 1976, Hubenov et al. 1998), *Bleshnitsa* (Hubenov et al. 1998); **Western Stara Planina Mts:** Berkovitsa (Atanassov and Vassileva 1976); **Eastern** **Stara Planina Mts:**under Razboyna peak (Atanassov and Vassileva 1976); **Osogovska Planina Mt.:** Hisarlaka (Kyustendil) (Atanassov and Vassileva 1976); **Krupnik-Sandanski-Petrich Valley**: Sandanski (Atanassov and Vassileva 1976, Hubenov et al. 1998); **Sturgach Mt.** (Atanassov and Vassileva 1976); **Western Rhodopi Mts:** Eleshnitsa (Atanassov and Vassileva 1976); **Northern** **Black Sea coast**: Kranevo (Atanassov and Vassileva 1976).

#### Genus Proformica

Ruzsky, 1902

##### Proformica kobachidzei

K. Arnoldi, 1968

###### Records

(Map 71): **Boboshevo-Simitli Valley**: Bistritsa vill. (Blagoevgrad) (Atanassov and Dlusskij 1992).

##### Proformica korbi

Emery, 1909

###### Records

(Map 71): **Boboshevo-Simitli Valle**: Kocherinovo (Dlussky 1969).

###### Notes:

This species was omitted by Atanassov and Dlusskij (1992).

##### Proformica nasuta

(Nylander, 1856)

###### Records

(Map 71): **Eastern** **Stara Planina Mts:** Sliven [Atanassov 1934 (as Formica nasuta)]; **Thracian Lowland**: Krichim [Atanassov 1934 (as Formica nasuta)]; **Western Rhodopi Mts:** Radilovo vill. (Peshtera) [Atanassov 1936 (as Formica nasuta)].

###### Notes:

Atanassov and Dlusskij (1992) noted that specimens determined by Forel (1892) and Atanassov (1964) as Proformica nasuta in fact are Proformica striaticeps (see below). At the same time, they stressed that material, based on which (Atanassov (1934, 1936) recorded Proformica nasuta for Bulgaria, has been lost, and it is impossible to make definitive conclusion on the taxonomic status of those ants. Proformica nasuta is definitely distributed in the Iberian Peninsula and France, but it is also recorded from Romania and Greece (see Markó et al. 2006, Radchenko 2007). As we cannot exclude its occurrence in Bulgaria, we include this species in the current list; on the other hand, this data needs confirmation.

##### Proformica pilosiscapus

Dlussky, 1969

###### Records

(Map 72): **Krupnik-Sandanski-Petrich Valley**: Sandanski district (Atanassov and Dlusskij 1992); **Mesta Valley**: Dolno Dryanovo vill. (Atanassov and Dlusskij 1992, Hubenov et al. 1998).

##### Proformica striaticeps

(Forel, 1911)

###### Records

(Map 72): **Western Stara Planina Mts:** Chepan (Dragoman) (Borisova et al. 2005); **Eastern** **Stara Planina Mts:** Sliven [Forel 1892 (as Formica (Proformica) nasuta)]; **Thracian Lowland**: Pazardzhik [Forel 1892 (as Formica (Proformica) nasuta)]; **Bakadzhik-Burgas district**: Aytos [Forel 1892 (as Formica (Proformica) nasuta)]; **Belasitsa Mt.** [Atanassov 1964 (as Formica (Proformica) nasuta)]; **Krupnik-Sandanski-Petrich Valley**: Kresna gorge, Kozhuh Mt. (Atanassov and Dlusskij 1992); **Dupnitsa Basin**: Dupnitsa [Forel 1892 (as Formica (Proformica) nasuta)]; **Rila Mt.** [Forel 1892 (as Formica (Proformica) nasuta), Atanassov and Dlusskij 1992]; **Pirin Mt.** (Atanassov and Dlusskij 1992); **Eastern Rhodopi Mts:** Momchilgrad, Senoklas vill. (Madzharovo) (Lapeva-Gjonova 2004a).

#### Genus Cataglyphis

Förster, 1850

##### Cataglyphis aenescens

(Nylander, 1849)

Myrmecocystus cursor  Fonsc. var. *aenescens* Nyl.: Atanassov 1936, 1964

###### Records

(Map 73): **Bulgaria** [Atanassov 1936 (as Myrmecocystus cursor var. aenescens)]; **Eastern Danubian Plain**: Dunav river valley (lower reaches) (Atanassov and Dlusskij 1992); **Central** **Predbalkan**: Vit river valley (middle reaches) (Atanassov and Dlusskij 1992); **Eastern** **Stara Planina Mts:** Sliven [Forel 1892 (as Myrmecocystus cursor)]; **Ihtimanska Sredna Gora Mts:** Benkovski peak [Atanassov 1934 (as Myrmecocystus cursor)]; **Bakadzhik-Burgas district**: Aytos [Forel 1892 (as Myrmecocystus cursor)]; **Strandzha Mt.:** [Atanassov 1934 (as Myrmecocystus cursor)]; **Krupnik-Sandanski-Petrich Valley**: west of Petrich, along Strumeshnitsa river, Kozhuh Mt. [Atanassov 1964 (as Myrmecocystus cursor var. aenescens)]; **Western Rhodopi Mts:** Peshtera [Atanassov 1934 (as Myrmecocystus cursor)]; **Black Sea coast** (Atanassov and Dlusskij 1992); **Southern Black Sea coast**: Pomorie, Burgas, Sozopol, Veselie vill. [Forel 1892 (as Myrmecocystus cursor)].

###### Notes:

Forel (1892) and Atanassov (1934) recorded Cataglyphis cursor (as Myrmecocystus cursor) from Bulgaria; the species distribution in Mediterranean region runs from Iberian peninsula to Greece (Agosti 1990, Radchenko 2007). We suggest that the occurence of Cataglyphis cursor (which can hardly be distinguished from Cataglyphis aenescens) is rather improbable, and old records of this species should be considered as belonging to Cataglyphis aenescens.

##### Cataglyphis bicolor rufiventris

Emery, 1925

###### Records

(Map 73): **Thracian Lowland**: Pazardzhik (Agosti 1990); **Western Rhodopi Mts:** Asenovgrad (Agosti 1990).

###### Notes:

This taxon was omitted by Atanassov and Dlusskij (1992). Taxonomic status of this subspecies is not properly resolved and we cannot exclude the possibility that this name is junior synonym of Cataglyphis nodus.

##### Cataglyphis lividus

(André, 1881)

Cataglyphis livida bulgarica  Atanassov, 1982 (recorded by all authors, below).

###### Records

(Map 74): **Eastern Rhodopi Mts:** Odrintsi vill. (Ivaylovgrad) (Atanassov 1982, Agosti 1990, Atanassov and Dlusskij 1992), Mandritsa vill., Gugutka vill., Svirachi vill. (Atanassov and Dlusskij 1992).

##### Cataglyphis nodus

(Brullé, 1833)

Myrmecocystus viaticus  Fab. var. *megalocola* Foerst.: Forel 1892

###### Records

(Map 74): **Bulgaria** (Agosti and Collingwood 1987a, Atanassov and Dlusskij 1992); **Central Predbalkan**: Dermantsi vill. (Lukovit), Balgarski izvor vill. (Teteven), Lukovit [Atanassov 1936 (as Myrmecocystus viaticus)]; **Central Stara Planina Mts:** Shipka pass (Csősz and Markó 2005); **Eastern** **Stara Planina Mts:** Sliven [Forel 1892 (as Myrmecocystus viaticus Fab. var. *megalocola*)], along Belenska river [Atanasov 1936 (as Myrmecocystus viaticus)]; **Zemen Gorge**: Skakavitsa station (Kyustendil) [Atanassov 1936 (as Myrmecocystus viaticus)]; **Sofia Basin**: surroundings of Sofia (Antonova and Penev 2006); **Podbalkan Basins**: Rose valley (Atanassov et al. 1955); **Ihtimanska Sredna Gora Mts:** Benkovski peak (Atanassov 1934); **Lozenska Planina Mts** (Vassilev and Evtimov 1973): north of Pasarel vill. (Antonova and Penev 2008); **Thracian Lowland**: Pazardzhik [Forel 1892 (as Myrmecocystus viaticus Fab. var. *megalocola*)], Svilengrad,Ognyanovo (Atanassov and Vassileva 1976, Hubenov et al. 1998), Plovdiv [Atanassov 1936 (as Myrmecocystus viaticus)]; **Bakadzhik-Burgas district**: Aytos [Forel 1892 (as Myrmecocystus viaticus Fab. var. *megalocola*)]; **Strandzha Mt.** (Antonova et al. in press): Papia peak [Atanassov 1936 (as Myrmecocystus viaticus)]; **Osogovska Planina Mt.:** Osogovo hut (Kyustendil) [Atanassov 1936 (as Myrmecocystus viaticus)]; **Ograzhden Mt.** [Atanassov 1964 (as Myrmecocystus viaticus)]; **Boboshevo-Simitli Valley**: Kocherinovo [Forel 1892 (as Myrmecocystus viaticus Fab. var. *megalocola*)]; **Krupnik-Sandanski-Petrich Valley**: west of Petrich, along Strumeshnitsa river, around Marikostino vill., Marino pole vill. [Atanassov 1964 (as Myrmecocystus viaticus)]; **Pirin Mt.:** Vihren (Csősz and Markó 2005); **Slavianka Mt.** [Atanassov 1934 (as Myrmecocystus viaticus)]; **Western Rhodopi Mts:** Asenovgrad [Forel 1892 (as Myrmecocystus viaticus Fab. var. *megalocola*)], Peshtera [Atanassov 1934 (as Myrmecocystus viaticus Fab. and Myrmecocystus viaticus Fab. var. *megalocola*)]; **Eastern Rhodopi Mts:** Momchilgrad, Madzharovo, between Dabovets vill. and Kamilski dol vill. (Ivaylovgrad) (Lapeva-Gjonova 2004a); **Northern Black Sea coast**: Evksinograd palace (Atanassov 1936); **Southern Black Sea coast**: Veselie vill., Sozopol [Forel 1892 (as Myrmecocystus viaticus Fab. var. *megalocola*)].

###### Notes:

Atanassov and Dlusskij (1992) without any comments referred to records of Myrmecocystus viaticus var. megalocola (Forel 1892, Atanassov 1934), and Cataglyphis viaticus (Atanassov 1934, 1936, 1964, Atanassov et al. 1955, Vassilev and Evtimov 1973) to Cataglyphis nodus. The first name is now considered as a junior synonym of Cataglyphis bicolor (Förster) – the species is distributed mostly in North Africa and probably in the Middle East. Cataglyphis viaticus is definitely known from Iberian Peninsula and North Africa. At the same time, in Balkans, lives single species from the *bicolor* species-group: Cataglyphis nodus. As the taxonomy of this genus was studied insufficiently previously to the last two decades, all old records of Cataglyphis bicolor and Cataglyphis viaticus for Bulgaria most probably belong to Cataglyphis nodus.

#### Genus Polyergus

Latreille, 1804

##### Polyergus rufescens

(Latreille, 1798)

###### Records 

(Map 75): **Bulgaria** (Agosti and Collingwood 1987a, Atanassov and Dlusskij 1992); **Central Stara Planina Mts:** Brestnitsa vill. (Teteven) (Atanassov 1936); **Sofia Basin**: Sofia (Atanassov 1934, Antonova 2005, Antonova and Penev 2006), the surroundings of Sofia (Antonova and Penev 2006); **Podbalkan Basins**: Rose valley (Atanassov et al. 1955); **Ihtimanska Sredna Gora Mts:** Momin prohod, Benkovski peak (Atanassov 1934); **Strandzha** **Mt.:** Valchanov most loc. (Malko Tarnovo) (Atanassov 1936); **Ograzhden Mt.** (Atanassov 1964); **Krupnik-Sandanski-Petrich Valley**: Kozhuh Mt., Marino pole vill. (Atanassov 1964); **Rila Mt.:** Rila monastery (Forel 1892).

## Discussion

### Analysis of the faunistic data

The current Catalogue was compiled based on the investigation of the available material, including that collected by the authors personally, and on the critical reconsideration of the existing publications concerning Bulgarian ants (more than 100 sources). As a result, we include here 163 ant species, belonging to 40 genera of 6 subfamilies:

Subfamily Amblyoponinae – 1 genus, 2 species

Subfamily Ponerinae – 3 genera, 5 species

Subfamily Proceratiinae – 1 genus, 2 species

Subfamily Myrmicinae – 21 genera, 77 species

Subfamily Dolichoderinae – 5 genera, 9 species

Subfamily Formicinae – 9 genera, 68 species

Nevertheless, several records seem doubtful and need confirmation. On the other hand, we assume that after revision of a couple of genera (e.g. Messor, Tetramorium, Temnothorax, Camponotus, etc.) the number of species might increase.

The number of species of the respective genera is as follows:

Genera: Cryptopone, Pyramica, Manica, Stenamma, Pheidole, Myrmecina, Monomorium, Solenopsis, Formicoxenus, Harpagoxenus, Chalepoxenus, Anergates, Dolichoderus, Liometopum, Linepithema, Prenolepis and Polyergus – 1;

Genera: Amblyopone, Hypoponera, Ponera, Proceratium, Leptothorax, Myrmoxenus, Strongylognathus, Tapinoma, Plagiolepis and Lepisiota – 2;

Genera: Aphaenogaster, Crematogaster, Cardiocondyla, Bothriomyrmex and Cataglyphis – 4;

Genus: Proformica – 5;

Genus: Tetramorium – 6;

Genus: Messor – 7;

Genus: Camponotus – 14;

Genus: Myrmica – 15;

Genus: Formica – 18;

Genus: Temnothorax – 20;

Genus: Lasius – 21.

Compared with the “Fauna of Bulgaria” (Atanassov and Dlusskij 1992), this catalog contains 51 more ant species and summarizes the published data for all 163 species, found in Bulgaria.

In the monograph of Atanassov and Dlusskij (1992) 27 species, recorded for Bulgaria before 1992, are missed. Some of them are included as synonyms or they figure in the determination keys. Four of them, Proceratium numidicum, Messor concolor, Temnothorax nigriceps and Cardiocondyla nigra, were recorded for Bulgaria without exact localities till 1992. Lasius myops was conidered by Atanassov and Dlusskij (1992) as junior synonym of Lasius flavus and believed that previous records of Proformica nasuta for Bulgaria belong to Proformica striaticeps (see also Notes to Proformica nasuta in Catalogue).

Atanassov and Dlusskij (1992) discussed that it is possible to find some ant species on the Bulgarian territory – Temnothorax madeirense (as Tapinoma ambiguum), Crematogaster scutellaris, Lasius distinguendus and Lepisiota splendens (as Acantholepis splendens). The last two are included into the determination keys as well.

The presence of genera Chalepoxenus, Myrmoxenus and Harpagoxenus in Bulgaria was suggested by Atanassov and Dlusskij (1992), and these three genera are included only into the key for genera.

On the other hand, 25 species were additionally recorded to Bulgaria from 1992 till 2009 (2010 part); these are:

Ponera testacea (Csősz and Seifert 2003)

Pyramica baudueri (Bezděčka and Bezděčková 2009)

Myrmica constricta (Seifert et al. 2009)

Myrmica lonae (Seifert 2000b)

Myrmica vandeli (Stankiewicz and Antonova 2005)

Messor capitatus (Markó and Csősz 2002)

Crematogaster auberti (Lapeva-Gjonova in press (b))

Harpagoxenus sublaevis (Antonova 2009)

Temnothorax crassispinus (Seifert 1995)

Temnothorax graecus (MIZ - Warsaw, Poland, unpublished data)

Temnothorax cf. korbi (MIZ - Warsaw, Poland, unpublished data)

Temnothorax saxonicus (Seifert 1995)

Temnothorax sordidulus(Seifert 2006)

Temnothorax tauricus (Radchenko 1994, 1995a)

Myrmoxenus gordiagini (Buschinger and Douwes 1993)

Myrmoxenus ravouxi (Buschinger and Douwes 1993)

Tetramorium diomedeum (Csősz and Schulz 2010)

Tetramorium moravicum (Steiner et al. 2005)

Bothriomyrmex menozzii (Csősz and Markó 2005)

Lasius neglectus (Seifert 2000a)

Lasius paralienus (Seifert 1992)

Lasius platythorax (Antonova and Penev 2006)

Lasius psammophilus (Antonova and Penev 2006)

Camponotus aegaeus (Lapeva-Gjonova in press (b))

Camponotus gestroi (Lapeva-Gjonova in press (b))

Till now, several species were recorded for Bulgaria without defined localities, and we did not find them in any investigated collections:

Proceratium numidicum (Agosti and Collingwood 1987a)

Temnothorax nigriceps (Agosti and Collingwood 1987a)

Cardiocondyla nigra (Agosti and Collingwood 1987a, Radchenko 1995b)

Lepisiota splendens (Agosti and Collingwood 1987a)

Lasius meridionalis (Agosti and Collingwood 1987a, Atanassov and Dlusskij 1992).

Finally, we propose to exclude from the Bulgarian myrmecofauna two species, recorded earlier.

Tetramorium meridionale Emery, 1870 was recorded for Vitosha Mt. by Atanassov (1952). Based on the modern data, this somewhat enigmatic species is distributed in the West Mediterranean region (Iberian, southern France, Italy). It was recorded also for Crimea and even for Ural region (Nasonov 1889, Ruzsky 1905), but these records seem doubtful (see Radchenko 1992a, b, Sanetra et al. 1999).

Formica fuscocinerea Forel, 1874 was considered by most authors for many tens of years as a mountain Central European and Balkans subspecies of Formica cinerea (e.g. see Dlussky 1967), but Seifert (2002b) considered it as a good species, which is locally distributed in the Alps and Northern Apennines.

### Comparative analysis of the ant fauna of the different regions of Bulgaria

**Map 76.**
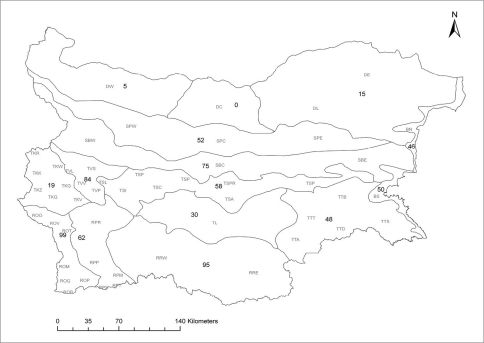
Total number of ant species for separate regions and subregions

We compare ant faunas of the natural regions of Bulgaria, as proposed by Hubenov (1997) (Map 1, Map 76 and Appendix III). Different regions of the country, and even different parts of a single region, have been studied to a variable extent, and there are many places where the ant fauna has been investigated insufficiently. Thus, currently we have no possibility to make a proper and detailed comparison of the ant faunas and their similarity across different regions, however our data might be useful for planning further investigations regarding the distribution of ants in Bulgaria.

The best studied area is the Rilo-Rhodopi region with 143 species from ca. 120 localities. The subregions Middle Struma Valley (line Krupnik-Sandanski-Petrich, 76 species) and Rhodopi Mountains (95 species) are also well studied areas with high species richness.

At the same time, Golo Bardo (1 species), Verila (5), Viskyar (5), Sushtinska (5) and Surnena (2) Sredna Gora mountains, Sturgach (1), Konyavska mountain (1), Sakar (3), Tundzha valley (2), Mesta valley (5) and Boboshevo-Simitli valley (7) are poorly studied and only a few ant species are recorded. There are almost no data for Central Danubian Plain, Ruy, Maleshevska, Zemenska and Vlahina mountains.

Despite the fact that the Transitional region is a larger and more diverse topographically, as well as climatically than the Rilo-Rhodopi region, only 115 species are reported from there. Most probably, this is the result of lower level of the myrmecological investigation carried out there. Only Sofia Basin (62 species), Vitosha (48), Plana (50), Lozenska (40) and Strandzha (40) mountains are sufficiently studied. Almost all information on myrmecofauna of Vitosha subregion arises from investigations in the following areas (Sofia Basin, Vitosha and Plana) and accordingly the greatest number of species have been reported there – 84. The worst studied area is the Kraishte-Konyavo subregion from the Transitional region, where only 19 ant species from just 8 localities are known, and 7 of these localities are in Zemen gorge.

The number of ant species recorded from the Stara Planina Range system is similar to that of Transitional region. The total number found in both subregions (Predbalkan and Stara Planina) is 88 species, 76 of which are given for Stara Planina Mountains only.

The territory of the Black Sea coast region is the smallest among all natural regions of Bulgaria and it is sufficiently studied: 75 ant species from ca. 27 localities are recorded.

Contrary to this, only 21 species are recorded from the Danubian Plain, which has an area of 31 523 square kilometers.

In general, we may consider that the ant fauna of Bulgaria is quite rich, which is confirmed by its comparison with adjacent regions.

Thus, 103 ant species from 34 genera are recorded for Romania (Markó et al. 2006), although the authors expect more species will be found in the country in the future. 140 species are known from Slovenia and 129 – from Croatia (Bračko 2006, 2007). The fauna of Serbia and Montenegro contains totally 160 species, 133 of which are found in Montenegro (Karaman 1998, 2004, Petrov 2004, 2006, 2008); 78 species are known from Macedonia (Doflein 1920, Petrov 1994, Karaman 2002, 2009). Agosti and Collingwood (1987a) recorded for Thracian part of Turkey 90 species, while more correct data of Çamlitepe and Aktaç (1987) and Aktaç et al. (1994) include 76 species. Finally, Greek and Turkish faunas are estimated at about 300 ant species each (Aktaç 1976, Petrov 2006, Kiran and Aktaç 2006, 2007, our unpublished data).

Finally, we may conclude: the Bulgarian ant fauna is rich and adequately studied as a whole, while several regions of the country require additional investigations. Such poorly myrmecologically elaborated areas are the Danubian Plain, Thracian Lowland, Kraishte-Konyavo and Tundzha-Strandzha subregions; moreover, ants of the Central Danubian Plain, Ruy, Maleshevska, Zemenska and Vlahina mountains have not been studied at all.

In general, Bulgarian ant fauna is richer than that of Romania, similar by the species number to those of many former Yugoslavian countries, but essentially poorer than faunas of Greece and Turkey. This fact is not surprising, regarding the geographical location and landscapes of Bulgaria. Furthermore, the core of Bulgarian myrmecofauna is composed by South European elements, but not Mediterranean species, and it is more closely related to the faunas of northern territories than to Greek fauna.

### Conservation status of the species

Totally, 13 ant species found in Bulgaria, are included in IUCN Red List of Threatened Species. Five of them (Anergates atratulus, Chalepoxenus muellerianus, Myrmoxenus ravouxi, Myrmoxenus gordiagini and Strongylognathus kratochvili) are placed to the category Vulnerable D2: “Population is characterized by an acute restriction in its area of occupancy (typically less than 100 km2) or in the number of locations”. Formicoxenus nitidulus and Harpagoxenus sublaevis are also Vulnerable, but in A2c category: “A reduction of at least 20%, projected or suspected to be met within the next ten years or three generations, whichever is the longer, based on a decline in area of occupancy, extent of occurrence and/or quality of habitat”. The status of another five species (Formica aquilonia, Formica lugubris, Formica polyctena, Formica pratensis and Formica rufa) is Lower Risk/near threatened: “Taxa which do not qualify for Conservation Dependent, but which are close to qualifying for Vulnerable”. Formica rufa is included in the Annex 2 and 3 of the Bulgarian Biodiversity Act (2002) as well (protected on the entire territory of the country). Temnothorax recedens is cited in the category Lower Risk/least concern: “Taxa which do not qualify for Conservation Dependent or Near Threatened”.

The IUCN’s classifications, based on the sizes of geographic ranges or the patterns of habitat occupancy, is complicated by problems of spatial scale. All of the above mentioned species need their conservation status at national level up-dated. We do not have enough data yet about the distributions and populations of these species in Bulgaria or on how their status in Bulgaria relates to their global population. Further investigations and accurate mapping would supply data that would clarify the situation.

## Supplementary Material

XML Treatment for Amblyoponinae

XML Treatment for Ponerinae

XML Treatment for Proceratiinae

XML Treatment for Myrmicinae

XML Treatment for Dolichoderinae

XML Treatment for Formicinae

## Appendix I: Index

**Table:** The Index includes names of good species and genera, mentioned in the current Catalogue. The page number in bold refers to the main treatment of the taxon. Page numbers in italics refer to the Maps.

*acervorum***20**, *92*, 119	*gibbosa***13**, *85*, 118	*pharaonis***19**, 65, *90*, 119
*aegaeus***41**, 61, *107*, 122	*gibbus***33**, *100*, 121	*Pheidole***16**, 60, *88*, 119
*aenescens***57**, *114*, 123	*glauca***50**, *110*, 123	*picea***51**, *111*, 123
*aethiops***41**, *107*, 122	*gordiagini***26**, 61, 63, *91*, 120	*piceus***45**, *108*, 123
*affinis***21**, *92*, 119	*graecus* 1, 3, **22**, 61, *93*, 119	*pilosiscapus***56**, *114*, 123
*alienus***35**, 36, 41, *103*, 121	*graminicola***17**, *89*, 119	*Plagiolepis***34**, 60, *101*, 121
*Amblyopone***4**, 5, 60, *79*, 117	*Harpagoxenus***20**, 60, 61, 63, *91*, 119	*platythorax* 40, **41**, 61, *106*, 122
*Anergates***27**, 60, 63, *96*, 120	*hellenica***8**, *82*, 117	*polyctena***51**, 63, *111*, 123
*Aphaenogaster***13**, 60, *85*, *86*, 118	*herculeanus***43**, *107*, 122	*Polyergus***59**, 60, *115*, 124
*aquilonia***47**, 63, *109*, 123	*humile***33**, *101*, 121	*Ponera***5**, 6, 60, *80*, 117
*atanassovii***14**, *86*, 118	*hungaricum***29**, 30, *98*, 121	*pratensis***52**, 63, *112*, 123
*atratulus***27**, 63, *96*, 120	*Hypoponera***5**, 60, *80*, 117	*Prenolepis***35**, 60, *102*, 121
*auberti***17**, 61, *89*, 119	*impressifrons***5**, *79*, 117	*pressilabris***53**, *112*, 123
*balcanicus* 2, **36**, *103*, 121	*interruptus***22**, *93*, 119	*Proceratium***6**, 60, 61, *81*, 117
*barbarus***14**, *86*, 119	*ionius***44**, *108*, 122	*Proformica***56**, 60, *113*, *114*, 123
*baudueri***7**, 60, *81*, 117	*jensi***39**, *105*, 121	*psammophilus* 36, **41**, 61, *106*, 122
*bicolor* rufiventris **57**, *114*, 123	*kobachidzei***56**, *113*, 123	*punctatissima***5**, *80*, 117
*bicornis***36**, *103*, 121	*korbi* (Proformica) **56**, *113*, 123	*pygmaea***34**, *101*, 121
*Bothriomyrmex***33**, 60, 61, *100*, 121	*korbi* (Temnothorax) 1, 3, **22**, 61, *94*, 119	*Pyramica***7**, 60, *81*, 117
*brunneus***36**, *103*, 121	*kratochvili***30**, 63, *98*, 121	*quadripunctatus***31**, *99*, 121
*bulgarica* 2, **26**, *96*, 120	*Lasius* 2, **35**, 36, 37, 38, 39, 40, 41, 60, 61, 121, *103*, *104*, *105*, *106*, 122	*ravouxi***26**, 61, 63, *91*, 120
*bulgaricus* 2, **21**, *92*, 119	*lateralis***44**, *108*, 123	*recedens* 20, **24**, 63, *94*, 119
*caducus***14**, *87*, 119	*lemani***50**, *111*, 123	*rubida***7**, *81*, 117
*caespitum***27**, 28, 30, *97*, 120	*Lepisiota***34**, 35, 60, 61, *102*, 121	*rubra***9**, *82*, 118
*Camponotus***41**, 42, 43, 44, 45, 46, 59, 60, 61, *107*, *108*, *109*, 122, 123	*Leptothorax* 2, **20**, 60, *92*, 119	*rufa***53**, 63, *112*, 123
*capitatus***15**, 61, *87*, 119	*ligniperdus***44**, *108*, 123	*rufescens***59**, *115*, 124
*Cardiocondyla* 2, **26**, 27, 60, 61, *96*, 120	*Linepithema***33**, 60, *101*, 121	*rufibarbis***54**, *113*, 123
*carniolicus***37**, *104*, 121	*Liometopum***31**, 60, *99*, 121	*ruginodis***9**, *83*, 118
*Cataglyphis***57**, 58, 60, *114*, *115*, 123, 124	*lividus***58**, *115*, 124	*rugulosa***9**, *83*, 118
*Chalepoxenus***26**, 60, 63, *91*, 120	*lobicornis***8**, *82*, 117	*sabuleti* 9, **10**, *83*, 118
*chefketi***28**, *97*, 120	*lonae***8**, 9, 60, *82*, 117	*samius***46**, *108*, 123
*cinerea***47**, 61, *109*, 123	*lugubris***50**, 63, *111*, 123	*sanguinea***55**, *113*, 123
*citrinus***37**, *104*, 121	*madeirense***32**, 33, 60, *100*, 121	*saxonicus***24**, 61, *95*, 120
*clara***48**, *109*, 123	*Manica***7**, 60, *81*, 117	*scabrinodis***10**, *83*, 118
*clypeatus***21**, *93*, 119	*melanocephalus***23**, *94*, 119	*schencki***10**, *84*, 118
*coarctata***5**, 6, *80*, 117	*melinum***6**, *81*, 117	*schmidti***18**, *89*, 119
*communistus***33**, *100*, 121	*menozzii***33**, 61, *100*, 121	*scutellaris***18**, 60, 119
*concolor***15**, 60, *87*, 119	*meridionalis* (Bothriomyrmex) **33**, *100*, 121	*semiruber***24**, *95*, 120
*constricta***7**, 60, *82*, 117	*meridionalis* (Lasius) **39**, 61, 121	*slovaca***11**, *84*, 118
*corticalis***21**, *93*, 119	*Messor***14**, 15, 59, 60, 61, *86*, *87*, *88*, 118, 119	*Solenopsis***19**, 60, *90*, 119
*crassispinus***22**, 23, 61, *93*, 119	*microcephalum***31**, *99*, 121	*sordidula***18**, *89*, 119
*Crematogaster***17**, 18, 60, 61, *89*, 119	*mixtus***39**, *105*, 122	*sordidulus* 24, **25**, 61, *95*, 120
*Cryptopone***5**, 60, *79*, 117	*Monomorium***19**, 60, *90*, 119	*specioides***11**, *84*, 118
*cunicularia***48**, *110*, 123	*moravicum* 29, **30**, 61, *98*, 121	*splendens***35**, 60, 61, 121
*dalmaticus***42**, *107*, 122	*muellerianus***26**, 63, *91*, 120	*stambuloffii* 2, **27**, *96*, 120
*debile***12**, *85*, 118	*muscorum***20**, *92*, 119	*Stenamma***12**, 60, *85*, 118
*denticulata***4**, *79*, 117	*myops***39**, 60, *105*, 122	*striaticeps***56**, 60, *114*, 123
*diomedeum***29**, 61, *97*, 121	*Myrmecina***17**, 60, *89*, 119	*Strongylognathus***30**, 31, 60, 63, *98*, 121
*distinguendus***37**, 60, *104*, 121	*Myrmica***7**, 8, 9, 10, 11, 12, 60, 61, 70, *82*, *83*, *84*, 117, 118	*structor***15**, *88*, 119
*Dolichoderus***31**, 60, *99*, 121	*Myrmoxenus***26**, 60, 61, 63, *91*, 120	*sublaevis***20**, 61, 63, *91*, 119
*eduardi***5**, *80*, 117	*nadigi***23**, 67, *94*, 119	*subterranea***13**, *86*, 118
*elegans* 2, **26**, *96*, 120	*nasuta***56**, 60, *113*, 123	*sulcinodis***12**, *84*, 118
*emarginatus***37**, *104*, 121	*neglectus***39**, 61, *105*, 122	*sylvaticus***46**, *108*, 123
*epirotes***13**, *85*, 118	*niger***39**, 40, 41, *105*, 122	*Tapinoma***32**, 60, *100*, 121
*erraticum***32**, *100*, 121	*nigra***27**, 60, 61, 120	*taurica***34**, *101*, 121
*exsecta***48**, *110*, 123	*nigriceps***23**, 60, 61, 119	*tauricus***25**, 61, *95*, 120
*fallax***43**, *107*, 122	*nitens***35**, *102*, 121	*Temnothorax* 1, 2, 3, **20**, 21, 22, 23, 24, 25, 59, 60, 61, 63, *92*, *93*, *94*, *95*, 119, 120
*ferox***29**, *97*, 121	*nitidigaster* 2, **40**, *106*, 122	*testacea***6**, 60, *80*, 117
*flavus***38**, 39, 60, *104*, 121	*nitidulus***19**, 63, *91*, 119	*testaceus***31**, *98*, 121
*Formica***47**, 48, 49, 50, 51, 52, 53, 54, 55, 60, 61, 63, *109*, *110*, *111*, *112*, *113*, 123	*nodus* 57, **58**, 59, *115*, 124	*Tetramorium***27**, 28, 29, 30, 59, 60, 61, *97*, *98*, 120, 121
*Formicoxenus***19**, 60, 63, *91*, 119	*numidicum***6**, 60, 61, 117	*truncatus***46**, *109*, 123
*frauenfeldi***34**, *102*, 121	*nylanderi* 22, **23**, *94*, 119	*truncorum***55**, *113*, 123
*fugax***19**, *90*, 119	*ochracea***5**, *79*, 117	*tuberum* 23, **25**, *95*, 120
*fuliginosus***38**, *105*, 121	*oertzeni***15**, *87*, 119	*umbratus***41**, *106*, 122
*fusca***49**, *110*, 123	*pallida***13**, *86*, 118	*unifasciatus***25**, *95*, 120
*gagates***49**, *110*, 123	*pallidula***16**, *88*, 119	*vagus***46**, *109*, 123
*gallienii***8**, *82*, 117	*paralienus* 36, **40**, 61, *106*, 122	*vandeli***12**, 61, *84*, 118
*gestroi***43**, 61, *107*, 122	*parvulus***23**, *94*, 119	

## Appendix II: Distribution Maps

**Table:**

Name of Region					Abbreviations
Danube Plain	D				
Western			DW		
Central			DC		
Eastern			DE		
Ludogorie – Dobrudzha district		DL			
Stara Planina Range system	S				
Predbalkan			SP		
Western				SPW	
Central				SPC	
Eastern				SPE	
Stara Planina Mts (Balkans)			SB		
Western				SBW	
Central				SBC	
Eastern				SBE	
Transitional region	T				
Kraishte-Konyavo district			TK		
Ruy Mt.				TKR	
Golo Bardo Mt.				TKG	
Verila Mt.				TKV	
Kraishte				TKK	
Zemenska Planina Mt.				TKZ	
Konyavska Planina Mt.				TKQ	
Viskyar Mt.				TKW	
Vitosha district			TV		
Sofia Basin				TVS	
Lyulin Mt.				TVL	
Vitosha Mt.				TVV	
Plana Mt.				TVP	
Srednogorie-Podbalkan subregion			TS		
Podbalkan Basins				TSP	
Rose valley					TSPR
Sredna Gora Mts				TSS	
Ihtimanska Mt.					TSI
Lozenska Planina Mt.					TSL
Sushtinska					TSC
Surnena					TSA
Thracian Lowland subregion			TL		
Tundzha-Strandzha subregion			TT		
Sakar-Tundzha district				TTT	
Sakar Mt.					TTA
Bakadzik-Burgas district				TTB	
Strandzha-Dervent district				TTD	
Strandzha Mt.					TTS
Rila-Rhodopi Massif	R				
Osogovo-Belasitsa group			RO		
Osogovska Planina Mt.				ROO	
Vlahina Planina Mt.				ROV	
Maleshevska Planina Mt.				ROM	
Ograzhden Mt.				ROG	
Belasitsa Mt.				ROB	
Srednostrumska valley				ROS	
Boboshevo-Simitli valley					ROT
Krupnik-Petrich valley					ROP
Rila-Pirin group			RP		
Rila Mt.				RPR	
Pirin Mt.				RPP	
Slavianka Mt.				RPS	
Sturgach Mt.				RPT	
Mesta valley				RPM	
Rhodopi Mts			RR		
Western				RRW	
Eastern				RRE	
Black Sea coast	B				
Northern			BN		
Southern			BS		

## Appendix III: Table of species by regions and subregions in Bulgaria

**Table:**

Species	*I*			*II*						*III*																			*IV*											*V*	
					*a*			*b*			*c*						*d*				*e*					*f*	*g*			*h*					*i*				*j*		
		*1*	*2*	*3*	*1*	*2*	*3*	*1*	*2*	*3*	*4*	*5*	*6*	*7*	*8*	*9*	*10*	*11*	*12*	*13*	*14*	*15*	*16*	*17*	*18*		*19*	*20*	*21*	*22*	*23*	*24*	*25*		*26*	*27*	*28*	*29*			
																																	*A*	*B*					*1*	*2*	*30*	*31*
Amblyopone denticulata									●														●																		
Amblyopone impressifrons																									●								●								
Cryptopone ochracea			●																						●								●								
Hypoponera eduardi																																	●								
Hypoponera punctatissima			●													●						●											●								
Ponera coarctata			●													●												●		●			●					●	●	●	●
Ponera testacea																																	●								
Proceratium melinum			●																						●								●								●
Proceratium numidicum																																									
Pyramica baudueri																																							●		
Manica rubida					●			●									●							●					●					●		●		●			●
Myrmica constricta																																	●								
Myrmica gallienii				●													●																								
Myrmica hellenica																●									●															●	●
Myrmica lobicornis								●									●	●											●	●				●	●	●		●			
Myrmica lonae																●					●							●										●			
Myrmica rubra								●	●							●	●	●																				●			
Myrmica ruginodis																●	●	●												●			●	●		●		●			
Myrmica rugulosa							●	●	●							●					●				●									●				●	●		
Myrmica sabuleti				●			●									●	●	●			●							●						●	●			●	●		
Myrmica scabrinodis					●				●							●	●	●			●						●			●								●			
Myrmica schencki							●									●	●	●			●												●	●	●			●			
Myrmica slovaca																●												●										●			
Myrmica specioides																●			●									●						●							●
Myrmica sulcinodis							●									●	●	●	●											●			●	●				●			
Myrmica vandeli																	●																								
Stenamma debile	●						●		●							●		●				●								●								●	●		
Aphaenogaster epirotes			●																																				●		
Aphaenogaster gibbosa							●																										●			●			●	●	
Aphaenogaster pallida				●																																					
Aphaenogaster subterranea				●					●								●										●	●	●	●						●		●	●	●	
Messor atanassovii																									●			●									●	●			
Messor barbarus																									●		●							●						●	●
Messor caducus																								●	●					●	●		●						●	●	●
Messor capitatus																																								●	
Messor concolor																									●																
Messor oertzeni																				●										●	●		●			●		●	●	●	
Messor structor				●	●		●							●		●	●	●	●	●	●			●	●		●		●	●	●		●			●		●	●	●	●
Pheidole pallidula				●	●		●	●	●					●										●				●		●	●		●	●				●	●	●	●
Myrmecina graminicola				●												●		●							●								●		●			●			●
Crematogaster auberti																																	●								
Crematogaster schmidti							●		●															●	●		●	●		●			●			●		●	●		●
Crematogaster scutellaris																																									
Crematogaster sordidula									●																		●						●					●	●	●	
Monomorium pharaonis			●													●									●			●												●	●
Solenopsis fugax				●	●		●									●	●			●	●			●					●				●					●	●		●
Formicoxenus nitidulus					●		●										●												●	●						●		●			
Harpagoxenus sublaevis																																				●					
Leptothorax acervorum								●									●	●																●				●			
Leptothorax muscorum							●										●																	●		●		●			
Temnothorax affinis							●		●							●	●	●	●											●					●	●			●		
Temnothorax bulgaricus									●					●																			●					●		●	
Temnothorax clypeatus														●				●	●										●	●			●							●	
Temnothorax corticalis																	●																●							●	●
Temnothorax crassispinus																●		●																				●			
Temnothorax graecus								●																																	
Temnothorax interruptus				●			●		●									●															●							●	
*Temnothorax cf. korbi*																																									●
Temnothorax melanocephalus																																	●	●							
Temnothorax nadigi																																						●			
Temnothorax nigriceps																																									
Temnothorax nylanderi				●			●									●					●							●					●							●	●
Temnothorax parvulus							●		●								●	●																							
Temnothorax recedens									●																		●						●							●	●
Temnothorax saxonicus							●										●																●							●	
Temnothorax semiruber									●																					●		●	●					●	●		
Temnothorax sordidulus				●			●																																		
Temnothorax tauricus																																	●					●			
Temnothorax tuberum																●									●									●	●			●			
Temnothorax unifasciatus					●				●							●	●	●			●							●		●				●				●			●
Chalepoxenus muellerianus																																						●			
Myrmoxenus gordiagini				●																																					
Myrmoxenus ravouxi																																	●								
Cardiocondyla bulgarica																																			●					●	●
Cardiocondyla elegans																																	●		●					●	●
Cardiocondyla nigra																																									
Cardiocondyla stambuloffii									●																															●	●
Anergates atratulus							●										●												●									●		●	●
Tetramorium caespitum				●	●		●	●	●					●		●	●	●	●		●			●	●		●	●		●	●		●	●		●	●	●	●		●
Tetramorium chefketi			●				●									●	●				●				●			●		●		●			●				●		●
Tetramorium diomedeum																																									●
Tetramorium ferox																								●									●					●	●		●
Tetramorium hungaricum					●											●		●												●			●						●	●	
Tetramorium moravicum																●		●			●							●				●						●			
Strongylognathus kratochvili					●	●																																			
Strongylognathus testaceus																														●			●						●		●
Dolichoderus quadripunctatus					●											●		●									●	●		●			●	●				●	●		●
Liometopum microcephalum	●		●		●			●	●											●				●	●		●	●		●			●	●	●			●	●		●
Tapinoma erraticum				●			●	●	●							●	●	●			●							●		●			●	●	●			●	●	●	●
Tapinoma madeirense																		●																	●			●		●	
Bothriomyrmex communistus																												●					●								
Bothriomyrmex gibbus				●																																					
Bothriomyrmex menozzii																																								●	
Bothriomyrmex meridionalis																																	●						●		
Linepithema humile																●																								●	●
Plagiolepis pygmaea							●		●							●	●				●						●	●	●					●					●		●
Plagiolepis taurica				●			●									●		●			●									●	●		●					●			
Lepisiota frauenfeldi																														●			●						●		
Lepisiota splendens																																									
Prenolepis nitens																●								●				●		●			●					●	●		
Lasius alienus				●			●					●				●	●	●			●						●	●		●			●	●				●	●	●	●
Lasius balcanicus																●		●																	●			●		●	
Lasius bicornis																					●												●								
Lasius brunneus					●				●					●		●	●	●			●						●	●					●	●				●	●		●
Lasius carniolicus																									●								●								●
Lasius citrinus			●		●				●							●					●																	●		●	
Lasius distinguendus				●																																					
Lasius emarginatus			●	●	●				●												●							●		●			●					●	●	●	
Lasius flavus							●	●								●	●		●		●			●				●		●			●	●				●			
Lasius fuliginosus	●				●		●		●							●	●	●	●		●			●	●		●		●				●					●	●	●	
Lasius jensi																																						●			
Lasius meridionalis																																									
Lasius mixtus					●		●	●	●					●															●			●					●				
Lasius myops					●												●																							●	
Lasius neglectus			●																																					●	
Lasius niger				●	●		●		●					●		●	●	●			●			●						●			●	●				●			●
Lasius nitidigaster								●										●															●							●	●
Lasius paralienus																●		●			●							●												●	
Lasius platythorax																●		●			●																	●			
Lasius psammophilus																●		●																							
Lasius umbratus																●															●							●	●		
Camponotus aegaeus																																	●		●						
Camponotus aethiops				●			●		●							●	●	●		●	●			●	●		●	●		●	●		●	●		●		●	●		●
Camponotus dalmaticus									●											●					●					●			●	●		●		●	●		●
Camponotus fallax																●		●			●												●								
Camponotus gestroi																																	●								
Camponotus herculeanus							●									●	●	●		●								●		●			●	●	●			●			●
Camponotus ionius																																	●								
Camponotus lateralis					●				●							●	●				●							●		●						●		●	●		●
Camponotus ligniperdus					●		●							●		●	●	●		●	●			●						●				●	●			●	●	●	
Camponotus piceus				●					●							●	●	●			●				●		●	●		●			●	●					●		●
Camponotus samius																								●				●					●					●	●	●	
Camponotus sylvaticus																					●																				
Camponotus truncatus							●									●																	●					●	●		
Camponotus vagus					●		●	●						●		●	●	●	●	●	●			●	●			●	●	●			●			●		●	●	●	●
Formica aquilonia				●																														●							
Formica cinerea				●			●									●	●	●			●			●	●								●	●	●			●	●	●	
Formica clara																		●																						●	●
Formica cunicularia				●			●									●		●			●						●	●											●		●
Formica exsecta					●		●	●			●				●		●	●	●	●									●	●				●	●	●		●	●		
Formica fusca	●				●											●	●	●			●				●									●		●		●			
Formica gagates					●		●							●		●		●			●									●				●				●	●		●
Formica glauca					●			●																	●													●			
Formica lemani																	●													●								●			
Formica lugubris			●			●	●	●	●		●				●		●	●	●	●						●				●			●	●	●	●		●	●	●	
Formica picea								●	●																									●				●			
Formica polyctena	●								●	●																								●	●						
Formica pratensis			●		●		●	●	●		●			●	●	●	●	●	●		●		●	●		●		●	●	●			●	●	●		●	●	●	●	●
Formica pressilabris							●										●																	●				●			
Formica rufa			●		●	●	●	●	●		●			●	●	●	●	●	●	●	●					●		●		●			●	●	●	●		●	●	●	
Formica rufibarbis									●							●	●	●			●						●	●					●	●				●			●
Formica sanguinea	●				●		●		●		●				●	●	●	●	●									●	●					●				●	●		
Formica truncorum	●			●			●		●																				●				●					●		●	
Proformica kobachidzei																																●									
Proformica korbi																																●									
Proformica nasuta									●																●					●								●			
Proformica pilosiscapus																																	●				●				
Proformica striaticeps							●		●																●		●			●			●	●	●				●		
Cataglyphis aenescens			●		●				●											●							●	●					●					●			●
*Cataglyphis bicolor rufiventris*																									●													●			
Cataglyphis lividus																																							●		
Cataglyphis nodus					●			●	●					●		●				●	●			●	●		●	●	●		●	●	●		●	●		●	●	●	●
Polyergus rufescens								●								●				●				●				●			●		●	●							

**Regions and subregions used in the table:** **I** Danubian Plain **II** Stara Planina Range system **III** Transitional region **IV** Rilo-Rhodopi Massif **V** Black Sea coast **a** Predbalkan **b** Stara planina Mts (Balkan) **c** Kraishte-Konyavo subregion **d** Vitosha subregion **e** Srednogorie-Podbalkan subregion **f** Thracian Lowland **g** Tundzha-Strandzha subregion **h** Osogovo-Belasitsa group **i** Rila-Pirin group **j** Rhodopi Mts **1** western **2** central **3** eastern **4** Golo bardo **5** Verila **6** Konyavska Mt. **7** Zemenska Mt. **8** Zemen gorge **9** Viskyar **10** Sofia Basin **11** Vitosha Mt. **12** Plana Mt. **13** Lyulin Mt. **14** Ihtimaska Sredna Gora **15** Lozenska Mt. **16** Sushtinska Sredna Gora **17** Surnena Sredna Gora **18** Podbalkan **19** Sakar Mt. **20** Bakadzhik-Bourgas district **21** Strandzha Mt. **22** Osogovska Mt. **23** Belasitsa Mt. **24** Ograzhden Mt. **25** Middle Struma valley **26** Rila Mt. **27** Pirin Mt. **28** Slavyanka Mt. **29** Mesta valley **30** northern **31** southern **A** Boboshevo-Simitli **B** Krupnik-Sandanski-Petrich
